# Elucidating electrochemical nitrate and nitrite reduction over atomically-dispersed transition metal sites

**DOI:** 10.1038/s41467-023-40174-4

**Published:** 2023-07-28

**Authors:** Eamonn Murphy, Yuanchao Liu, Ivana Matanovic, Martina Rüscher, Ying Huang, Alvin Ly, Shengyuan Guo, Wenjie Zang, Xingxu Yan, Andrea Martini, Janis Timoshenko, Beatriz Roldán Cuenya, Iryna V. Zenyuk, Xiaoqing Pan, Erik D. Spoerke, Plamen Atanassov

**Affiliations:** 1 Department of Chemical and Biomolecular Engineering, University of California, Irvine, CA 92697 USA; 2 Department of Chemical and Biological Engineering, University of New Mexico, Albuquerque, NM 87131 USA; 3 Theoretical Division, Los Alamos National Laboratory, Los Alamos, NM 87545 USA; 4 Department of Interface Science, Fritz Haber Institute of the Max Planck Society, 4-6 Faradayweg, Berlin, 14195 Germany; 5 Department of Materials Science and Engineering, University of California, Irvine, CA 92697 USA; 6 Sandia National Laboratories, Albuquerque, NM 87185 USA

**Keywords:** Electrocatalysis, Chemical engineering, Electrocatalysis

## Abstract

Electrocatalytic reduction of waste nitrates (NO_3_^−^) enables the synthesis of ammonia (NH_3_) in a carbon neutral and decentralized manner. Atomically dispersed metal-nitrogen-carbon (M-N-C) catalysts demonstrate a high catalytic activity and uniquely favor mono-nitrogen products. However, the reaction fundamentals remain largely underexplored. Herein, we report a set of 14; 3*d*-, 4*d*-, 5*d*- and *f*-block M-N-C catalysts. The selectivity and activity of NO_3_^−^ reduction to NH_3_ in neutral media, with a specific focus on deciphering the role of the NO_2_^−^ intermediate in the reaction cascade, reveals strong correlations (R=0.9) between the NO_2_^−^ reduction activity and NO_3_^−^ reduction selectivity for NH_3_. Moreover, theoretical computations reveal the associative/dissociative adsorption pathways for NO_2_^−^ evolution, over the normal M-N_4_ sites and their oxo-form (O-M-N_4_) for oxyphilic metals. This work provides a platform for designing multi-element NO_3_RR cascades with single-atom sites or their hybridization with extended catalytic surfaces.

## Abstract (web summary)

Understanding of atomically dispersed metal-nitrogen-carbon (M-N-C) for electrochemical nitrate reduction is important and of high interest. Here, the authors employ a series of M-N-C catalysts to investigate selectivity correlation between nitrate and nitrite reduction.

## Introduction

Recently, significant effort has been focused to decarbonize the production of chemicals and fuels^1,2^. Among these, the decarbonization of ammonia (NH_3_) synthesis from the traditionally energy intensive and environmentally damaging Haber-Bosch (H-B) process remains a grand challenge^3,4^. The electrochemical synthesis of NH_3_, can ideally utilize renewable energy, reactive N-species and protons from water to generate NH_3_. The reduction of di-nitrogen (N_2_) in aqueous protic electrolytes remains the most heavily researched green pathway for NH_3_ synthesis^5^. However, due to the high bond dissociation energy of N_2_ (945 kJ mol^−1^), ultra-low solubility in aqueous electrolytes and competition from the hydrogen evolution reaction (HER), the current Faradaic efficiencies (FE) and NH_3_ yield rates (Yield_NH3_) remain prohibitively low^4,6^. Given the total lack of reproducibility and the difficulties of ubiquitous NH_3_ contamination in electrochemical systems, direct N_2_ reduction remains unproven in aqueous protic electrolytes^7^.

To circumvent the challenges of N_2_ reduction, there is a renewed interest in closing the N-cycle by recycling the reactive, more oxidized N-species (nitrate-NO_3_, nitrite-NO_2_ and nitric oxide-NO) to NH_3_^8–11^. The electrochemical NO_3_^−^ reduction reaction (NO_3_RR) is of particular interest due to its weaker N=O bond (204 kJ mol^−1^), large solubility in aqueous electrolytes and widespread availability. NO_3_^−^ is an environmental pollutant found in industrial waste streams and agricultural runoffs, due to the heavy overfertilization practices currently utilized^8^. While the NO_3_RR to NH_3_ provides a pathway for efficient recycling of NH_3_/NO_3_^−^ (originating from the H-B process), additional efforts are focusing on low energy plasma techniques to produce NO_3_^−^ from air, but such processes are to date, not energy efficient^12–14^.

The electrochemical NO_3_RR to NH_3_ is a complex 8e^−^ (9H^+^) transfer process, involving several desorbable and non-desorbable surface intermediates, creating a challenge for tailoring the catalytic surface for favorable adsorption of specific intermediates due to scaling relations^15–17^. Previous attempts were made to tune the intermediate adsorption binding energies by modulating the electronic structure on extended metal surfaces through alloys such as CuNi, PdAu, and PtRu^18–20^. These attempts achieved improved FE_NH3_ and Yield_NH3_ compared to mono-metallic counterparts, however, these approaches are still constrained by scaling relations and combat this by utilizing strongly alkaline or acidic environments with large concentrations of NO_3_^−^.

In the biological NO_3_RR, the reaction is partitioned as an enzymatic cascade, where the initial 2e^−^ reduction of NO_3_^−^-to-NO_2_^−^ is catalyzed over a Mo-cofactor in nitrate-reductase and the further reduction of NO_2_^−^ to NH_3_/N_2_ is catalyzed over an Fe- or Cu-cofactor in nitrite-reductase^21–23^. Inspired by the enzymatic pathway, recent work demonstrated that an atomically dispersed bi-metallic FeMo-N-C catalyst could successfully partition the NO_3_RR into the NO_3_^−^-to-NO_2_^−^ and NO_2_^−^-to-NH_3_ constituents, outperforming its mono-metallic Mo-N-C or Fe-N-C counterparts. The atomically dispersed Mo-N_x_ sites facilitated dissociative adsorption of NO_3_^−^, releasing NO_2_^−^, which is subsequently re-adsorbed and reduced to NH_3_ over Fe-N_x_ sites at 100% FE^24^. Additionally, other approaches to partition the NO_3_RR are emerging in the field for example, employing heterogenous metal sites, where each metal is tailored to efficiently catalyze a segment of the reaction. A recent study demonstrated potential dependent phase transitions of a Cu-Co (3–5 nm) binary metal sulfide catalyst, in which the inner Cu/CuO_x_ phases efficiently reduce NO_3_^−^-to-NO_2_^−^, while the outer Co/CoO phases selectively reduce the NO_2_^−^-to-NH_3_. The latter results in a NO_3_RR cascade to NH_3_, reaching a high FE of 90.6%^25^. A recent complementary work examining a series extended of transition metal surfaces by Carvalho et al. described NO_3_RR catalyst design parameters by linking the electronic structure to experimentally observed NO_3_RR performance^26^. These design parameters are derived from microkinetic models that described the 2e^−^ transfer rate limiting step (NO_3_^−^-to-NO_2_^−^) and further the NO_3_RR FE_NH3_, identifying a competitive adsorption between H^+^ and NO_3_^−^, that was described by the material-dependent property $({\Delta {{{{{\rm{G}}}}}}}_{{{{{{{\rm{H}}}}}}}^{*}}-{\Delta {{{{{\rm{G}}}}}}}_{{{{{{{\rm{NO}}}}}}}_{3}^{-*}})$. The study found that increasing FE_NH3_ correlates with NO* binding and subsequent dissociation into N* and O* as the d-band energy approaches the Fermi energy, resulting in the Co-foil showing the highest ammonium selectivity.

Atomically dispersed metal-nitrogen-carbon (M-N-C) catalysts have been extensively studied in neighboring electrocatalytic reactions (carbon dioxide reduction-CO_2_RR^27–29^, oxygen reduction-ORR^30,31^), demonstrating excellent catalytic activities and unique reaction pathways. Recently, atomically dispersed Fe-N-C catalysts were shown to be very selective for the NO_3_RR to NH_3_^32,33^, owing to their maximized atomic utilization and favorable NO_3_^−^ adsorption over H^+^. Additionally, isolated active sites provide an unfavorable environment for coupling adsorbed N-molecules, enabling mono N-based products (e.g., NH_3_) to be selectively produced.

The current understanding of atomically dispersed M-N-C catalysts for the complex NO_3_RR is very limited, with only M = Fe, Mo, and Cu being explored at varying conditions. Herein, we identify key transition metals and rare earth elements, important for electrocatalytic reactions and synthesize a set of 13 atomically dispersed M-N-C catalysts (M = Cr, Mn, Fe, Co, Ni, Cu, Mo, Ru, Rh, Pd, W, La, and Ce). The reaction onset potential (HER, NO_3_RR and NO_2_RR), the NO_3_RR selectivity to both NO_2_^−^ and NH_3_ and the NO_2_RR selectivity to NH_3_, elucidate several experimental activity descriptors. Isotopically doped ^15^NO_2_^−^ in the NO_3_RR highlights the complex production/consumption mechanism of the NO_2_^−^ intermediate. Density functional theory (DFT) evaluates the Gibbs free energy for both the NO_3_RR and NO_2_RR following either the dissociative adsorption or associative adsorption pathway. Relating the experimentally determined and computationally determined activity descriptors, reveals strong correlations for the NO_2_RR (*R* = 0.72) and the NO_3_RR (*R* = 0.73) selectivity to NH_3_. This work bridges the gap between computation and experiment for the NO_3_^−^ and NO_2_^−^ reduction reactions over atomically dispersed M-N-C catalysts by providing a powerful set of activity descriptors that correlate strongly with the experimentally observed activity. These descriptors can be utilized to guide future atomically dispersed M-N_x_ catalyst development, for highly active and efficient NO_3_^−^ reduction to NH_3_.

## Results

### Structural environment of single-atom metal centers

**Fig. 1:**
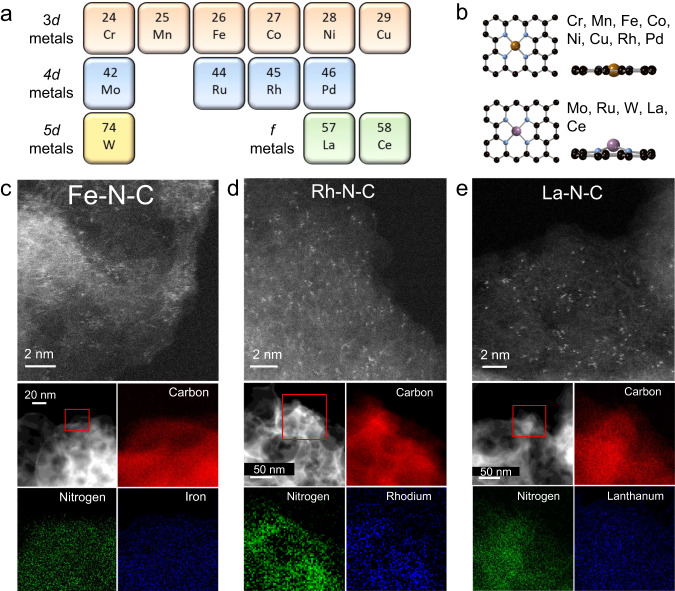
Physical structure of the atomically dispersed transition-metal catalysts. **a** Selection of 3*d*, 4*d*, 5*d* and *f* metals synthesized via the sacrificial support method. **b** Schematic of the nitrogen coordinated metal active site (M-N_4_) on a prototype carbon matrix, illustrating in-plane and out-of-plane configurations. Where the black, blue, brown/purple balls represent carbon, nitrogen and metal atoms, respectively. **c**–**e** Representative atomic resolution HAADF STEM images for the Fe-N-C, Rh-N-C and La-N-C catalysts. High contrast points indicate atomically dispersed metal sites. Corresponding EDS mapping of the M-N-C is given below.

Atomically dispersed M-N-C catalysts were synthesized through the well-established sacrificial support method (SSM) originally developed by our group^34–36^. The SSM has been extensively applied for atomically dispersed Fe-N-C catalysts for the ORR and CO_2_RR^27,37^. Here, extending beyond Fe-N-C to a variety of 3*d-*, 4*d-*, 5*d-* and *f-*block metals, a set of atomically dispersed M-N-C catalysts (M = Cr, Mn, Fe, Co, Ni, Cu, Mo, Ru, Rh, Pd, La, Ce, and W) were synthesized, as shown in Fig. 1a. The metallic centers ideally have a first coordination shell of 4-nitrogen atoms and are coordinated in either an in-plane or out-of-plane configuration as shown in Fig. 1b. With the synthesis conditions being adjusted to maintain the atomically dispersed nature for each metal element (Table S1), the resulting catalysts showed consistent porosity and pore size distribution (Fig. S1), degree of graphitization (Fig. S2) and a well-maintained metal loading at 0.5–1.5 wt% (Fig. S3).

X-ray diffraction (XRD) shows only the characteristic (002) and (100) peaks for carbon, confirming the absence of bulk-like ordered metallic phases (Fig. S4). Figure 1c–e shows representative aberration corrected high-angle annular dark-field scanning transmission electron microscopy (AC-HAADF-STEM) images for the Fe, Rh, and La-N-C, where atomically dispersed metal sites are clearly observed through the high contrast points. Energy dispersive X-ray spectroscopy (EDS) maps confirm the homogenous distribution of carbon, nitrogen, and the relevant metal throughout the catalyst, Fig. 1c–e (see other M-N-Cs in Figs. S5–S8). Throughout the main text physical characterization of the Fe-, Rh- and La-N-C are shown as a 3*d*, 4*d* and *f*-metal representative for the set of M-N-C catalysts. Complete characterization for the remaining M-N-Cs is found in the ESI as noted.

**Fig. 2:**
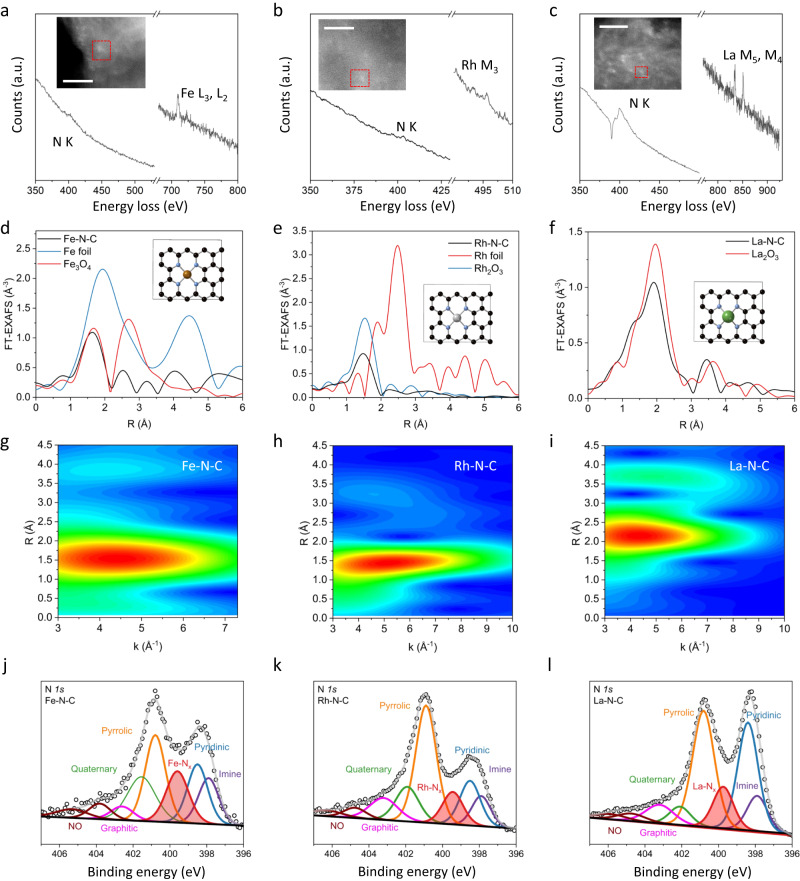
Coordination environment of the single-atom metal centers. **a**–**c** Atomic resolution EELS point spectra of the N K-edge and corresponding metal-edge of Fe (L_3,2_), Rh (M_3_) and La (M_5,4_), in the M-N-C catalysts, indicating nitrogen coordination of the single atom M-N_x_ site. All scale bars are 2 nm. **d**–**f** FT EXAFS spectra of the Fe, Rh and La-N-C catalysts, with the corresponding metallic foil and metal oxide standards, demonstrating the presence of M-N_x_ sites. Where the black, blue, brown/silver/green balls represent carbon, nitrogen and metal atoms, respectively. **g**–**i** WT-EXAFS of the Fe K-edge of Fe-N-C, Rh K-edge of Rh-N-C and La L_3_-edge of La-N-C. **j**–**l** N 1*s* XPS spectra for the Fe, Rh and La-N-C catalysts, confirming the presence of M-N_x_ moieties.

**Fig. 3:**
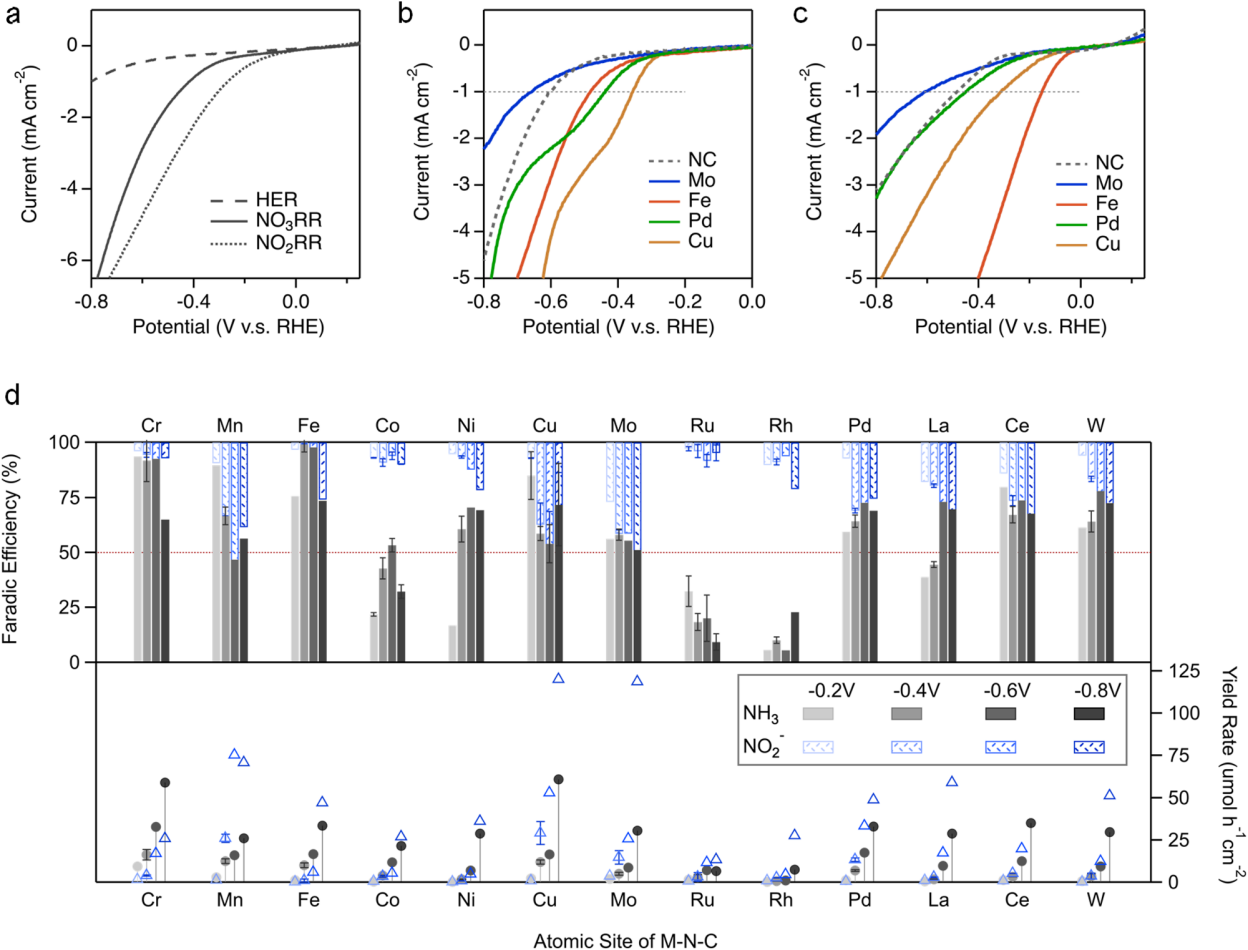
Electrochemical activation, selectivity, and activity for the NO_3_RR and NO_2_RR. **a** Example of a linear sweep voltammetry (LSV) curve for the Cr-N-C catalyst in electrolytes of 0.05 M PBS (HER), 0.05 M PBS + 0.01 M KNO_2_ (NO_2_RR) and 0.05 M PBS + 0.16 M KNO_3_ (NO_3_RR). Representative LSV over the metal free N-C and a variety of 3*d* and 4*d* metals for the (**b**) NO_3_RR and (**c**) NO_2_RR. Complete LSV for all examined M-N-C and metal free catalysts are given in Fig. S28. **d** Gap analysis plot (GAP) for the electrochemical NO_3_RR for all M-N-C catalysts in 0.05 M PBS + 0.16 M KNO_3_ for 2 h. The top section of the figure shows the Faradaic efficiency for NO_2_^−^ (blue; top-down) and NH_3_ (gray; bottom-up) as a function of the applied potentials between −0.2 V and −0.8 V vs. RHE. A horizontal line is set at 50% FE to guide the eye. The bottom section of the figure shows the corresponding yield rate (μmol h^−1^ cm^−2^) for NO_2_^−^ (blue; triangle) and NH_3_ (gray; circle) as a function of the applied potentials. The corresponding NO_3_RR chronoamperometry plots and UV–Vis detection for NO_2_^−^ and NH_3_ concentrations are shown in Figs. S29–38. Error bars are determined from three replicate trials at −0.40 V vs. RHE. The GAP for the metal free N-C catalysts is shown in Fig. S39.

The electron energy loss spectroscopy (EELS) in Fig. 2a–c shows the EELS spectra of small sample regions containing single atoms of Fe, Rh, and La, respectively. The EELS point spectra show the co-existence of the N K-edge and the corresponding metal-edge (Fe-L_3,2_, Rh-M_3_, La-M_5_,_4_), supporting the bond formation between the singe metal atom and supporting nitrogen atoms (M-N_x_). X-ray absorption spectroscopy (XAS) was used to further investigate the chemical state and coordination environment of the catalysts, including X-ray absorption near-edge structure (XANES) and extended X-ray absorption fine structure (EXAFS). The XANES edge position and features (e.g., the intensity of the while line) of the Fe K-edge (7112 eV), Rh K-edge (23,219.9 eV) and La L_3_-edge (5890.6 eV) as compared to their corresponding reference foil and oxide compounds indicate oxidation states of *ca*. Fe^2+^, Rh^3+^, and La^3+^, in agreement with the Fe 2*p*, Rh 3*d* and La 3*d* X-ray photoelectron spectroscopy (XPS) analysis (Fig. S9). Interestingly, for Cr-N-C, evaluated to be one of the most selective M-N_x_ sites for the conversion of NO_3_^−^ to NH_3_ (Fig. 3d), the XANES analysis reveals a relatively high Cr oxidation sate, comprising a mixture Cr^6+^ (20–30%) and Cr^3+^ (70–80%). Analogously for other M-N-C catalysts, the chemical state of the metal center is assessed by their respective XANES and XPS spectra (Figs. S10–S16). Furthermore, the EELS valence state analysis for Fe-N-C and La-N-C, evaluating the intensity ratio and separation distance between the Fe L_3_/L_2_ and La M_5_/M_4_ edges further supports the oxidation state of Fe (between Fe^2+^ and Fe^3+^) and La (La^3+^) (Fig. S17). However, the Rh K-edge EELS signal is too weak to perform quantitative analysis. EXAFS provides structural information of the metal centers inner coordination spheres, confirming the presence of shorter bonds (M-C/M-N/M-O) rather than longer metallic M-M bonds. From comparison with complementary techniques (EELS and XPS) the formation of M-N bonds is supported. Fourier transformed (FT) EXAFS spectra for the Fe K-edge, Rh K-edge and La L_3_-edge, respectively are shown in Fig. 2d–f. In the Fe-N-C and Rh-N-C spectra, a single sharp peak was observed at a bond distance of ca. 1.5 Å (phase uncorrected) for both, characteristic of the Fe-N and Rh-N coordination in the first shell. While for La-N-C, an *f*-metal with a complex coordination environment (shown to sit out of plane during the modeling of the active site, Fig. 1b) a peak was observed at higher distances between 1 and 2.5 Å (phase uncorrected). A similar peak can also be observed in the FT-EXAFS of amorphous La_2_O_3_. Therefore, we assume that in our La-N-C sample a strongly disordered local environment around the single atom site, including the possible formation of small metal oxide-clusters. For the Ce-N-C catalyst, a more complex peak split feature was observed indicating the complex nature of *f*-metal M-N-C catalysts (Fig. S18). To deconvolute minor contributions to the EXAFS spectra at larger bond distances, wavelet transforms of the EXAFS oscillations, providing high resolution information in both k-space and R-space are shown in Fig. 2g–I. For Fe-N-C, Rh-N-C, and La-N-C, in addition to the high intensity Fe-N, Rh-N and La-N peak, a low intensity peak is observed at *ca*. 3.5 Å, 3.2 Å, and 3.7 Å (at low k-space values), respectively which we attribute to Fe-C, Rh-C, and La-C interactions in the second coordination shell. The lack of peaks in wavelet-transformed EXAFS spectra at higher values of wavenumber *k* indicates the absence of metal-metal bonds in the M-N-C catalysts, and, hence, absence of metallic or large oxide clusters. FT-EXAFS and WT-EXAFS for the M-N-C and corresponding metal oxide standards are given for Fe-, Rh-, and La-N-C in Fig. S19, while complete XANES, EXAFS, and FT-EXAFS spectra are provided for all M-N-Cs in Figs. S10–S12, with fitting parameters provided in Table S2 and fitting results plotted in R-space found in Fig. S20, respectively.

XPS was used to further examine the metal-nitrogen coordination and other nitrogen moieties present. The representative N 1s spectra for the Fe-, Rh-, and La-N-C catalysts in Fig. 2j–l, reveals the presence of metal-nitrogen moieties (M-N_x_). N 1*s* XPS spectra for the remaining M-N-C catalysts is shown in Figs. S21–24, all of which demonstrate the formation of M-N_x_ moieties. The deconvoluted high resolution N 1*s* XPS spectra showed a variation in the N-content and N-moiety percentage (e.g., pyridinic, pyrrolic, graphitic metal-N_x_), as shown in Tables S3–6.

In summary, 13 atomically dispersed M-N-C catalysts have been synthesized using the SSM. By combining AC-STEM, EELS, XAS and XPS, the atomically dispersed nature of each metal site has been comprehensively visualized and confirmed to be in a M-N_x_ coordination. Critically, the well-deciphered coordination environment of the metal center allows us to elucidate the intrinsic activity of the different M-N_x_ moieties towards the electrocatalytic transformation of reactive N-species.

### Electrochemical performance

The NO_3_RR is somewhat universal in that even metal-free nitrogen-doped-carbon (N-C) showed limited but observable activity towards the generation of NH_3_ and NO_2_^−^ under an isotopically labeled ^15^NO_3_^−^ feed in neutral electrolyte (pH = 6.3 ± 0.05) at −0.4 (V vs. RHE) (Fig. S25). Comparatively, with the addition of nitrogen-coordinated metal sites Cr-N-C as an example, the FE_NH3_ increased from *ca*. 10% (metal free N-C) to 91.2 ± 9.6%. While the introduction of Fe-N_x_ sites boosted the FE_NH3_ to 99.1 ± 2.6% (again at −0.4 V vs. RHE) and the corresponding Yield_NH3_ increased from 1.3 to 10.0 ± 1.3 μmol h^−1^ cm^−2^.

It should be noted that, for the NO_3_RR activity, as compared to measurements performed in neutral media, the current density and NH_3_ yield rate could be significantly enhanced under alkaline conditions, while maintaining a high FE_NH3_. Given Fe-N-C as an example, Fig. S26 shows that when using a 1 M KOH electrolyte (pH = 13.8 ± 0.17), the NH_3_ partial current density increased from 2 mA cm^−2^ (11.4 μmol h^−1^ cm^−2^) to 35 mA cm^−2^ (156.5 μmol h^−1^ cm^−2^) at −0.4 V and further to 175 mA cm^−2^ (750.4 μmol h^−1^ cm^−2^) at −0.6 V, both with a FE_NH3_ above 95%. Other work on alkaline NO_3_RR also found similar trends^18,33,38^. However, as a fundamental study, this work focused on neutral pH (pH 6.3 ± 0.05) to evaluate the intrinsic activity of the M-N_x_ sites toward the electrocatalytic NO_3_RR and NO_2_RR.

Regardless of the NO_3_RR pathways^39–41^, the surface intermediates *NO_2_ and *NOOH are inevitable before the reaction pathway diverges to its several possible products. The NO_2_^−^ molecule is also the first desorbable reaction intermediate, playing a key role in the NO_3_RR via a cascade pathway^24,42^. For the set of M-N-C catalysts in this work, the NO_2_RR displayed an earlier activation than the NO_3_RR by linear sweep voltammetry (LSV), as shown in Fig. 3a, indicating that the 6e^−^ transfer from NO_2_^−^/*NOOH to NH_3_, in the NO_3_RR was more facile than the initial 2e^−^ transfer from *NO_3_^−^ to *NO_2_^−^/*NOOH, which is the rate limiting step and thus the focus of the computational work in the following discussion. Representative LSV for the metal free N-C and a variety of 3*d* and 4*d* metals demonstrates the range of NO_3_RR (Fig. 3b), and NO_2_RR (Fig. 3c) activities, with Cu-N_x_ being the most active NO_3_RR site and Fe-N_x_ being the most active NO_2_RR site. As a unique nitrate-to-nitrite electrocatalyst^24^, Mo-N-C was even more inert than the metal free N-C. A full analysis of the reaction onset potentials (Fig. S27) clearly visualizes the range of onset potentials for the diverse M-N_x_ sites.

To investigate the NO_3_RR activity and selectivity towards NH_3_ and NO_2_^−^, a series of 2-h potential holds were performed at potentials between −0.2 V and −0.8 V. A gap analysis plot (GAP) was constructed in Fig. 3d to visualize the nitrogen conversion landscape. Collectively, as the cathodic potential decreased, the yield rate of NH_3_ and NO_2_^−^ increased for all catalysts, but the selectivity varied. Specifically, both Fe-N-C and Cr-N-C showed the highest NH_3_ selectivity (FE_NH3_ 92–100%) at −0.4 V and −0.6 V. In contrast, Mo-N-C showed comparable, consistent, and somewhat potential-independent selectivity for NO_2_^−^ (40–50% FE_NO2-_) and NH_3_ (50–55% FE_NH3_), as expected given that Mo-N_x_ sites were shown in our previous work, as an active nitrate-to-nitrite converter via dissociative adsorption (chemical process)^24^. Similarly, the Mn-, Pd-, W-, La-, and Ce-N-C showed moderate NO_2_^−^ and NH_3_ selectivity at high overpotentials (~70% FE_NH3_ vs. ~30% FE_NO2-_) and the FE for nitrogen conversion (FE_NH3_ + FE_NO2-_) was maintained at 100%. Other early transition metals (Co-, Ni- and Cu-N-C) showed irregular potential dependence on selectivity, wherein Co-N-C showed a volcano trend for FE_NH3_ but consistent FE_NO2-_ and the nitrogen conversion was far below 100% FE through the entire potential range. For an easier comparison, the NO_3_RR electrolysis results (FE_NH3/NO2-_ and Yield_NH3/NO2-_) have been separated based on the applied potential in Fig. S40, where its clearly observed that Cr-N-C was leading the other catalysts between −0.2 V and −0.6 V, being exceptional at −0.2 V (9.33 μmol h^−1^ cm^−2^ and 94% FE_NH3_). However, Ni-N-C and Cu-N-C showed a strong potential dependence on the Yield_NH3_, where Cu-N-C rivals Cr-N-C at −0.8 V (61 vs. 59 μmol h^−1^ cm^−2^). Particularly, Cu-N-C maintained 100% FE for NH_3_ + NO_2_^−^ (FE_NH3_ = 72%) at −0.8 V, indicating that no current is wasted on the parasitic HER. Two distinct exceptions were Ru-N-C and Rh-N-C, albeit their early activation indicated by LSV (Fig. 3), both of which showed inferior NH_3_ and NO_2_^−^ activity (Fig. 3d), displaying unique and significant gaps between the NH_3_ and NO_2_^−^ FE bars. This gap in FE is likely due to insoluble, undetected gas phase products (Fig. S41), likely from the HER, as the isolated active sites present in atomically dispersed catalysts are unfavorable for the coupling of N-N bonds for N_2_.

It was recently observed for the NO_3_RR and previously reported for the CO_2_RR that under a cathodic potential, atomically dispersed Cu-N_x_ sites can reduce to form metallic clusters and even nanoparticles at more cathodic potentials^43–45^. Interestingly, additional investigations employing operando XAS over other atomically dispersed transition metal sites (M = Mn, Fe, Co, Ni and Pd) revealed a stable atomically dispersed M-N_x_ site, even under highly reductive potentials^46–49^. Further studies employing post-mortem STEM observed atomically dispersed sites after reductive potentials in the CO_2_ and N_2_ reduction reactions, over Ru-, Rh-, Ce-N-C, and other rare earth metals^50–54^. To investigate possible morphological changes to the atomically dispersed nature of the catalysts in this work after the NO_3_RR electrolysis, the representative Fe-, Rh-, and La-N-C catalysts were imaged. Post-mortem STEM images in Figs. S42–S44 confirm atomically dispersed nature of the Fe-, Rh- and La-N_x_ sites after the series of NO_3_RR electrolysis from −0.2 to −0.8 V for 2 h each. It is important to note that post-mortem STEM does not reveal possible in-situ restructuring under electrolysis conditions. However, as reported in a recent study employing Cu-N-C for the NO_3_RR, as morphological reconstructions were occurring as a result of the cathodic potential, a significant increase in the current is readily observed over time in the electrochemical response (chronoamperometry curves) as the Cu-N_x_ sites transformed into metallic Cu clusters/particles^43^. Similar changes in the electrochemical response would be expected in the current study during both the NO_3_RR and NO_2_RR if significant morphological reconstruction was occurring, however, this was not observed. This may suggest, however not confirm without operando XAS, that even for Cu-N-C (which has been shown to be more susceptible to morphological changes under reductive potentials than other M-N-C catalysts), significant morphological reconstruction of the Cu-N_x_ sites may not be occurring. Perhaps because of variations in the stability of the Cu-N_x_ sites, originating from the different M-N-C synthesis approaches and precursor selections. This behavior is observed in the literature for Cu-N-C, by comparing operando studies under reductive potentials, for which some studies report active site reconstruction at −0.2 V vs. RHE^43^ and others report no changes up to −0.6 V vs. RHE^44^. Additionally, at the mild cathodic potential in this work of −0.2 V, at which the correlations (discussed in the following section) are most accurate, significant morphological reconstruction may not be expected and was not observed over time in the electrochemical NO_3_RR and NO_2_RR performances, however, direct spectroscopic evidence would be required to confirm this hypothesis. Although no M-N_x_ restructuring was observed through post-mortem STEM (Fe-, Rh-, and La-N-C) or suggested by the electrochemical response, this cannot be totally ruled out and will require future operando XAS studies for a robust evaluation.

**Fig. 4:**
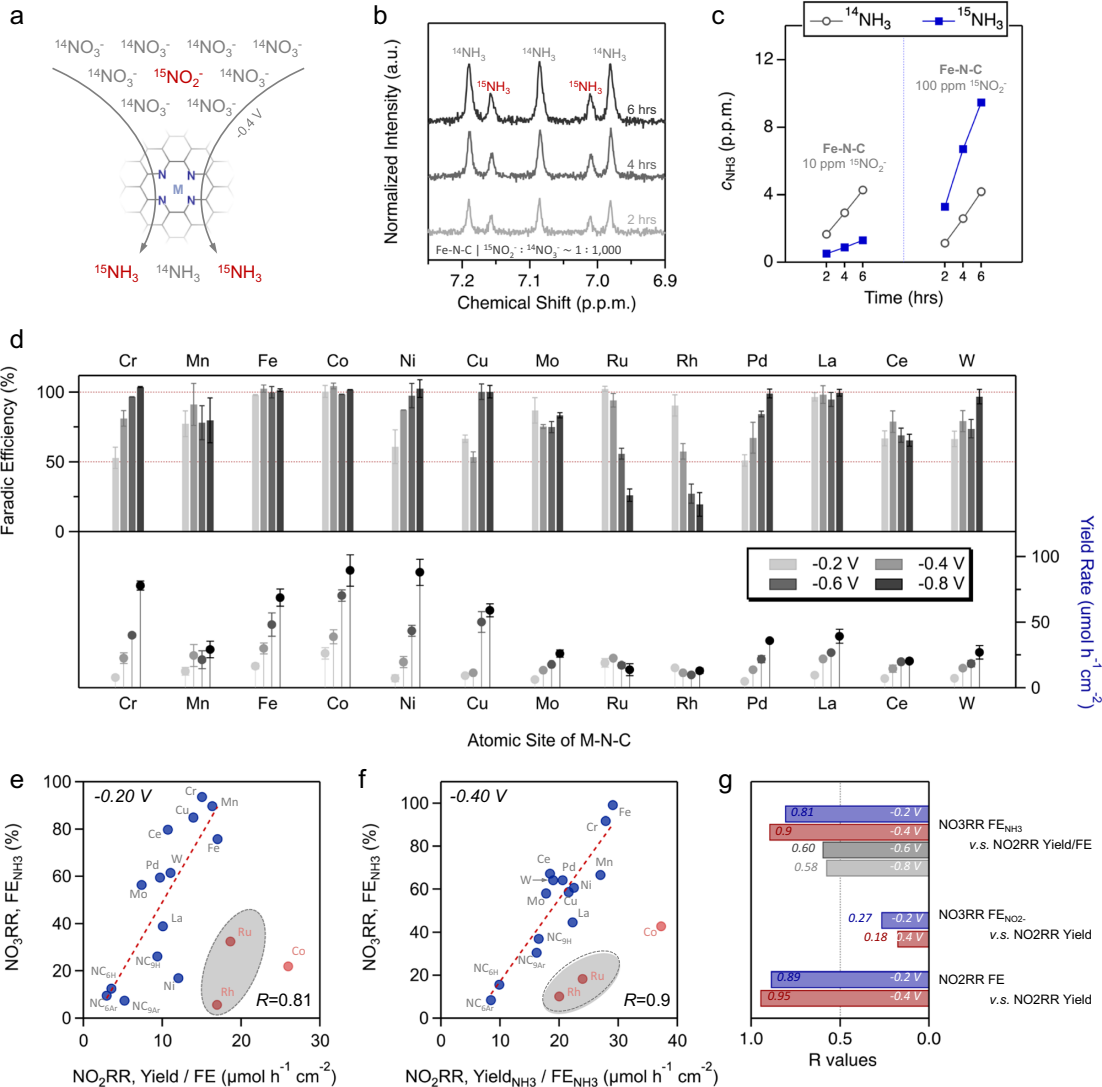
Mechanistic analysis of the NO_3_RR via a NO_2_^−^ intermediate, NO_2_RR performance and experimental correlations between lining NO_3_RR selectivity and NO_2_RR performance. **a**–**c** Isotopic analysis of competing NO_3_RR and NO_2_RR reactions at −0.4 V. **a** Schematic for NO_3_RR electrolysis in which small amounts of ^15^NO_2_^−^ are doped in 0.16 M ^14^NO_3_^−^. **b** NMR spectra for the electrolysis with 10 ppm of ^15^NO_2_^−^ doped in 0.16 M ^14^NO_3_^−^ (10,000 ppm), sampled at 2, 4, and 6 h. **c** Time course analysis of ^14^NH_3_ and ^15^NH_3_ concentration at 2, 4, and 6 h for the NO_3_RR with 10 ppm ^15^NO_2_^−^ (left) and 100 ppm ^15^NO_2_^−^ (right) in 0.16 M ^14^NO_3_^−^ (10,000 ppm). **d** Electrochemical NO_2_RR for M-N-C catalysts in 0.05 M PBS + 0.01 M KNO_2_ for 0.5 h. Faradaic efficiency for NH_3_ as a function of the applied potential between −0.2 V and −0.8 V (top). Bottom section shows the corresponding NH_3_ yield rate. The chronoamperometry plots and UV-Vis detection curves for NH_3_ are shown in Figs. S50–56. Note, FE’s over 100% arise from electrolyte dilution to bring the concentration of NH_3_ into the UV-vis calibrated range. Error bars are determined from three replicate trials. Correlations between the NO_3_RR Faradaic efficiency for NH_3_ in Fig. 3d, where *R* is the correlation coefficient (y-axis) and the dividend of the NH_3_ yield rate and Faradaic efficiency of the NO_2_RR in Fig. 4d at (**e**) −0.20 V and (**f**) −0.40 V. The NO_2_RR, Yield_NH3_ /FE_NH3_ is represented as the NO_2_RR total current (NO_2_RR, j_total_). The correlation between the NO_2_RR, Yield_NH3_ and NO_3_RR, FE_NH3_ is shown in Fig. S57. Ru and Rh outliers (gray shades) were excluded from the linear fit due to the dominant NO_3_RR gaseous products as shown in Fig. S41 as well as Co-N-C. **g** Summary of the correlation coefficients, *R*, for varying experimental NO_3_RR-NO_2_RR correlations. Correlations not shown in Fig. 4e, f, are shown graphically in Figs. S58–60.

For the role of potential nitrite (as a by-product or intermediate), previous reports usually considered the NO_3_RR as a direct 8e^−^ transfer pathway with certain irreversible NO_2_^−^ desorption or leaching^32,33,55^. Here, doping of isotopic ^15^NO_2_^−^ in the NO_3_RR, schematically shown in Fig. 4a, revealed that trace amounts (e.g., 1 ppm) of ^15^NO_2_^−^ could be easily reduced to ^15^NH_3_ even under a concentrated ^14^NO_3_^−^ environment (10,000 ppm), which applied to both M-N_x_ sites and metal-free N-C sites (Figs. S45–46). Figure 4b shows the NMR spectra for a time course electrolysis over Fe-N-C, wherein even at a concentration ratio of 1,000:1 (^14^NO_3_^−^: ^15^NO_2_^−^), the ^15^NO_2_^−^ competed for an active site, yielding comparable ^15^NH_3_. NMR spectra for the other M-N-C catalysts is shown in Fig. S47, where a similar trend is observed for most metals with the no exception of Co- and Ni-N-C, where significantly more isotopic ^15^NH_3_ is generated than standard ^14^NH_3_, in support of Fig. 3d, suggesting their poor activity towards NO_3_^−^ but high activity towards NO_2_^−^, which will be addressed in the following section. As the concentration ratio is reduced to 100:1 (^14^NO_3_^−^: ^15^NO_2_^−^), the ^15^NO_2_^−^ outcompeted ^14^NO_3_^−^, yielding significantly more ^15^NH_3_ than ^14^NH_3_ over the 6-h electrolysis (Fig. 4c). This indicates that for the nitrogenous products in the NO_3_RR, the potential participation of bulk NO_2_^−^ or locally channeled NO_2_^−^, significantly complicates the mechanism of NH_3_ production (e.g., 2e^−^ + 6e^−^ vs. 8e^−^)^24,56^. Therefore, unambiguously identifying whether the underlying reaction mechanism is a direct 8e^−^ pathway, a (rapid) 2e^−^ + 6e^−^ pathway with a bulk/channeled NO_2_^−^ intermediate, or a combination thereof, is of great significance for the design and optimization of NO_3_RR systems and even more complex nitrate-involving reactions^57,58^.

Figure 4d (and shown individually by applied potential in Figs. S48–49) shows the NO_2_RR electrolysis for all M-N-C catalyst under a 0.01 M NO_2_^−^ feed, a concentration mimicking the bulk NO_2_^−^ concentrations in the NO_3_RR electrolysis (Fig. 3d). Obviously, both the NH_3_ selectivity and activity of the NO_2_RR were significantly higher than that of the NO_3_RR (Fig. 3d) for all catalysts. Specifically, Fe-, Co-, and La-N-C showed 100% FE_NH3_ over the whole potential range and Cr-, Ni-, Cu-, Pd-, and W-N-C showed increasing FE_NH3_ as the cathodic potentials decreased, reaching 100% at −0.8 V. Again, Mo-N-C showed a unique and potential-independent FE_NH3_ around 75%. Similar to the NO_3_RR, the NO_2_RR for Ru-N-C and Rh-N-C showed a distinct decreasing trend on the FE_NH3_ and consistent Yield_NH3_ over the entire potential range, likely being outcompeted by the HER as the cathodic potential decreased.

To examine the relationship between the NO_2_RR and NO_3_RR, Fig. 4e, f correlates the activity of NO_2_RR (Yield_NH3_/FE_NH3_) with the selectivity of NO_3_RR (FE_NH3_), revealing a linear relationship at −0.20 V and −0.40 V. This linear relationship suggests a major contribution from the bulk or locally channeled NO_2_^−^ towards NH_3_ (2e^−^ + 6e^−^ pathway). Meanwhile, the correlation between the FE_NO2-_ in the NO_3_RR and the Yield_NH3_ in the NO_2_RR, has a poor linear fit (Fig. S59), highlighting the complex reaction mechanism. In contrast, the poor correlations between the NO_3_RR FE_NO2-_ and NO_2_RR Yield_NH3_ (Fig. 4g), confirmed that in the NO_3_RR the bulk NO_2_^−^ species were in a complex production-consumption process rather than an irreversibly desorbed final by-product.

### Computational descriptors for the NO_3_RR and NO_2_RR

**Fig. 5:**
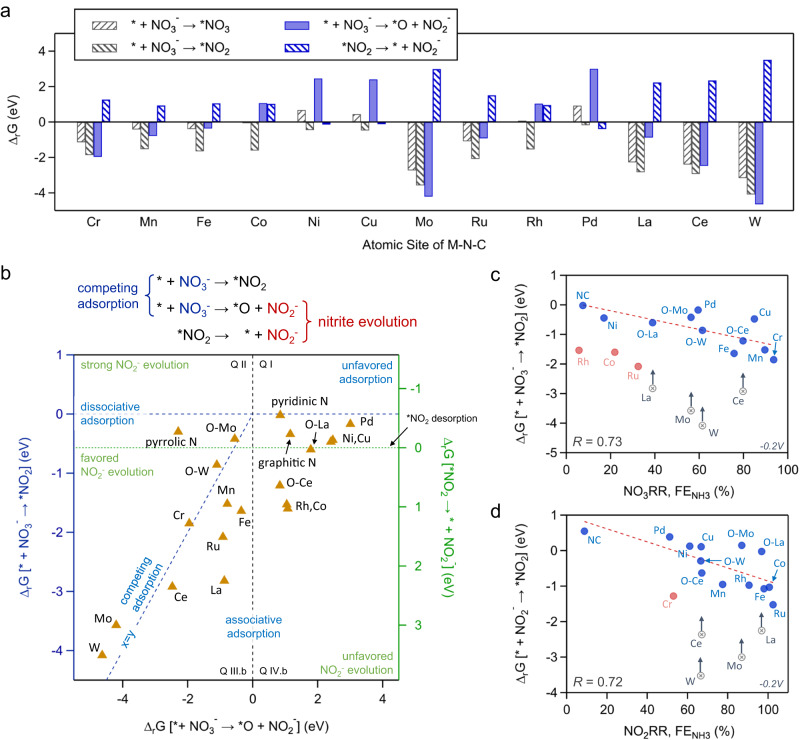
Computational NO_3_RR descriptors as calculated by using DFT with optB86b-vdW functional. **a** Gibbs free energies (∆_r_G) of the reaction for the first two electron transfer steps in the NO_3_RR. Processes that generate NO_2_^−^ in the bulk electrolyte are shown in blue. Note solid bars indicate no electron transfer, uphill gradient stripes indicate a reductive e^−^ transfer and downhill gradient stripes indicate an oxidative/reverse electron transfer. **b** Quadrant plot of the ∆_r_G for associative adsorption $[{\,\!}^{*}+{{{{{{\rm{NO}}}}}}}_{3}^{-}\to {\,\!}^{*}{{{{{{\rm{NO}}}}}}}_{2}]$ (Y_1_-axis), dissociative adsorption $[{\,\!}^{*}+{{{{{{\rm{NO}}}}}}}_{3}^{-}\to {\,\!}^{*}{{{{{{\rm{O}}}}}}+{{{{{\rm{NO}}}}}}}_{2}^{-}]$ (X-axis) and *NO_2_ desorption $[{\,\!}^{*}{{{{{{\rm{NO}}}}}}}_{2}\to {\,\!}^{*}+{{{{{{\rm{NO}}}}}}}_{2}^{-}]$ (Y_2_-axis), forming sectors where certain reaction pathways are thermodynamically favored. The main quadrants were determined by the X-axis and Y_1_-axis. Quadrants III and IV were further divided by the Y_2_-axis into III.a, III.b, IV.a and IV.b sub^−^sections. The three reaction coordinates determined two types of NO_3_^−^ adsorptions and two types of NO_2_^−^ evolutions as shown by the diagram above the figure. For simplified plots correlating two descriptors at a time, see Fig. S63. **c** Correlation between DFT-derived ${\Delta }_{{{{{{\rm{r}}}}}}}{{{{{\rm{G}}}}}}[{\,\!}^{*}+{{{{{{\rm{NO}}}}}}}_{3}^{-}\to {\,\!}^{*}{{{{{{\rm{NO}}}}}}}_{2}]$ and experiment-derived NO_3_RR FE_NH3_ at −0.2 V vs. RHE. **d** Correlation between DFT-derived ${\Delta }_{{{{{{\rm{r}}}}}}}{{{{{\rm{G}}}}}}[{\,\!}^{*}+{{{{{{\rm{NO}}}}}}}_{2}^{-}\to {\,\!}^{*}{{{{{{\rm{NO}}}}}}}_{2}]$ and experiment-derived NO_2_RR FE_NH3_ at −0.2 V vs. RHE. Oxygenated active sites (O-M) were included for the oxyphilic elements (Mo, La, Ce and W), where *R* is reported as the absolute value of the correlation coefficient.

As discussed above, the first 2e^−^ transfer is the rate limiting step in the NO_3_RR, therefore DFT was used to study the energetics of key intermediates in the conversion of NO_3_^−^-to-NO_2_^−^ on various catalytic active sites (Fig. S61). This included the *NO_3_ surface intermediate formed by oxidative associative adsorption of NO_3_^−^, the *O surface intermediate formed by neutral dissociative adsorption of NO_3_^−^^24^, and the *NO_2_ surface species formed in the reductive adsorption of NO_3_^−^, these result in four thermodynamic reaction descriptors, as shown in Fig. 5a.

Figure 5a shows that the Mo-N_4_, La-N_4_, Ce-N_4_, and W-N_4_ sites could strongly adsorb NO_3_^−^ (**∆**_**r**_**G** ~ −4eV) in a dissociative manner, forming an *O surface species and a free NO_2_^−^ molecule. For these oxyphilic metals, upon exposure to NO_3_^−^ molecules, the single-atom sites are oxygenated with a fifth ligand and the new active site (O-M-N_4_) can still coordinate a NO_3_^−^/NO_2_^−^ molecule for further reduction^24^. Figure S62 shows that the O-M-N_4_ sites had a weaker interaction with NO_3_^−^ but could stabilize the *NO_3_/*NO_2_ surface intermediates. For the Cr-N_4_, Mn-N_4_, Fe-N_4_^,^ and Ru-N_4_ sites, associative and dissociative adsorption of NO_3_^−^ is likely a competitive process that also depends on the cell potential. Namely, while the associative adsorption of NO_3_^−^ is potential dependent, dissociative adsorption is not as no electrons are involved in this process. Co-N_4_, Ni-N_4_, Cu-N_4_, Pd-N_4_, and La-N_4_ sites adsorb NO_3_^−^ associatively and produce NH_3_ via a *NO_2_ intermediate. Additionally, our DFT descriptors suggest limited NO_3_RR activity over Rh-N_4_ sites, being only favorable via the reductive adsorption of NO_3_^−^, while in other computational based works, Rh-N_4_ sites suffer some competition between *H and *NO_3_, in agreement with the poor NO_3_RR activity observed in our experimental observations^41,59^.

The quadrant plot in Fig. 5b graphically shows the correlations between the DFT-derived descriptors, mapping out the competitive NO_3_^−^ adsorption and sources for NO_2_^−^ evolution. Specifically, the far ends of Quadrant I, Quadrant II and Quadrant IV.b represent unfavored NO_3_^−^ adsorption, exclusive NO_2_^−^ evolution and unfavored NO_2_^−^ evolution, respectively. These three blank sectors explain the above-mentioned universal NO_3_RR activity for all M-N-C and metal free N-C catalysts (Fig. S64), as well as the variant but non-dominant FE_NO2-_ in the NO_3_RR. The diagonal of Quadrant III (*x* = *y*) indicates competitive associative and dissociative adsorption of NO_3_^−^. The largest NO_2_^−^ producing catalyst, Mo-N-C (O-Mo-N_4_ and Mo-N_4_ sites), is located at the diagonal, indicating comparable associative and dissociative adsorption. Several metal centers (e.g., Mn, Fe, Ru, La, and Ce) fall below the diagonal in Quadrant III.b and upper Quadrant IV.b, suggesting a favored associative NO_3_^−^ adsorption for high NH_3_ selectivity in NO_3_RR electrolysis (Fig. 3d). Quadrant IV.b (Co, Rh, O-Ce and O-La) marks the region where a solely direct 8e^−^ pathway to NH_3_ is feasible, with formation of the *NO_2_ intermediate being favored, while both descriptors for the generation/desorption of NO_2_^−^ are unfavored. Quadrant IV.a (Ni, Cu and Pd, pyridinic-N and graphitic-N) shows a weak stabilization of the *NO_2_ intermediate, while simultaneously showing activity for the desorption of *NO_2_ for NO_2_^−^ evolution. It should be noted that Mo-N-C is the only metal center favoring NO_2_^−^ evolution through both the dissociative NO_3_^−^ adsorption and *NO_2_ desorption paths, explaining the unique selectivity of Mo-N-C in the NO_3_RR (Fig. 3d). The DFT calculated Gibbs free energy changes for these descriptors are given in Table S7.

To evaluate the practical relevance of these computational descriptors, a set of correlations were developed between the DFT-derived free energies (∆_r_G) in Fig. 5a and the electrocatalytic performance in Figs. 3d and 4d. Specifically, Fig. 5c shows the correlation between the adsorption energy of NO_3_^−^ (${\Delta }_{{{{{{\rm{r}}}}}}}{{{{{\rm{G}}}}}}[{\,\!}^{*}+{{{{{{\rm{NO}}}}}}}_{3}^{-}\to {}^{*}{{{{{{\rm{NO}}}}}}}_{2}]$) and the NO_3_RR NH_3_ selectivity (FE_NH3_), wherein a linear correlation (*R* = 0.73) is observed at −0.2 V. This result highlights the importance of a stable *NO_2_ intermediate for high NH_3_ selectivity in both the 8e^−^ pathway or the 2e^−^ + 6e^−^ pathway (which requires the re-adsorption of NO_2_^−^). Additionally, the involvement of the oxo-form for the highly oxyphilic elements (O-Mo, O-W, O-La, O-Ce) shifts them back onto the trend line as compared to their bare M-N_4_ counterparts. These results suggest the distinct active sites/reaction mechanism of early transition 4*d-*, 5*d-* and *f-*metal-based M-N-C catalysts in neutral environments, which could be attributed to the oxophilicity and large coordination number of these metals that allow simultaneous coordination of multiple intermediates^60^. Similarly, Fig. 5d shows a comparable correlation (*R* = 0.72) between the adsorption energy of NO_2_^−^ (${\Delta }_{{{{{{\rm{r}}}}}}}{{{{{\rm{G}}}}}}[{\,\!}^{*}+{{{{{{\rm{NO}}}}}}}_{2}^{-}\to {}^{*}{{{{{{\rm{NO}}}}}}}_{2}]$) and the NO_2_RR ammonia selectivity (FE_NH3_), indicating that the stabilization of the *NO_2_ intermediate also plays a key role in the NO_2_RR as well as the downstream 6e^−^ transfer in the NO_3_RR. It is important to note potential limitations of these computational-experimental correlations. From our observations, metal centers over which H* adsorption is a competing factor leading to the HER dominating (Ru- and Rh-N-C, Fig. 3) the NO_3_RR are not well predicted by these correlations. Similarly, metal centers which demonstrate significant NO_2_^−^ activation, with poor NO_3_^−^ activation (observed over Co-N-C in the isotopic NO_2_^−^ doping experiments, Fig. S47) are also not well predicted by these correlations.

However, the NO_3_RR FE_NO2-_ showed minimum correlations with either ${\Delta }_{{{{{{\rm{r}}}}}}}{{{{{\rm{G}}}}}}[{\,\!}^{*}{{{{{{\rm{NO}}}}}}}_{2}\to {\,\!}^{*}+{{{{{{\rm{NO}}}}}}}_{2}^{-}]$ or ${\Delta }_{{{{{{\rm{r}}}}}}}{{{{{\rm{G}}}}}}[{\,\!}^{*}+{{{{{{\rm{NO}}}}}}}_{3}^{-}\to {\,\!}^{*}{{{{{{\rm{O}}}}}}+{{{{{\rm{NO}}}}}}}_{2}^{-}]$, as shown in Fig. S65. This agrees well with the above-mentioned poor correlation between the NO_3_RR FE_NO2-_ and NO_2_RR Yield_NH3_ (Figs. 4g and S59), wherein the bulk NO_2_^−^ species in the NO_3_RR were in an active but and complex production-consumption process, making it difficult to deconvolute the NO_2_^−^ production and consumption rates from its net yield.

## Discussion

In summary, a rich set of atomically dispersed 3*d*-, 4*d*-, 5*d*-, and *f*-block M-N-C catalysts with a well-established M-N_x_ coordination was synthesized. The gap analysis plot revealed diverse NO_3_RR performance, wherein Cr-N-C and Fe-N-C were the most NH_3_ selective catalysts, achieving near 100% FE_NH3_, while Mn-, Cu-, and Mo-N-C were highly selective for NO_2_^−^. For the NO_2_RR, several elements including Cr-, Fe-, Co-, Ni-, Cu- and La-N-C achieved a FE_NH3_ of 100% at high overpotentials, with Fe and Co-N-C showing 100% FE_NH3_ over the entire potential range. Isotopically doped ^15^NO_2_^−^ in concentrated ^14^NO_3_^−^, demonstrated the ease at which minute concentrations of NO_2_^−^ in the bulk electrolyte can preferentially reduce to NH_3_, convoluting the possibility of a direct 8e^−^ pathway or a 2e^−^ + 6e^−^ cascade pathway with NO_2_^−^ as transient intermediate for the NO_3_RR. The correlation between experimental NO_3_RR ammonia selectivity and experimental NO_2_RR activity suggested a universal contribution of the NO_2_^−^ intermediate in NO_3_RR (2e^−^ + 6e^−^). The DFT-derived thermodynamic descriptors can theoretically explain the electrocatalytic selectivity for each metal center. Furthermore, these computational descriptors showed good correlations with the experimental performances, such that a simple computationally evaluated descriptor can be utilized to estimate the activity of M-N-C catalysts for the NO_3_RR and NO_2_RR. While these computational-experimental activity descriptors provide correlations for the NO_3_RR and NO_2_RR activity, these correlations are limited when the H^+^ adsorption is a competing factor leading to the HER over NO_3_RR (Ru- and Rh-N-C) and when there is poor NO_3_^−^ activation in contrast with significant NO_2_^−^ activation (Co-N-C). Importantly, these computational-experimental activity descriptors are based on the M-N_x_ active site and have strong predictive NO_3_/NO_2_RR ability at low potentials, where the intrinsic nature of the M-N_x_ site is observed. However, the effectiveness of these activity descriptors decreases as the overpotential increases, forcibly driving the reaction and possibly inducing structural changes to the M-N_x_ sites, convoluting the intrinsic activity of the atomically dispersed metal center. This work deciphered the unique fundamentals of the NO_3_RR over atomically dispersed M-N-C catalysts to create a set of experimentally driven computational descriptors for the NO_3_/NO_2_RR activity, paving the road for the design of tandem NO_3_RR systems and nitrate-containing systems composed of either multi-metallic M-N-C catalysts or extended catalytic surfaces supported by synergistic M-N-C supports.

## Methods

### Materials

Nicarbazin (Sigma-Aldrich), CAB-O-SIL® LM-150 fumed silica (Cabot), Aerosil® OX-50 (Evonik), iron(III) nitrate nonahydrate (Sigma-Aldrich), chromium(III) acetylacetonate (Sigma-Aldrich), manganese(II) nitrate tetrahydrate (Sigma-Aldrich), cobalt(II) nitrate hexahydrate (Sigma-Aldrich), nickel(II) nitrate hexahydrate (Sigma-Aldrich), copper(II) nitrate hemi pentahydrate (Sigma-Aldrich), ammonium molybdate tetrahydrate (Sigma-Aldrich), ruthenium(III) nitrosylnitrate (Alfa Aesar), rhodium(III) nitrate hydrate (Sigma-Aldrich), palladium(II) nitrate dihydrate (Sigma-Aldrich), lanthanum(III) nitrate hexahydrate (Alfa Aesar), cerium(III) nitrate hexahydrate (Fisher Scientific) and ammonium paratungstate (Sigma-Aldrich), potassium nitrate (Sigma-Aldrich), potassium nitrite (Sigma-Aldrich), isotopic potassium nitrate (^15^N, 99% - Cambridge Isotope Laboratories), isotopic sodium nitrite (^15^N, 98%^+^ - Cambridge Isotope Laboratories).

### Synthesis of atomically dispersed M-N-C catalysts

All catalysts were synthesized following the well-established sacrificial support method, as detailed below, with the metal precursor loading, pyrolysis temperature, pyrolysis atmosphere and etching environment being tuned to maintain atomically dispersed metal sites between the different metals (Supplementary note 1).

#### Synthesis of Mn, Fe, Co, Ni, Cu, Mo and W-N-C catalysts

First a slurry of a carbon-nitrogen containing precursor, Nicarbazin (6.25 g), the silica sacrificial support, LM-150 (Cabot, 1.25 g), OX-50 (Evonik, 1.25 g) and Stöber spheres (made in house, 0.5 g) and the corresponding metal salt precursor (Mn = 0.266 g, Fe = 0.60 g, Co = 0.272 g, Ni = 0.271 g, Cu = 0.345 g, Mo = 0.262 g and W = 0.095 g), in 50 mL of MilliQ water was created. Next, the slurry was sonicated for 30 min before being dried overnight at 45 °C, under constant stirring. The mixture was then further dried in an oven at 45 °C for 24 h. The resulting powder was then ball milled at 45 Hz for 1 h. The catalyst powder is then pyrolyzed at 975 °C (with a ramp rate of 15 °C min^−1^) under a reductive H_2_/Ar (7%/93%) atmosphere for 45 min. The pyrolyzed powder is then ball milled a second time at 45 Hz for 1 h. The silicate template is then etched in a hydrofluoric acid (15 M) solution for 96 h. The catalyst is then recovered by filtration and washed to neutral pH, followed by drying at 60 °C overnight. The catalyst undergoes a second pyrolysis in a reductive NH_3_/N_2_ (10%/90%) atmosphere at 950 °C for 30 min (with a ramp rate of 20 °C min^−1^). The catalyst is then ball milled a final time at 45 Hz for 1 h.

#### Synthesis of Cr-N-C catalyst

The synthesis is identical to the previous procedure, with only the pyrolysis temperatures being reduced to 650 °C in both the first and second pyrolysis, to maintain an atomic dispersion of the Cr metal. The metal salt precursor loading of Cr = 0.519 g.

#### Synthesis of Ru-N-C catalyst

The synthesis is identical to the previous procedure, with the pyrolysis temperatures being reduced to 650 °C and an inert argon pyrolysis atmosphere, to maintain an atomic dispersion of the Ru metal. The metal salt precursor loading of Ru = 0.235 g.

#### Synthesis of Rh and Pd-N-C catalysts

The synthesis is identical to the previous procedure, with the high pyrolysis temperatures of 975 °C and 950 °C for the first and second pyrolysis, respectively, however the pyrolysis is performed in an inert argon atmosphere, to maintain an atomic dispersion of the Rh and Pd metals. The metal salt precursor loading of Rh = 0.215 g and for Pd = 0.198 g.

#### Synthesis of the La and Ce-N-C catalysts

The synthesis is identical to the previous procedure, with the pyrolysis temperatures being reduced to 650 °C under an inert argon pyrolysis atmosphere. Furthermore, the etching of the silica template was performed in an alkaline 4 M NaOH environment at 80 °C for 96 h. If etched using a hydrofluoric acid environment, the La and Ce readily form LaF_3_ and CeF_3_ nanoparticles. The metal salt precursor loading of La = 0.060 g and Ce = 0.061 g.

### Physical characterization

The physical structure and atomically dispersed nature of the catalysts were analyzed by aberration-corrected scanning transmission electron microscopy (AC-STEM) and EDS using a JEOL ARM300CF at an accelerating voltage of 300 kV. The coordination environment and valence state of the atomically dispersed sites were examined through atomic resolution electron energy loss spectroscopy (EELS) on a Nion UltraSTEM200 microscope equipped with a cold FEG, a C3/C5 aberration correction and a high-energy resolution monochromated EELS system (HERMES). To mitigate the beam damage on the coordination between single metal atoms and nitrogen-carbon support, the EELS was collected at a low voltage of 60 kV. During acquisition, the energy dispersion was set as 0.16 ~ 0.3 eV/channel with an exposure time of 2–4 s nm^−2^. The background in each spectrum was removed by a power-law function and the de-noising of the spectra was performed by the multivariate weighted principal component analysis routine in the Digital Micrograph software.

The hierarchical pore structure of the catalyst was examined by scanning electron microscopy (SEM) on an FEI Magellan 400 XHR SEM.

The electronic structure and local bonding environment of the metal sites were analyzed by ex-situ X-ray Absorption Spectroscopy (XAS) collected on several beamlines. Rh K-edge was measured on the SAMBA beamline at SOLEIL synchrotron radiation facility, Paris, France. The sample was measured in fluorescence mode and references in transmission mode using a Si (220) monochromator for the energy selection. Ionization chambers to measure the X-ray intensity before and after the sample were filled with a mixture of Ar/N_2_ (*I*_0_) or pure Ar (*I*_1_/*I*_2_).

The La L_3_-edge was measured on the XAFS beamline at ELETTRA synchrotron radiation facility, Triest, Italy. The sample was measured in transmission mode using a Si (111) 20% detuned monochromator for the energy selection. Simultaneously, a vanadium reference foil was measured for the energy calibration. Ionization chambers to measure the X-ray intensity before and after the sample were filled with a mixture of N_2_/He (*I*_0,_ *I*_1_, *I*_2_).

The Fe K-edge was measured on the lab-based easyXAFS300 at the Fritz Haber Institute, Berlin, Germany (measured in transmission mode). Using a Ge (620) crystal for the energy selection and a Si drift detector (AXAS-M assembly from KETEK GmbH). A W-ProtoXRD X-ray tube was used.

The Ce L_3_-edge, Cr, Mn, Co, Ni and Cu K-edge were measured on the KMC-3 beamline at the Bessy synchrotron radiation facility, Berlin, Germany. Cr-N-C in transmission mode, Cu-, Co-, Ni-, Mn- and Ce-N-C in fluorescence mode using a Si (111) monochromator for the energy selection. The ionization chamber for the measurement of the X-ray intensity before the sample was filled with 100 mbar air (*I*_0_). Either a 13 element Si drift detector was used (fluorescence) or PIPS detector (transmission). Note that the energy resolution of the XAS spectra measured at the KMC-3 beamline is compromised due to technical issues involving the optics/mirrors at the KMC-3 beamline. However, the samples and corresponding reference foils and compounds were measured for energy calibration and comparison of features. Note Mo-N-C and Pd-N-C were measured on the 10-BM beamline of the Advanced Photon Source at Argonne National Laboratory.

The ATHENA software was used for data alignment and XAS spectra extraction^61^. A set of in-house built Wolfram Mathematica scripts were used for the XANES analysis. Fitting of the EXAFS spectra was done using FEFFIT scripts^61^. The photoelectron amplitudes and phases were calculated by the FEFF8 code^62^. The EXAFS R-space fitting parameters and results are given in Table S8. If the M-N-C sample and corresponding reference material were not measured at the same beamline (Cr-, La-, and Ce-N-C) the *S*_0_^2^ factor multiplied by the coordination number is reported.

The metal valence state and quantification of nitrogen-moieties of the M-N-C catalysts were analyzed by XPS. The XPS was performed on a Kratos AXIS Supra spectrometer with a monochromatic Al Kα source (with an emission current of 15 mA and X-ray power of 225 W, while for the high-resolution spectra the emission current and power are 20 mA and 300 W, respectively). No charge neutralization was employed as all these samples are highly conductive, carbon-based catalysts. Survey spectra were obtained at a pass energy of 160 eV from 1400 eV to 5 eV at a step size of 1 eV. The High-resolution C 1s, N 1s and O 1s spectra were obtained at a pass energy of 20 eV with a 0.1 eV step size. The metal spectra were obtained at a pass energy of 40 eV with a 0.1 eV step size. A C 1s spectra was also obtained at a pass energy of 40 eV for calibration. CasaXPS software was used to analyze and fit all spectra and all spectra were calibrated based on the sp^2^ carbon (284 eV). A Linear background was employed for the C 1s and N 1s spectra, while a Shirley background was used for the O 1s and metal spectra. The sp^2^ carbon in the C 1s spectra was fit with a 80% Gaussian/20% Lorentzian function and all other spectra were fit using a 70% Gaussian/30% Lorentzian function. All fitting parameters employed in this work are based on previous fittings from our group that are reported in the literature references here for this class of M-N-C catalysts^63–66^. X-ray diffraction (XRD) patterns were collected on a Rigaku Ultima-III powder X-ray diffractometer. Inductively coupled plasma mass spectrometry (ICP-MS) on an Aligent 5110 was performed to accurately quantify the low metal content of the M-N-C catalysts. N_2_ physisorption was recorded on a Micromeritics 3Flex Analyzer at 77 K, using a low-pressure dosing mode (5 cm^3^ g^−1^). The surface area and pore size distribution were obtained using the Brunauer–Emmett–Teller method and the non-local density functional theory model (NLDFT), respectively. Raman spectra were obtained with a 633 nm laser and 600 g.mm grating, using a Horiba LabRAM-HR.

### Preparation of the working electrode%

A catalyst ink was created by mixing 10 mg of catalyst in a solution of 680 μL IPA, 300 μL MilliQ water and 20 μL of a 5 wt% Nafion solution and sonicated for 1 h. For linear sweep voltammetry (LSV) experiments, 12.35 μL of catalyst ink was drop cast on a glassy carbon electrode (0.247 cm^2^) for a catalyst loading of 0.5 mg cm^−2^. For chronoamperometry experiments an AvCarb MGL370 carbon paper was used as the working electrode. The carbon paper was pre-treated by plasma cleaning for 15 min to remove the PTFE layer and then subsequently washed in 3 M H_2_SO_4_ and MilliQ water to create a hydrophilic surface. After the pretreatment, 12.5 μL of catalyst ink was drop cast on the carbon paper with a 0.25 cm^2^ geometric working area for a catalyst loading of 0.5 mg cm^−2^.

### Linear sweep voltammetry

Note that the applied potential for all electrochemical tests is not iR corrected. From PEIS measurements (from 1 MHz to 1 Hz at 7 points per decade), the solution and contact resistances are small at 13.5 Ω, aided by the high concentration of KNO_3_, which dissociates into K^+^ and NO_3_^−^ ions, creating a strong electrolyte with a high conductivity. To evaluate the onset potentials for the HER, NO_2_RR, and NO_3_RR, LSV was performed. All LSV measurements were obtained on a Biologic potentiostat, using a rotating disk electrode in a single compartment cell. A glassy carbon electrode (0.247 cm^2^), graphite rod and reversible hydrogen electrode are used as the working, counter, and reference electrodes, respectively. To evaluate the HER onset potential, a 0.05 M PBS electrolyte was used. For the NO_2_RR onset potential LSV was performed in a 0.05 M PBS + 0.01 M KNO_2_ electrolyte. For the NO_3_RR onset potential LSV was performed in a 0.05 M PBS + 0.16 M KNO_3_ electrolyte. LSV curves were obtained by sweeping reductively from 1 V to −1 V vs. RHE at 5 mV s^−1^ at a rotation speed of 1600 rpm. The reaction onset potential was determined to be the potential at which a current density of 0.4 mA cm^−2^ was reached.

### Electrocatalytic nitrate reduction

Electrochemical nitrate reduction measurements were carried out in a customized two compartment H-cell, as shown in Fig. S66 separated by a Celgard 3401 porous polypropylene membrane (used without pre-treatment). Electrochemical measurements were recorded using an AutoLab potentiostat. A standard three-electrode system was used with a reversible hydrogen electrode (HydroFlex®) and a graphite rod as the reference and counter electrode, respectively. The electrolyte used for all nitrate reduction experiments is a 0.05 M phosphate buffer solution (PBS) and with 0.16 M NO_3_^−^ (KNO_3_). The electrolyte pH was measured to be pH = 6.3 ± 0.05 using an Orion Dual Star pH probe. Error bars are obtained from measurements on three independent electrolytes. Prior to electrochemical tests, N_2_ gas (research grade 99.9995% - PraxAir) is purged in both the working and counter chambers for 30 min at 80 sccm, which contain 30 mL and 25 mL of electrolyte, respectively. To assess if saturating the electrolyte with N_2_ or Ar has an impact on the equilibrium potentials of the NO_3_RR processes, open circuit voltage measurements were performed under N_2_ or Ar saturation and showed negligible differences as shown in Fig. S67. Potentiostatic tests are performed for 2 h under constant stirring at a constant N_2_ gas flow rate of 20 sccm to the working chamber. Potentiostatic tests were performed at potentials of −0.20, −0.40, −0.60 and −0.80 V vs. RHE. After the electrolysis, the electrolyte in both the working and counter chambers was sampled and tested for NH_3_ and NO_2_^−^. Note that at the pH = 6.3 ± 0.05 conditions utilized in this work, the pKa of ammonia has not been reached (pKa 9.2), therefore the ammonia produced is in the protonated form ammonium (NH_4_^+^). However, in the alkaline conditions present during the Berthelot method for detection, the NH_3_/NH_4_^+^ is present in the NH_3_ form. Therefore, the discussion in this manuscript is for the detected product ammonia (NH_3_). Note our previous work has demonstrated that these atomically dispersed M-N-C catalysts have been shown to be inactive for the reduction of N_2_ under these conditions^24^.

NO_3_RR tests in alkaline media (pH = 13.8 ± 0.17) were performed using a 1 M KOH + 0.16 M KNO_3_ electrolyte. Due to the extremely high NH_3_ yield rate, potentiostatic tests were performed for 15 min at potentials of −0.20, −0.40, −0.60, and −0.80 V vs. RHE.

### Electrocatalytic nitrite reduction

Electrochemical nitrite reduction experiments were performed analogously to nitrate reduction experiments. The electrolyte is 0.05 M PBS and 0.01 M NO_2_^−^ (KNO_2_) (pH = 6.26 ± 0.11). Due to the higher NH_3_ yield rates of the more active NO_2_RR, potentiostatic tests were performed for 30 min. Potentiostatic tests were performed at potentials of −0.20, −0.40, −0.60, and −0.80 V vs. RHE. After each electrolysis, the electrolyte in the working chamber was sampled and tested for NH_3_ detection.

### Isotope-labeling experiments

#### Isotopic nitrate ^15^NO_3_^−^ labeling

Isotopic ^15^NO_3_^−^ labeling experiments were performed by using K^15^NO_3_ (99% - Cambridge Isotopes) as the isotopic nitrate source with a 0.05 M PBS + 0.16 M K^15^NO_3_^−^ electrolyte (pH 6.3 ± 0.05). Isotopic ^15^NO_3_^−^ experiments were performed using a metal free N-C catalyst. Using a metal free N-C catalyst simultaneously allows the source of the N in the produced NH_3_ to be confirmed, while also demonstrating that the even the metal free N-moieties in the N-C catalysts can catalyze the NO_3_RR (and is not from N-originating from catalyst decomposition).Electrolysis was performed at −0.40 V for 4 h, after which the electrolyte in the working chamber was samples and the produced ^15^NH_3_ was quantified by ^1^H NMR.

#### Isotopic nitrite ^15^NO_2_^−^ labeling

Isotopic ^15^NO_2_^−^ labeling experiments were performed by using Na^15^NO_3_ (98%^+^ - Cambridge Isotopes) as the isotopic nitrite source. Isotopically labeled ^15^NO_2_^−^ at concentrations of 100, 10, and 1 ppm were doped into the 0.05 M PBS + 0.16 M KNO_3_ (10,000 ppm NO_3_^−^) electrolyte. Potentiostatic tests were performed at −0.40 V vs. RHE for 6 h, under constant stirring and a N_2_ gas flow of 20 sccm to the working chamber. The electrolyte was sampled at 2-, 4-, and 6-h time intervals and the ^15^NH_3_ was quantified by ^1^H NMR.

#### Calculation of the yield and faradaic efficiency

Note that all error bars in this work are determined from triplicate experiments on independently prepared electrodes and are then calculated using a 90% confidence interval.

For the nitrate (NO_3_^−^) and nitrite (NO_2_^−^) reduction reactions, the NH_3_ yield rate was calculated by Eq. (1).

1 $${{Yield}}_{{{NH}}_{3}}=\frac{{c}_{{{NH}}_{3}} * V}{{{Mw}}_{{{NH}}_{3}} * t * {A}_{{electrode}}}$$

For the nitrate (NO_3_^−^) and nitrite (NO_2_^−^) reduction reactions, the NH_3_ Faradaic efficiency was calculated by Eq. (2).

2 $${{FE}}_{{{NH}}_{3}}=\frac{n * F * {c}_{{{NH}}_{3}} * V}{{{Mw}}_{{{NH}}_{3}} * Q}$$

In the NO_3_RR, the nitrate (NO_3_^−^) to nitrite (NO_2_^−^) reduction Faradaic efficiency was calculated by Eq. (3).

3 $${{FE}}_{{{NO}}_{2}^{-}}=\frac{n * F * {c}_{{{NO}}_{2}^{-}} * V}{{{Mw}}_{{{NO}}_{2}^{-}} * Q}$$

where ${{{{{{\rm{c}}}}}}}_{{{{{{{\rm{NH}}}}}}}_{3}}$ is the concentration of NH_3(aq)_ (μg mL^−1^), *V* is the volume of the electrolyte (mL), ${{{{{{\rm{Mw}}}}}}}_{{{{{{{\rm{NH}}}}}}}_{3}}$ is the molar mass of NH_3_ (17.031 g mol^−1^), *t* is the duration of the chronoamperometric measurement (h), the surface area of the working electrode, *A*_electrode_ is 0.45 cm^2^. The number of electrons transferred, *n*, for NO_3_^−^ to NO_2_^−^ is *n* = 2 and for NO_3_^−^ to NH_3_ is *n* = 8.

*F* is Faraday’s constant (96,485 C mol^−1^), the concentration of NO_2_^−^ (μg mL^−1^), C_NO2-_, where the molar mass of NO_2_^−^, Mw_NO2-_ is (46.005 g mol^−1^) and the charge during the chronoamperometric measurement, Q.

#### Product detection

For typical, non-isotopically labeled NO_3_RR and NO_2_RR electrolysis, products were quantified using an ultraviolet-visible (UV–Vis) spectrophotometer (Shimadzu, UV−2600).

#### Determination of ammonia

Ammonia was quantified by UV–Vis using the Berthelot reaction. In cases producing large concentrations of NH_3_, the working electrolyte solution was diluted such that the absorbance would fall within the range of the calibration curve. Specifically, 2 mL of the electrolyte (or diluted electrolyte if needed) was pipetted into a vial. To this, 2 mL of a 1 M NaOH solution that contains 5 wt% salicylic acid, and 5 wt% sodium citrate was added. Next, 1 mL of a 0.05 M NaClO and 0.2 mL of a 1 wt% C_5_FeN_6_Na_2_O (sodium nitroferricyanide) was added to the solution. The solution was incubated in the dark at ambient condition for 1 h, then UV-Vis spectra were recorded. The concentration of NH_3_ is determined using the maximum absorbance at a 655 nm wavelength, when compared to the generated calibration curves, Figs. S68–69.

#### Determination of nitrite

Nitrite was quantified by UV–Vis using a commercial NO_2_^−^ assay kit (Spectroquant), based on the Griess test. In cases producing large concentrations of NO_2_^−^, the working electrolyte solution was diluted such that the absorbance would fall within the range of the calibration curve. Specifically, 2 mL of the electrolyte (or diluted electrolyte if needed) was placed in a vial. To this, 22 mg of the assay reagent was added to the electrolyte and incubated in the dark at ambient conditions for 10 min. After incubation, the UV–Vis spectra were taken, and the concentration of nitrite was determined at a maximum absorbance of 540 nm wavelength, when compared to the generated calibration curves, Fig. S70.

#### NMR determination of ammonia

For isotopic labeling experiments, nuclear magnetic resonance (NMR) spectroscopy was used to detect and quantify both ^14^NH_3_ and ^15^NH_3_. Dimethylsulfoxide-d6 (DMSO) and 3-(trimethylsilyl)-1-propanesulfonic acid sodium salt (DSS) were used as the locking solvent and internal standard, respectively. For the NMR test solution, 580 μL of the electrolysis electrolyte, 25 μL of DMSO, 20 μL of 3 M H_2_SO_4_, and 75 μL of 6 mM DSS (made with Millipore water) are mixed. The NMR spectrum was obtained on a Bruker CRYO 500 MHz spectrometer. The signal from H_2_O was restrained for better accuracy by applying the solvent suppression method during acquisition. Topspin 4.0.8 software was used to process the NMR data. Standard NMR calibration curves for standard ^14^NH_3_ and isotopically labeled ^15^NH_3_ are shown in Fig. S71.

#### Computational details

All the DFT calculations were performed with the generalized gradient approximation approach and projector augmented-wave pseudopotentials^67,68^ using the Vienna Ab initio Simulation Package^69–71^. To account for the van der Waals interactions, an optB86b-vdW functional was used^72–75^ with a gamma centered 8 × 8 × 1 (M-N_4_ sites, pyridinic N, graphitic N) or 3 × 3 × 1 *k*-mesh (pyrrolic NH) and Fermi-smearing. Sigma and the plane-wave basis cutoff was set to 0.03 and 400 eV, respectively. We approached modeling of the M-N_4_ sites in the M-N-C catalysts by creating two carbon atom vacancies in the graphene, in which we place a metal atom^27,65,76–78^. Structures were then optimized as neutral, using unrestricted open shell spin density and with careful evaluation of the effect the initial magnetic moment has on the self-consistent solution of the electronic problem^79,80^. Adsorption energies are given relative to the M-N_4_ defect with a total magnetic moment corresponding to the lowest electronic energy. M-N_4_, pyridinic N, and graphitic N sites were modeled using 4 × 4 orthorhombic single-layer graphene cell with the dimensions of 9.84 × 8.52 Å. Pyrrolic NH defect was modeled using a hexagonal single layer 17.04 × 17.04 Å unit cell. Structures of all the unit cells used for the DFT calculations are shown on Fig. S72. A vacuum region of 20 Å was applied in all cases. All structures were optimized by allowing all atoms to relax (including adsorbents) while the cell parameters were kept fixed at the DFT optimized value for graphene. The criteria for the convergence of the electronic energy were set to 1 × 10^−5^ eV. The forces were converged to 0.01 eV Å^−1^. All calculations were performed as spin polarized calculations. The Pearson correlation coefficient was calculated using the python function np.corrcoef, which utilizes the equation below.

$$R=\frac{\sum \left({x}_{i}-\bar{x}\right) * ({y}_{i}-\bar{y})}{\sqrt{\sum {({x}_{i}-\bar{x})}^{2} * \sum {({y}_{i}-\bar{y})}^{2}}}$$

Where *R*, is the correlation coefficient, *x*_*i*_ and *y*_*i*_ are the *x* and *y* values in samples, and ${\bar{x}}$ and ${\bar{y}}$ are mean values for the *x* and *y* variables. The computational approach for the calculation of Gibbs free energy of reactions and considered steps in the NO_3_RR and NO_2_RR mechanism are explained in details of our recent work^24^. However, it must be pointed out that all the Gibbs free energies were calculated at an experimentally determined pH of 6.3. We also emphasize that M-N-C catalysts are challenging to simulate, and the reliability of the computational results depends on many factors (e.g., see refs. ^81,82^ and SI note 4). Although one should pay attention to the accuracy of the absolute numbers, relative values and trends are more reliable.

## Supplementary information

Supplementary Information

Peer Review File

## Data Availability

The computational data for Figures and Supplementary Figures is available in X repository. All other relevant data supporting for the findings in this work are available from the authors upon request.

## References

[1] Schiffer ZJ, Manthiram K (2017). Electrification and decarbonization of the chemical industry. Joule.

[2] van Geem KM, Galvita VV, Marin GB (2019). Making chemicals with electricity. Science.

[3] Chen, J. G. et al. Beyond fossil fuel–driven nitrogen transformations. *Science* **360**, eaar6611 (2018). 10.1126/science.aar6611 PMC6088796 29798857

[4] Iriawan, H. et al. Methods for nitrogen activation by reduction and oxidation. *Nat. Rev. Methods Prim.* **1**, 56 (2021).

[5] Ren Y (2021). Strategies to suppress hydrogen evolution for highly selective electrocatalytic nitrogen reduction: challenges and perspectives. Energy Environ. Sci..

[6] Suryanto BHR (2019). Challenges and prospects in the catalysis of electroreduction of nitrogen to ammonia. Nat. Catal..

[7] Choi J (2020). Identification and elimination of false positives in electrochemical nitrogen reduction studies. Nat. Commun..

[8] van Langevelde PH, Katsounaros I, Koper MTM (2021). Electrocatalytic nitrate reduction for sustainable ammonia production. Joule.

[9] Wang Y, Wang C, Li M, Yu Y, Zhang B (2021). Nitrate electroreduction: mechanism insight,: in situ characterization, performance evaluation, and challenges. Chem. Soc. Rev..

[10] Ko BH, Hasa B, Shin H, Zhao Y, Jiao F (2022). Electrochemical reduction of gaseous nitrogen oxides on transition metals at ambient conditions. J. Am. Chem. Soc..

[11] Long J (2020). Direct electrochemical ammonia synthesis from nitric oxide. Angew. Chem. Int. Ed..

[12] Sun J (2021). A hybrid plasma electrocatalytic process for sustainable ammonia production. Energy Environ. Sci..

[13] Muzammil I (2021). Plasma catalyst-integrated system for ammonia production from H2O and N2 at atmospheric pressure. ACS Energy Lett..

[14] Ornes S (2021). Green ammonia could produce climate-friendly ways to store energy and fertilize farms. Proc. Natl. Acad. Sci..

[15] Wang Z, Richards D, Singh N (2021). Recent discoveries in the reaction mechanism of heterogeneous electrocatalytic nitrate reduction. Catal. Sci. Technol..

[16] Liu JX, Richards D, Singh N, Goldsmith BR (2019). Activity and selectivity trends in electrocatalytic nitrate reduction on transition metals. ACS Catal..

[17] Yang M (2022). Tuning single metal atoms anchored on graphidyne for highly efficient and selective nitrate electroreduction to ammonia under aqueous environments: a computational study. Appl. Surf. Sci..

[18] Wang Y (2020). Enhanced nitrate-to-ammonia activity on copper-nickel alloys via tuning of intermediate adsorption. J. Am. Chem. Soc..

[19] Seraj S (2017). PdAu Alloy nanoparticle catalysts: effective candidates for nitrite reduction in water. ACS Catal..

[20] Wang Z, Young SD, Goldsmith BR, Singh N (2021). Increasing electrocatalytic nitrate reduction activity by controlling adsorption through PtRu alloying. J. Catal..

[21] Milton RD, Minteer SD (2017). Enzymatic bioelectrosynthetic ammonia production: recent electrochemistry of nitrogenase, nitrate reductase, and nitrite reductase. Chempluschem.

[22] Thorgersen MP (2015). Molybdenum availability is key to nitrate removal in contaminated groundwater environments. Appl. Environ. Microbiol..

[23] Wang J (2021). Electrocatalytic nitrate/nitrite reduction to ammonia synthesis using metal nanocatalysts and bio-inspired metalloenzymes. Nano Energy.

[24] Murphy E (2022). Highly durable and selective Fe- and Mo-based atomically dispersed electrocatalysts for nitrate reduction to ammonia via distinct and synergized no 2 – pathways. ACS Catal..

[25] He W (2022). Splicing the active phases of copper/cobalt-based catalysts achieves high-rate tandem electroreduction of nitrate to ammonia. Nat. Commun..

[26] Carvalho OQ (2022). Role of electronic structure on nitrate reduction to ammonium: a periodic journey. J. Am. Chem. Soc..

[27] Asset T (2019). Investigating the nature of the active sites for the CO_2_ reduction reaction on carbon-based electrocatalysts. ACS Catal..

[28] Ju W (2017). Understanding activity and selectivity of metal-nitrogen-doped carbon catalysts for electrochemical reduction of CO_2_. Nat. Commun..

[29] Delafontaine L (2022). Synergistic electrocatalytic syngas production from carbon dioxide by bi-metallic atomically dispersed catalysts. Chem. Electro Chem..

[30] Rojas-Carbonell S, Santoro C, Serov A, Atanassov P (2017). Transition metal-nitrogen-carbon catalysts for oxygen reduction reaction in neutral electrolyte. Electrochem. commun..

[31] Jin Z (2021). Understanding the inter-site distance effect in single-atom catalysts for oxygen electroreduction. Nat. Catal..

[32] Wu Z (2021). Electrochemical ammonia synthesis via nitrate reduction on Fe single atom catalyst. Nat. Commun..

[33] Li P, Jin Z, Fang Z, Yu G (2021). A single-site iron catalyst with preoccupied active centers that achieves selective ammonia electrosynthesis from nitrate. Energy Environ. Sci..

[34] Serov A (2015). Nano-structured non-platinum catalysts for automotive fuel cell application. Nano Energy.

[35] Hossen MM, Artyushkova K, Atanassov P, Serov A (2018). Synthesis and characterization of high performing Fe-N-C catalyst for oxygen reduction reaction (ORR) in alkaline exchange membrane fuel cells. J. Power Sources.

[36] Serov A, Artyushkova K, Andersen NI, Stariha S, Atanassov P (2015). Original mechanochemical synthesis of non-platinum group metals oxygen reduction reaction catalysts assisted by sacrificial support method. Electrochim. Acta.

[37] Asset T, Atanassov P (2020). Iron-nitrogen-carbon catalysts for proton exchange membrane. Fuel Cells Joule.

[38] Li J (2020). Efficient ammonia electrosynthesis from nitrate on strained ruthenium nanoclusters. J. Am. Chem. Soc..

[39] Wang Y, Shao M (2022). Theoretical screening of transition metal-n4-doped graphene for electroreduction of nitrate. ACS Catal..

[40] Niu H (2020). Theoretical insights into the mechanism of selective nitrate-to-ammonia electroreduction on single-atom catalysts. Adv. Funct. Mater..

[41] Wang S (2022). High-throughput identification of highly active and selective single-atom catalysts for electrochemical ammonia synthesis through nitrate reduction. Nano Energy.

[42] Liu H (2021). Electrocatalytic nitrate reduction on oxide-derived silver with tunable selectivity to nitrite and ammonia. ACS Catal..

[43] Yang J (2022). Potential-driven restructuring of cu single atoms to nanoparticles for boosting the electrochemical reduction of nitrate to ammonia. J. Am. Chem. Soc..

[44] Weng Z (2018). Active sites of copper-complex catalytic materials for electrochemical carbon dioxide reduction. Nat. Commun..

[45] Wang M (2019). Over 56.55% faradaic efficiency of ambient ammonia synthesis enabled by positively shifting the reaction potential. Nat. Commun..

[46] Lu C (2021). Electrochemical reduction of carbon dioxide with nearly 100% carbon monoxide faradaic efficiency from vacancy-stabilized single-atom active sites. J. Mater. Chem. A.

[47] Yang HB (2018). Atomically dispersed Ni(i) as the active site for electrochemical CO_2_ reduction. Nat. Energy.

[48] He, Q. et al. Accelerating CO_2_ electroreduction to CO Over Pd single-atom catalyst. *Adv. Funct. Mater*. **30**, 2000407 (2020).

[49] Leonard N (2018). The chemical identity, state and structure of catalytically active centers during the electrochemical CO_2_ reduction on porous Fe-nitrogen-carbon (Fe-N-C) materials. Chem. Sci..

[50] Zou H, Rong W, Wei S, Ji Y, Duan L (2020). Regulating kinetics and thermodynamics of electrochemical nitrogen reduction with metal single-atom catalysts in a pressurized electrolyser. Proc. Natl. Acad. Sci. USA.

[51] Tao H (2019). Nitrogen fixation by ru single-atom electrocatalytic reduction. Chem.

[52] Liu J (2020). Rare earth single-atom catalysts for nitrogen and carbon dioxide reduction. ACS Nano.

[53] Han L (2019). Atomically dispersed molybdenum catalysts for efficient ambient nitrogen fixation. Angew. Chem. Int. Ed..

[54] Han L (2021). Local modulation of single-atomic mn sites for enhanced ambient ammonia electrosynthesis. ACS Catal..

[55] Chen GF (2020). Electrochemical reduction of nitrate to ammonia via direct eight-electron transfer using a copper–molecular solid catalyst. Nat. Energy.

[56] Wheeldon I (2016). Substrate channelling as an approach to cascade reactions. Nat. Chem..

[57] Li J, Zhang Y, Kuruvinashetti K, Kornienko N (2022). Construction of C–N bonds from small-molecule precursors through heterogeneous electrocatalysis. Nat. Rev. Chem..

[58] Wu Y, Jiang Z, Lin Z, Liang Y, Wang H (2021). Direct electrosynthesis of methylamine from carbon dioxide and nitrate. Nat. Sustain..

[59] Wang Y, Shao M (2022). Theoretical screening of transition metal–N 4 -doped graphene for electroreduction of nitrate. ACS Catal..

[60] Moltved KA, Kepp KP (2019). The chemical bond between transition metals and oxygen: electronegativity, d-orbital effects, and oxophilicity as descriptors of metal-oxygen interactions. J. Phys. Chem. C..

[61] Ravel B, Newville M (2005). ATHENA, ARTEMIS, HEPHAESTUS: data analysis for X-ray absorption spectroscopy using IFEFFIT. J. Synchrotron Radiat..

[62] Ankudinov A, Conradson S, Mustre de Leon J (1998). Relativistic XANES calculations of Pu hydrates. Phys. Rev. B Condens. Matter Mater. Phys..

[63] Artyushkova K, Serov A, Rojas-Carbonell S, Atanassov P (2015). Chemistry of multitudinous active sites for oxygen reduction reaction in transition metal-nitrogen-carbon electrocatalysts. J. Phys. Chem. C..

[64] Artyushkova K, Matanovic I, Halevi B, Atanassov P (2017). Oxygen binding to active sites of Fe-N-C ORR electrocatalysts observed by ambient-pressure XPS. J. Phys. Chem. C..

[65] Matanovic I, Artyushkova K, Atanassov P (2018). Understanding PGM-free catalysts by linking density functional theory calculations and structural analysis: perspectives and challenges. Curr. Opin. Electrochem..

[66] Artyushkova K (2020). Misconceptions in interpretation of nitrogen chemistry from x-ray photoelectron spectra. J. Vac. Sci. Technol. A.

[67] Blöchl PE, Jepsen O, Andersen OK (1994). Improved tetrahedron. Phys. Rev. B.

[68] Joubert D (1999). From ultrasoft pseudopotentials to the projector augmented-wave method. Phys. Rev. B Condens. Matter Mater. Phys..

[69] Kresse G, Hafner J (1993). Ab initio molecular dynamics for liquid metals. Phys. Rev. B.

[70] Kresse G, Hafner J (1994). Ab initio molecular-dynamics simulation of the liquid-metalamorphous- semiconductor transition in germanium. Phys. Rev. B.

[71] Kresse G, Furthmüller J (1996). Efficiency of ab-initio total energy calculations for metals and semiconductors using a plane-wave basis set. Comput. Mater. Sci..

[72] Dion M, Rydberg H, Schröder E, Langreth DC, Lundqvist BI (2004). Van der Waals density functional for general geometries. Phys. Rev. Lett..

[73] Klime J, Bowler DR, Michaelides A (2011). Van der Waals density functionals applied to solids. Phys. Rev. B Condens. Matter Mater. Phys..

[74] Román-Pérez G, Soler JM (2009). Efficient implementation of a van der waals density functional: application to double-wall carbon nanotubes. Phys. Rev. Lett..

[75] Klimeš J, Bowler DR, Michaelides A (2010). Chemical accuracy for the van der Waals density functional. J. Phys. Condens. Matter.

[76] Rojas-Carbonell S (2018). Effect of pH on the activity of platinum group metal-free catalysts in oxygen reduction reaction. ACS Catal..

[77] Kodali M (2017). Air breathing cathodes for microbial fuel cell using Mn-, Fe-, Co- and Ni-containing platinum group metal-free catalysts. Electrochim. Acta.

[78] Sebastián D (2017). Insights on the extraordinary tolerance to alcohols of Fe-N-C cathode catalysts in highly performing direct alcohol fuel cells. Nano Energy.

[79] Li J (2021). Identification of durable and non-durable FeNx sites in Fe–N–C materials for proton exchange membrane fuel cells. Nat. Catal..

[80] Mineva T (2019). Understanding active sites in pyrolyzed Fe-N-C catalysts for fuel cell cathodes by bridging density functional theory calculations and 57fe mössbauer spectroscopy. ACS Catal..

[81] Tosoni S, Di Liberto G, Matanovic I, Pacchioni G (2023). Modelling single atom catalysts for water splitting and fuel cells: a tutorial review. J. Power Sources.

[82] Di Liberto G, Cipriano LA, Pacchioni G (2022). Universal principles for the rational design of single atom electrocatalysts? handle with care. ACS Catal..

