# Supplementary material for: Elucidating electrochemical nitrate and nitrite reduction over atomically-dispersed transition metal sites
Source: Nat Commun. 2023 Jul 28;14:4554. doi: 10.1038/s41467-023-40174-4 (PMC10382506; doi:10.1038/s41467-023-40174-4)
Supplement: Supplementary file 1 — Supplementary Information [file 41467_2023_40174_MOESM1_ESM.pdf]

Supplementary Information

Elucidating electrochemical nitrate and nitrite reduction over atomically-dispersed transition metal sites

Eamonn Murphy[1†], Yuanchao Liu[1†], Ivana Matanovic[2,3], Martina Rüscher[4], Ying Huang[5], Alvin Ly[5], Shengyuan Guo[1], Wenjie Zang[5], Xingxu Yan[5], Andrea Martini[4], Janis Timoshenko[4], Beatriz Roldán Cuenya[4], Iryna V. Zenyuk[1,5], Xiaoqing Pan[5], Erik Spoerke[6] and Plamen Atanassov[1,5*]

[1]Department of Chemical and Biomolecular Engineering, University of California, Irvine, California 92697, USA

[2]Department of Chemical and Biological Engineering, University of New Mexico, Albuquerque, New Mexico 87131, USA

[3]Theoretical Division, Los Alamos National Laboratory, New Mexico 87545, USA

[4]Department of Interface Science, Fritz Haber Institute of the Max Planck Society, 4-6 Faradayweg, Berlin 14195, Germany

[5]Department of Materials Science and Engineering, University of California, Irvine, California 92697, USA

[6]Sandia National Laboratories, Albuquerque, New Mexico 87185, USA

[†]Authors contributed equally

*E-mail: plamen.atanassov@uci.edu

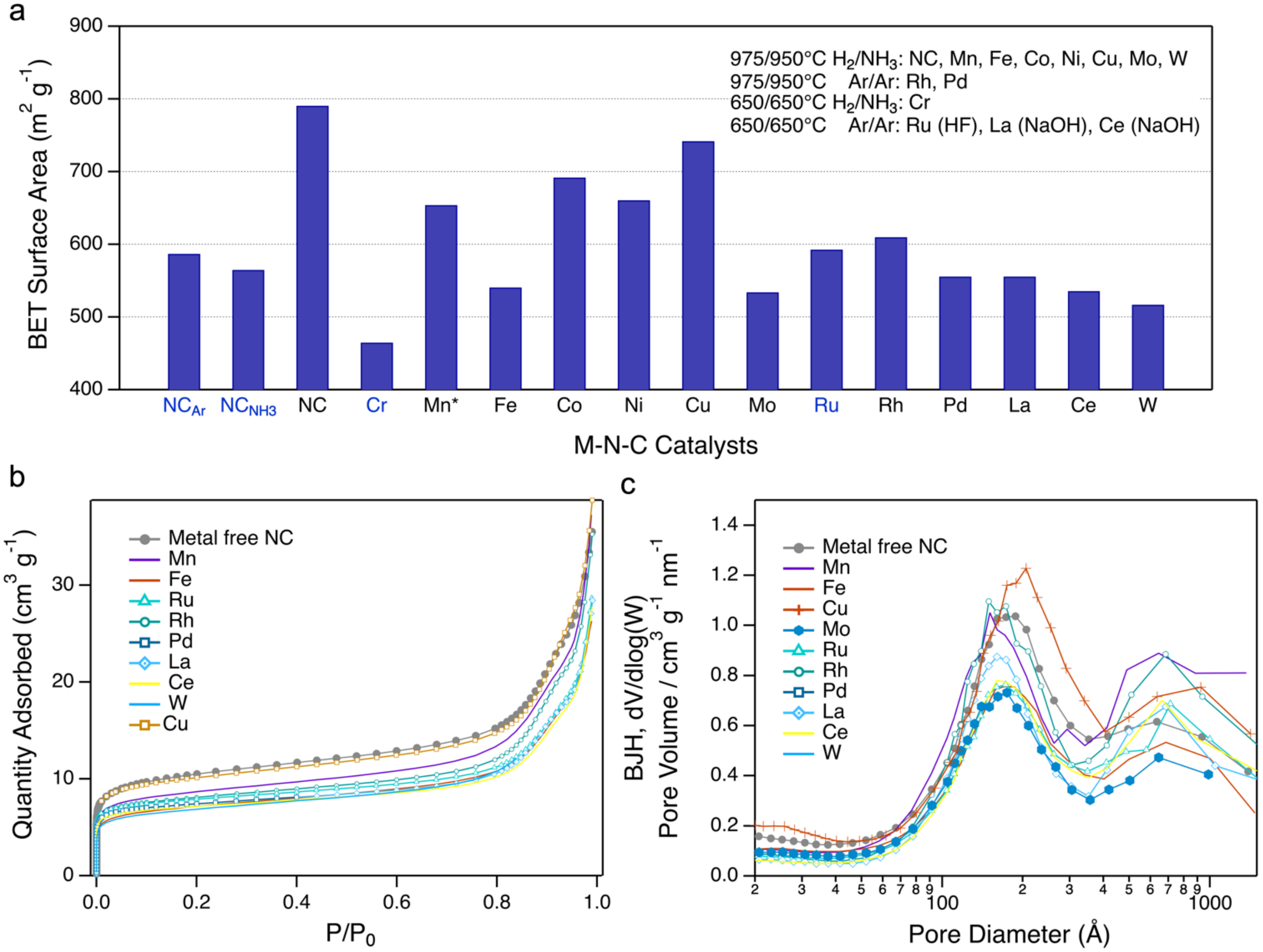

Figure S 1. N_2 physisorption measurements for the M-N-C and metal free N-C catalysts. (a) BET surface area of the metal free N-C and M-N-C catalysts. The surface area of all M-N-C catalysts falls between 550 and 750 m^2/g. (b) N_2 sorption isotherm for the metal free N-C and M-N-C Catalysts. (c) pore size distribution in the meso/macro-porous range, showing distinct microporosity at *ca.* 20 nm and *ca.* 80 nm for all M-N-C catalysts, characteristic of the diameter of the sacrificial silica template.

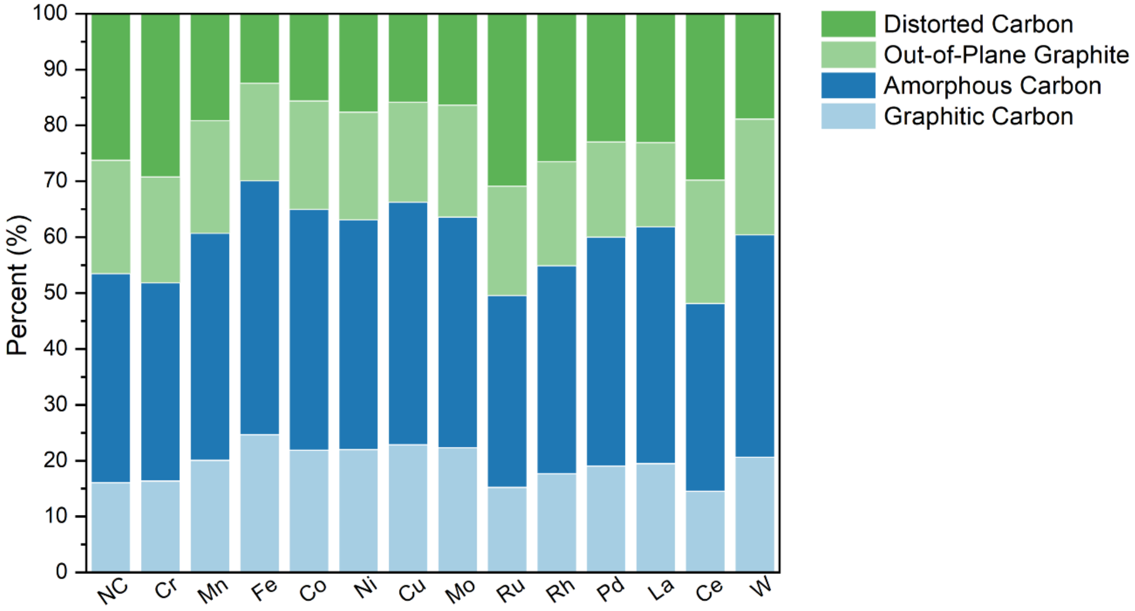

Figure S 2. Carbon composition of the M-N-C catalysts as determined by deconvoluted Raman spectra of the D and G bands. Demonstrating similar carbon composition of all the M-N-C catalysts.

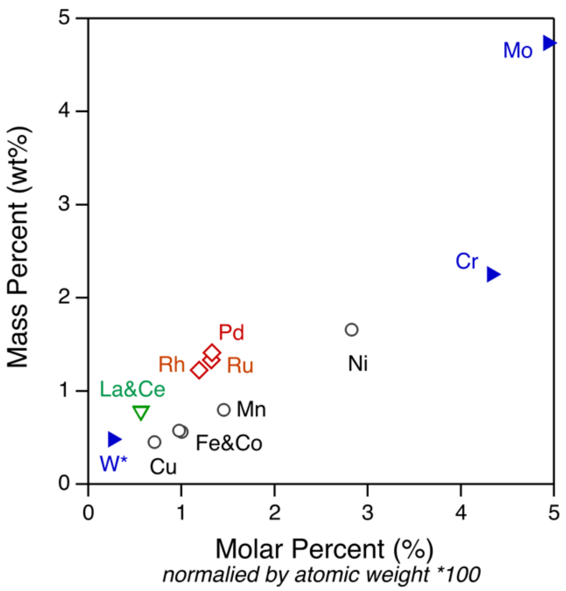

Figure S 3. Metal loading of M-N-C catalysts determined by ICP-MS. The Y-axis is the mass percent as determined by the ICP-MS, while the X-axis is normalized by the molar mass of the metal element.

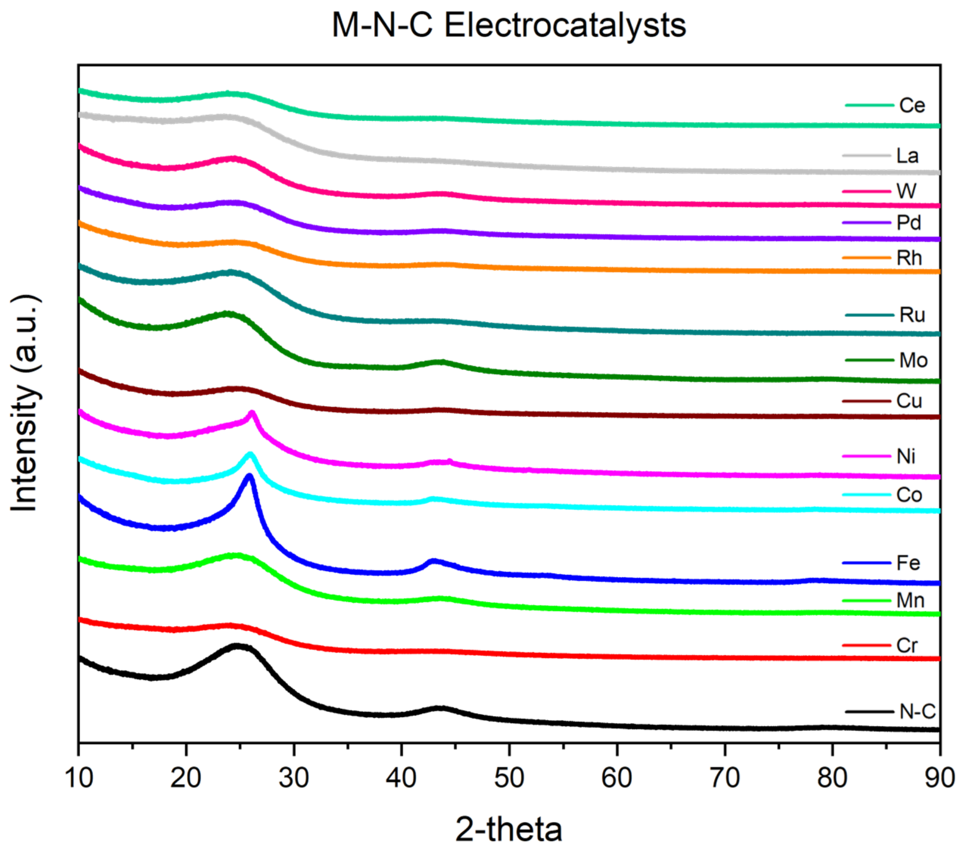

Figure S 4. X-ray diffraction patterns of the metal free N-C and M-N-C catalysts. All materials show only the (002) and (100) graphitic peaks, indicating the absence of any metallic crystalline phases in the materials, characteristic of atomically dispersed materials.

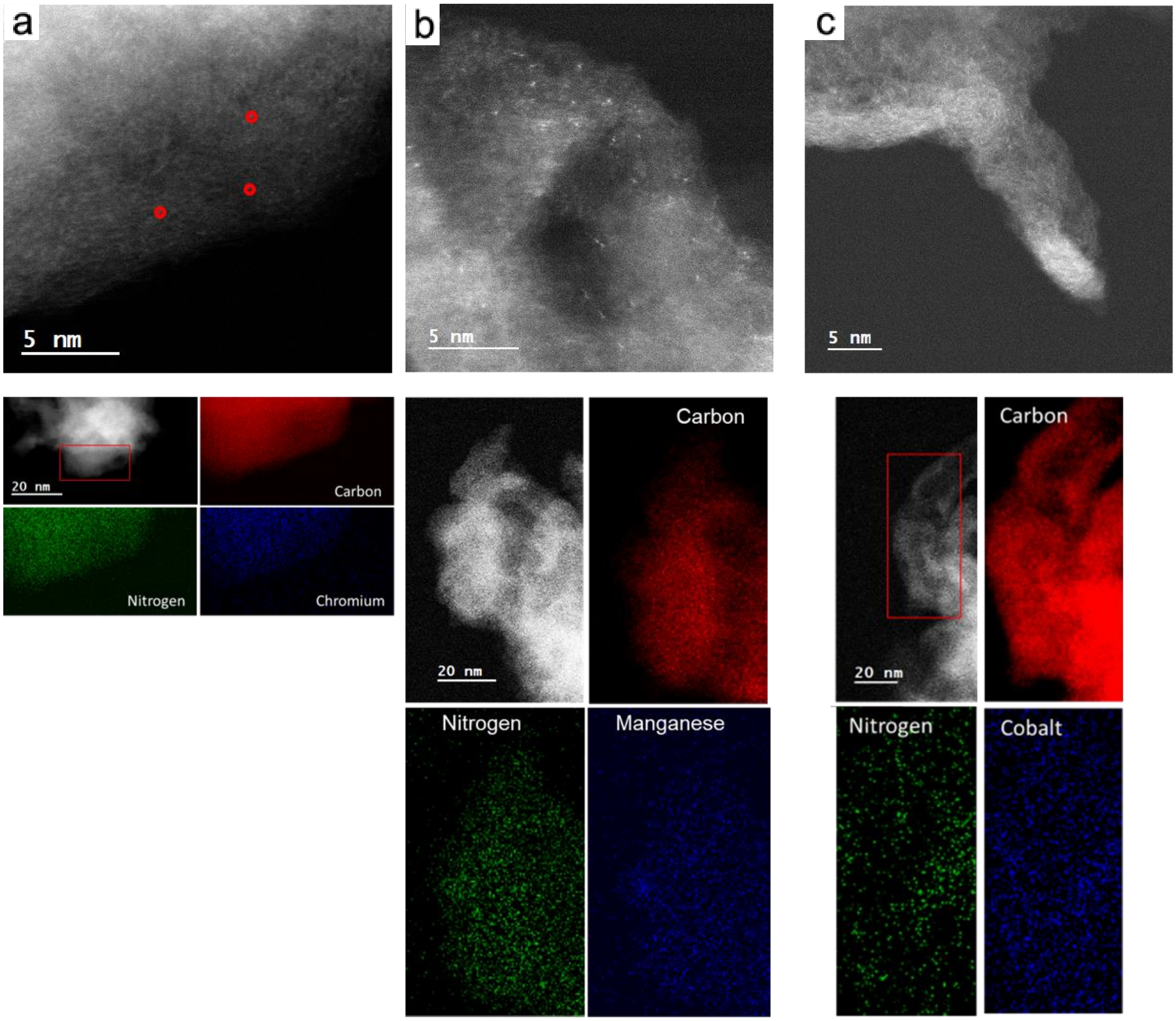

Figure S 5. AC-HAADF STEM images and corresponding EDS mapping of the carbon, nitrogen and metal elements for the (a) Cr-N-C (b) Mn-N-C and (c) Co-N-C catalysts. Demonstrating the atomically dispersed metal sites and homogenous distribution of C, N and metal in the materials.

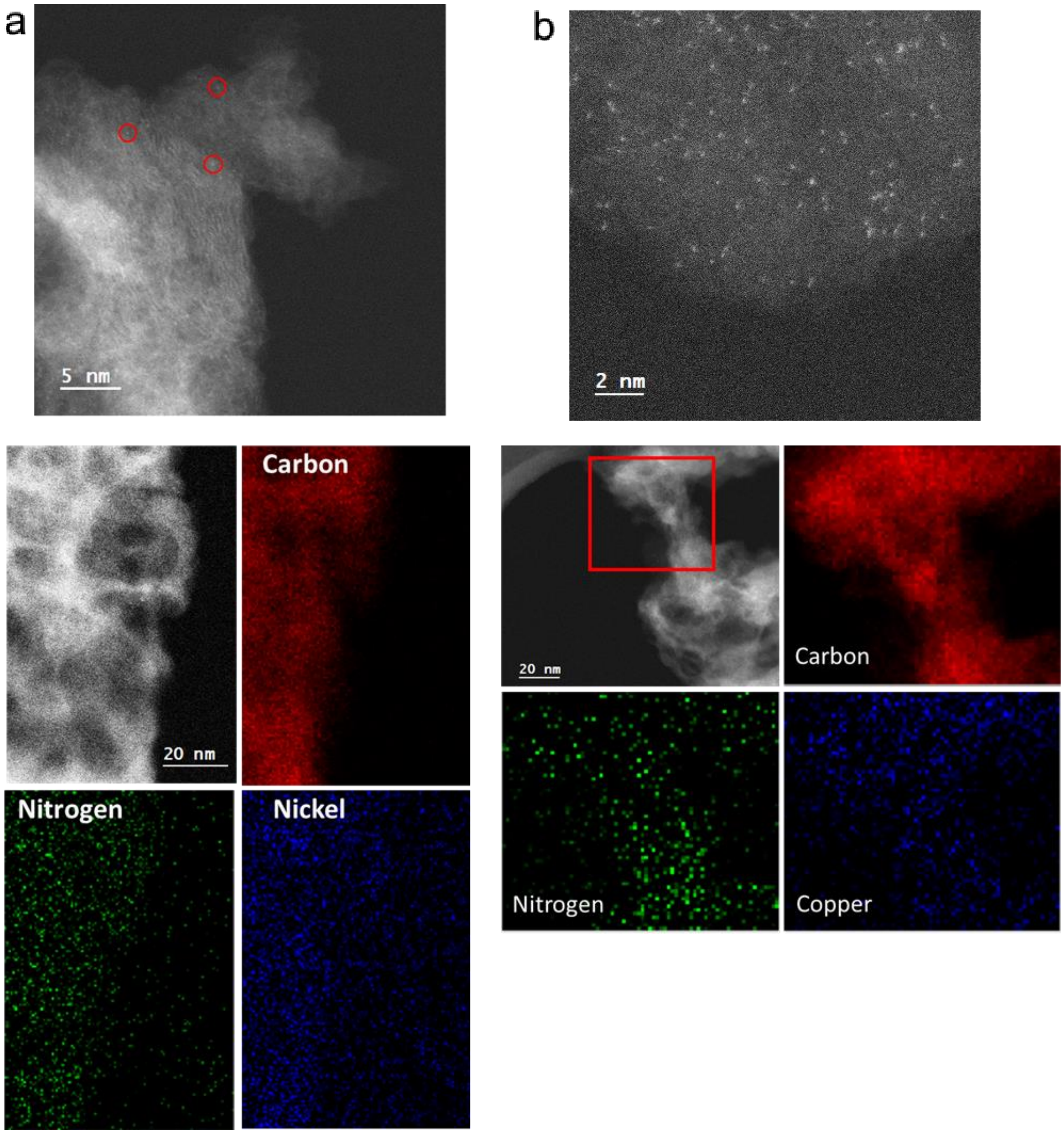

Figure S 6. AC-HAADF STEM images and corresponding EDS mapping of the carbon, nitrogen and metal elements for the (a) Ni-N-C and (b) Cu-N-C. Demonstrating the atomically dispersed metal sites and homogenous distribution of C, N and metal in the materials.

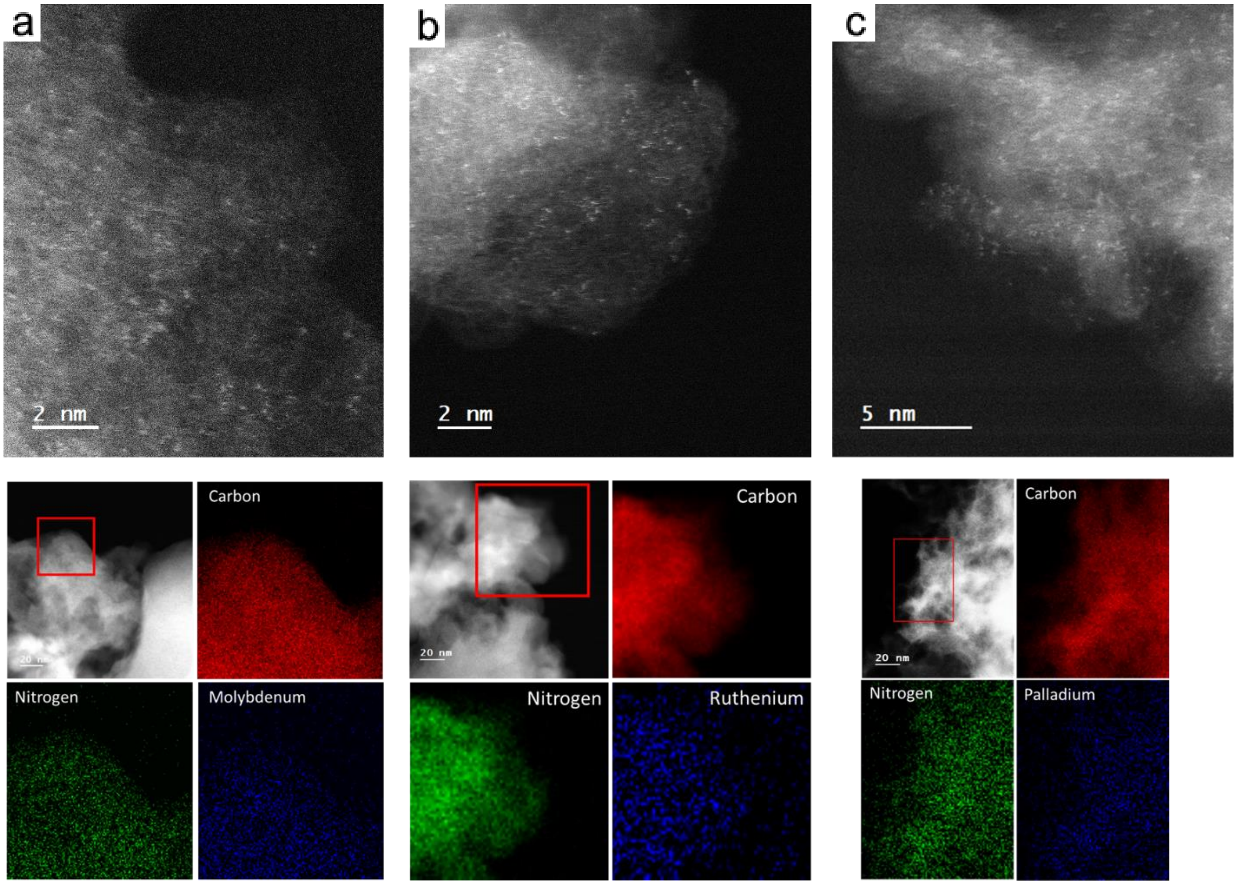

Figure S 7. AC-HAADF STEM images and corresponding EDS mapping of the carbon, nitrogen and metal elements for the (a) Mo-N-C (b) Ru-N-C and (c) Pd-N-C. Demonstrating the atomically dispersed metal sites and homogenous distribution of C, N and metal in the materials.

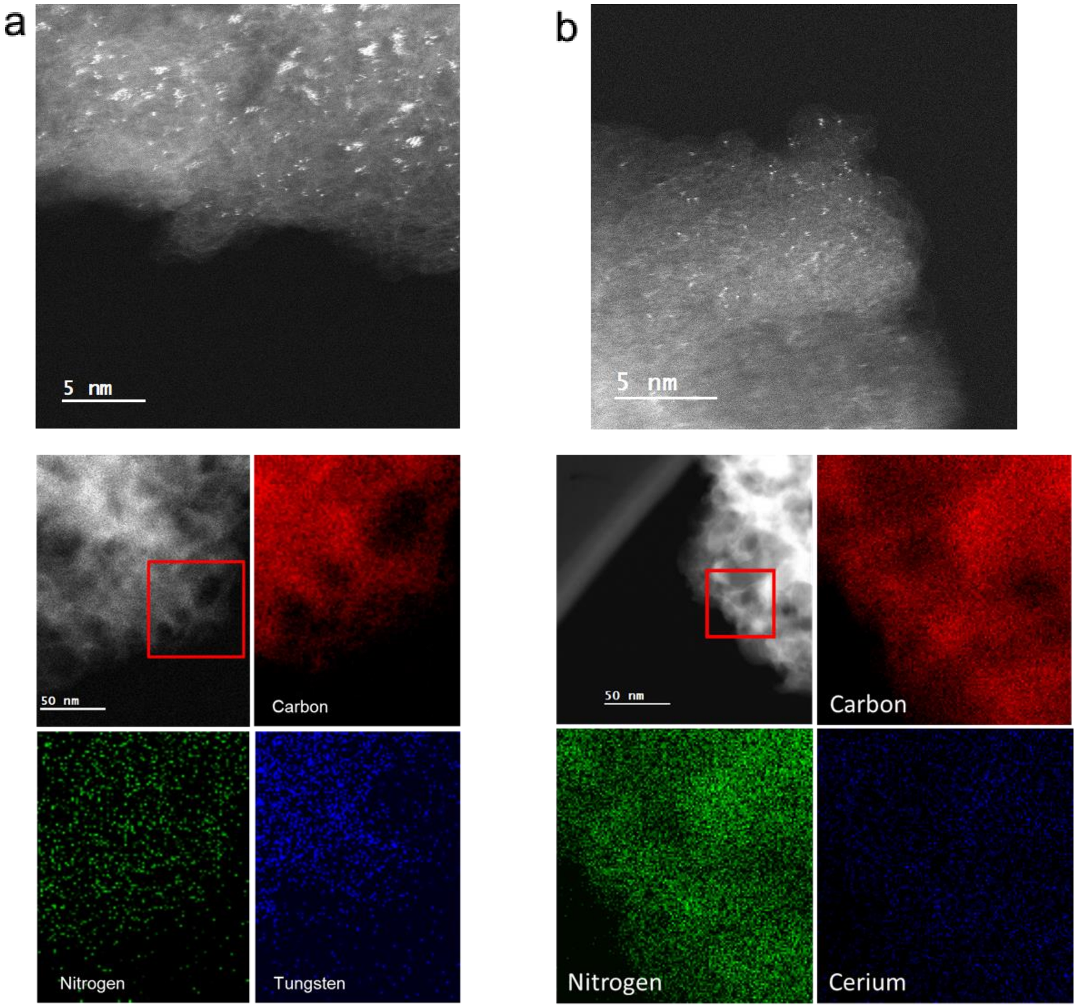

Figure S 8. AC-HAADF STEM images and corresponding EDS mapping of the carbon, nitrogen and metal elements for the (a) W-N-C and (b) Ce-N-C. It can be seen that for W-N-C, while isolated atomically dispersed sites are present, the formation of 2-D nanoclusters are also present.

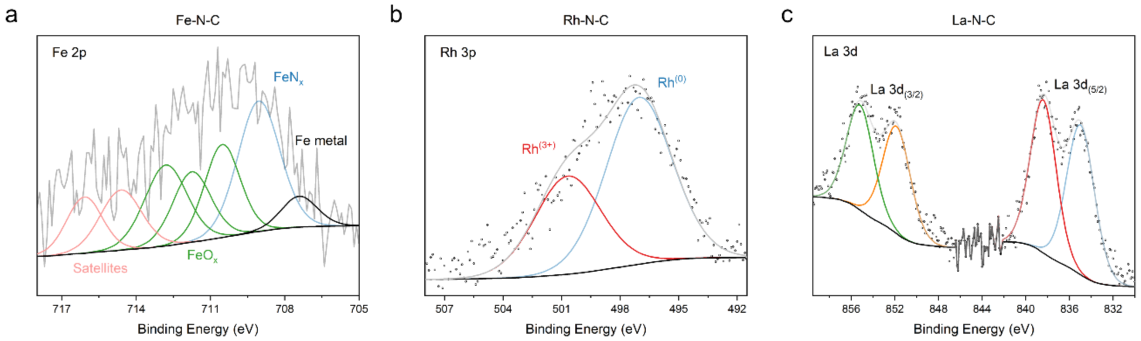

Figure S 9. Deconvoluted XPS spectra for (a) Fe-N-C, showing a mixed oxidation state characteristic of Fe^{2+} and Fe^{3+}. (b) Rh-N-C, showing a mix between Rh^{0} and Rh^{3+}, equating to an oxidation state of ca. $Rh^{1.7+}$. (c) La-N-C, showing an oxidation state of La^{3+}.

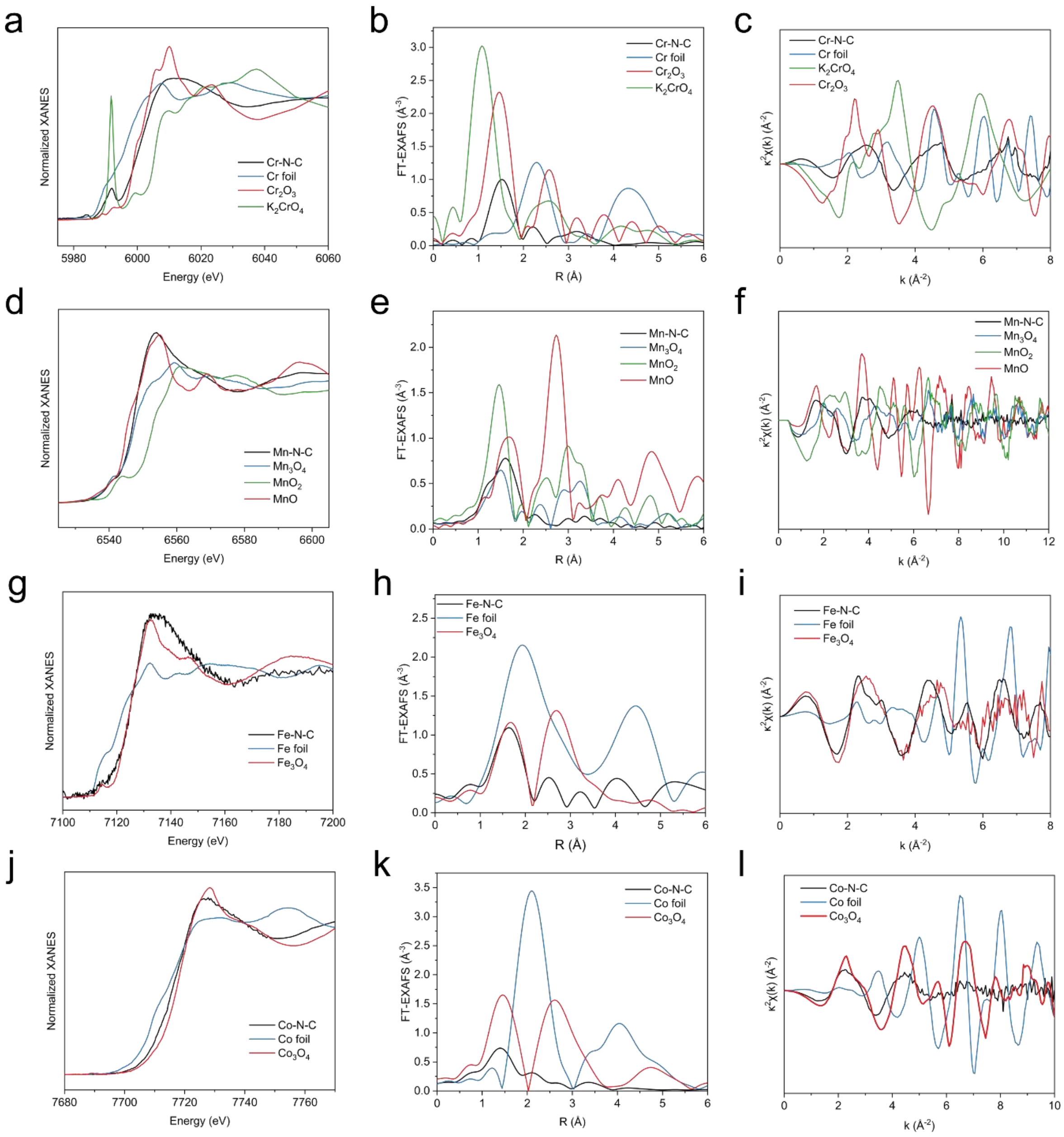

Figure S 10. XANES, FT-EXAFS and EXAFS spectra for the *3d* metals (a-c) Cr-K edge in Cr-N-C, (d-f) Mn-K edge in Mn-N-C, (g-i) Fe-K edge in Fe-N-C and (j-l) Co-K edge in Co-N-C. See Supplementary note 1 for a full discussion on the XANES interpretation and determination of metal oxidation states and Supplementary Table 2 and Supplementary note 3 for a full discussion on the EXAFS analysis.

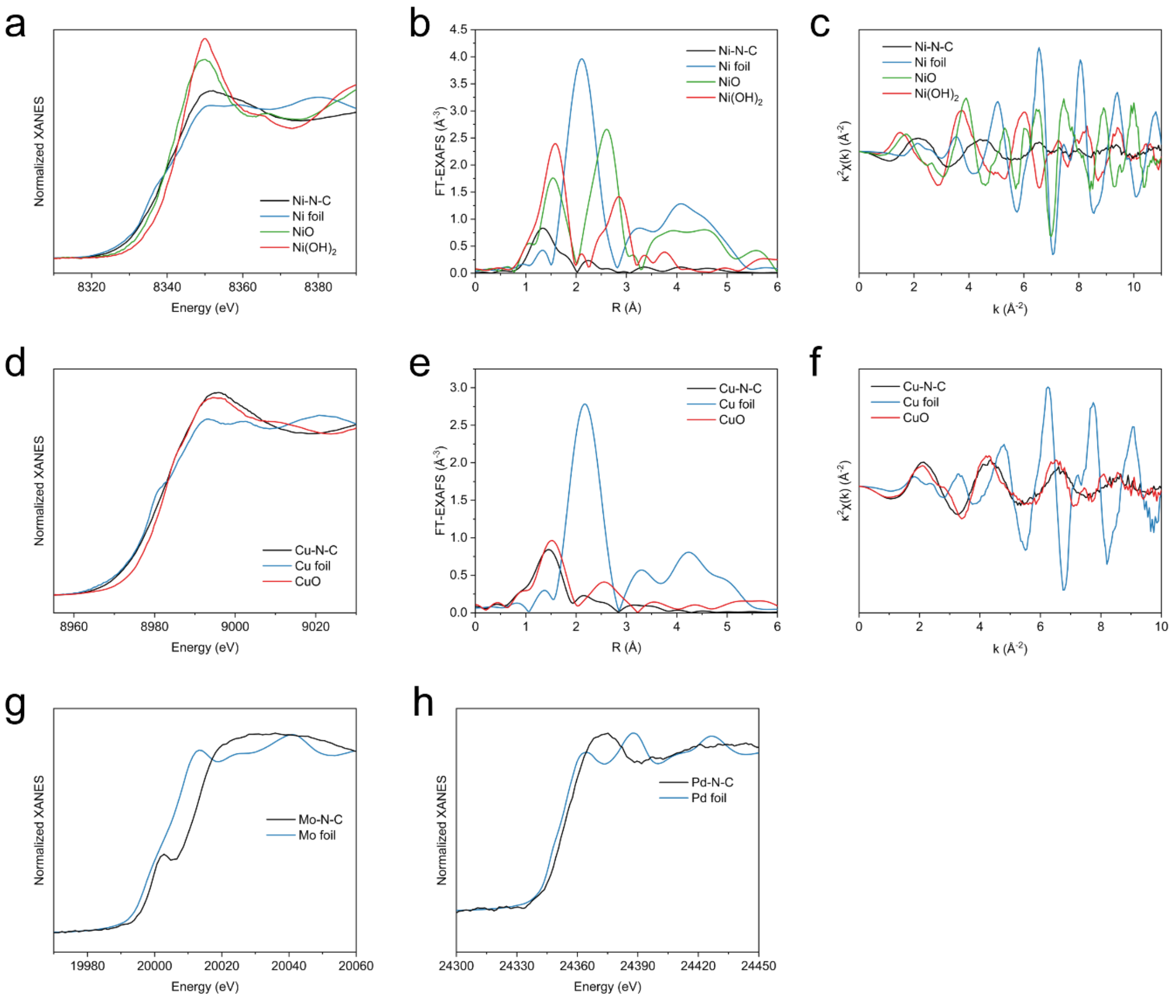

Figure S 11. XANES, FT-EXAFS and EXAFS spectra *3d* metals (a-c) Ni-K edge in Ni-N-C and (d-f) Cu-K edge in Cu-N-C and XANES spectra for *4d* metals (g) Mo-K edge in Mo-N-C and (h) Pd-K edge in Pd-N-C. See Supplementary note 1 for a full discussion on the XANES interpretation and determination of metal oxidation states and Supplementary Table 2 and Supplementary note 3 for a full discussion on the EXAFS analysis.

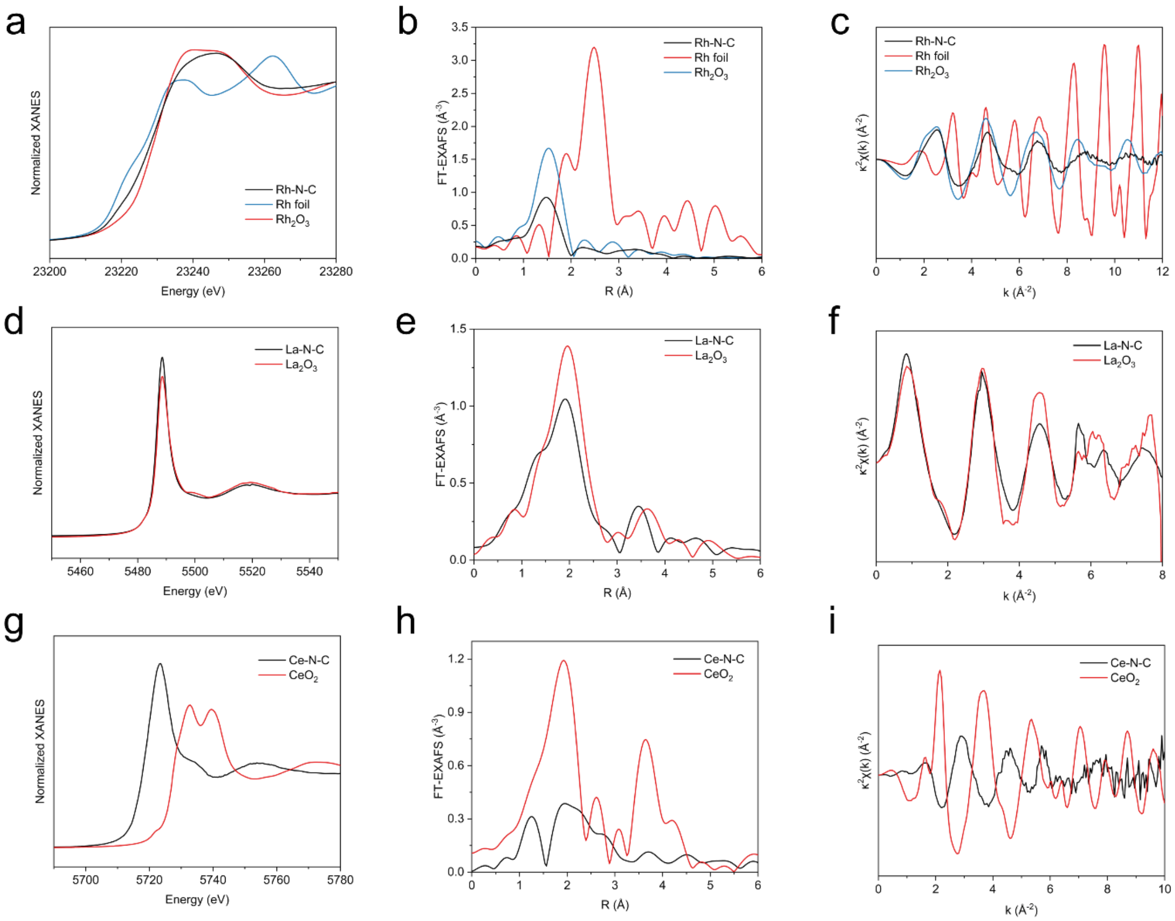

Figure S 12. XANES, FT-EXAFS and EXAFS spectra for *4d* and *f*-metals (a-c) Rh-K edge in Rh-N-C, (d-f) La-L edge in La-N-C and (g-i) Ce-L edge in Ce-N-C. See Supplementary note 1 for a full discussion on the XANES interpretation and determination of metal oxidation states and Supplementary Table 2 and Supplementary note 3 for a full discussion on the EXAFS analysis.

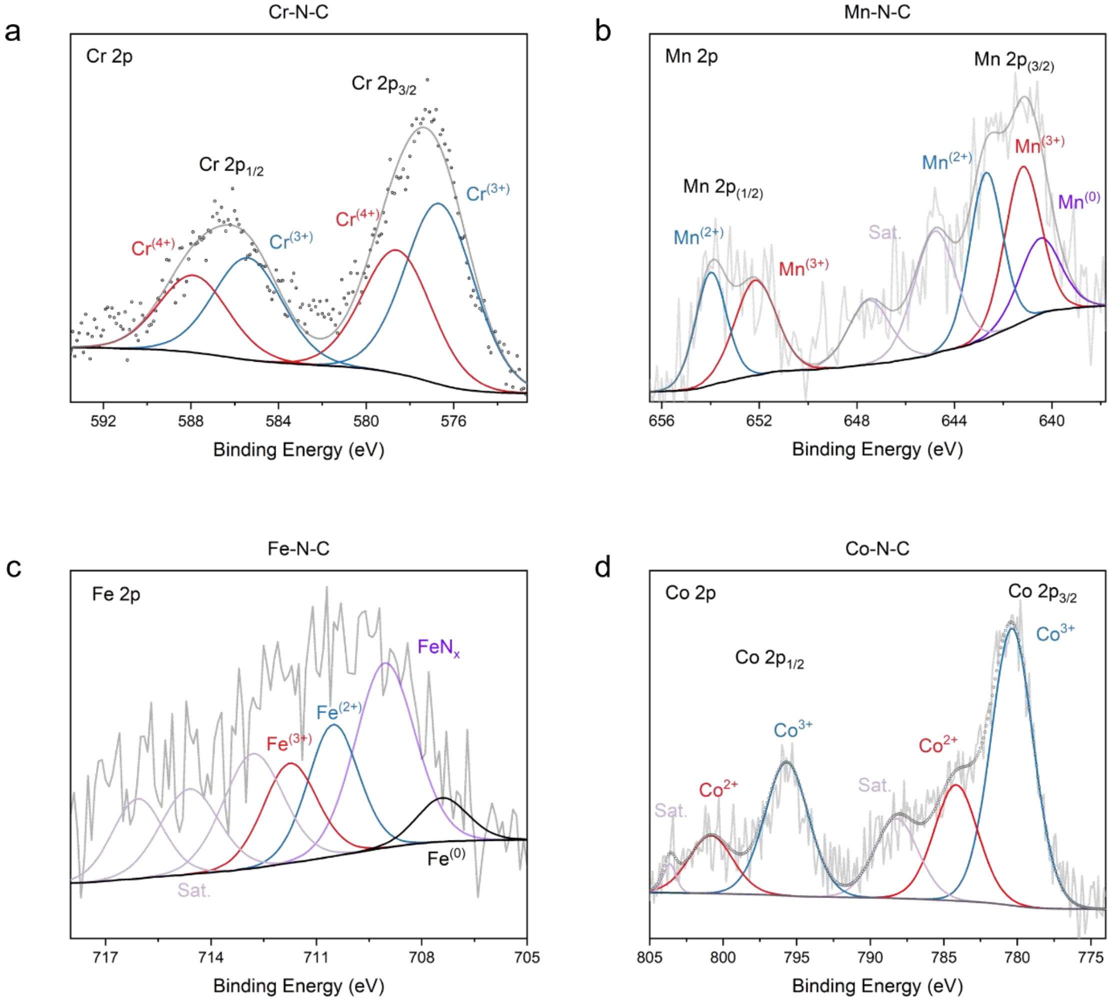

Figure S 13. Deconvoluted XPS spectra for 3d metals (a) Cr-N-C, Cr 2p spectra showing a mixed oxidation state of Cr^{3+} and Cr^{4+}. (b) Mn-N-C, Mn 2p spectra showing a mix between Mn^{0} and Mn^{3+}. (c) Fe-N-C, Fe 2p spectra showing a mixed metal oxidation state between Fe^{0} and Fe^{3+}. (d) Co-N-C, Co 2p spectra showing a mixed metal oxidation state between Co^{2+} and Co^{3+}.

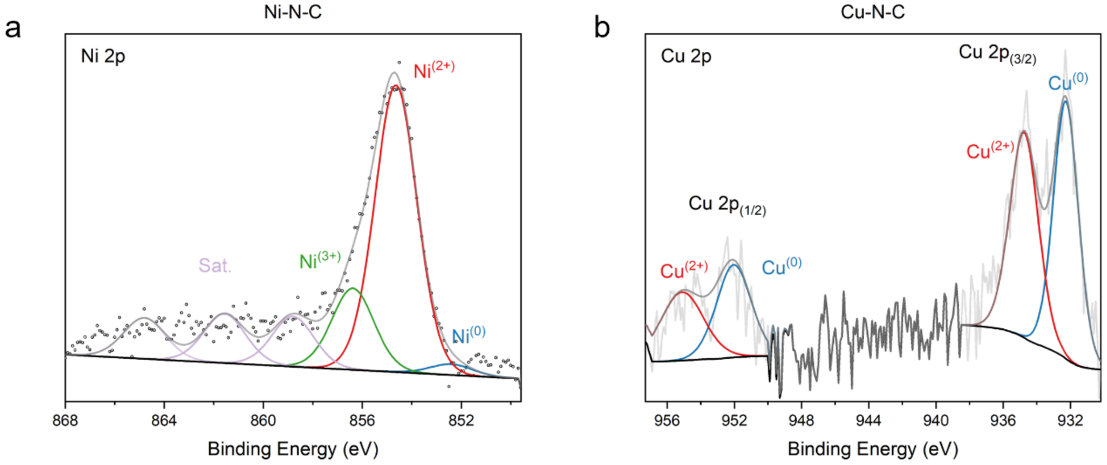

Figure S 14. Deconvoluted XPS spectra for 3d metals (a) Ni-N-C, Ni 2p spectra showing a metal oxidation state between $Ni^{(0)}$ and Ni^{3+}. (b) Cu-N-C, Cu 2p spectra showing a metal oxidation state between $Cu^{(0)}$ and $Cu^{(2+)}$.

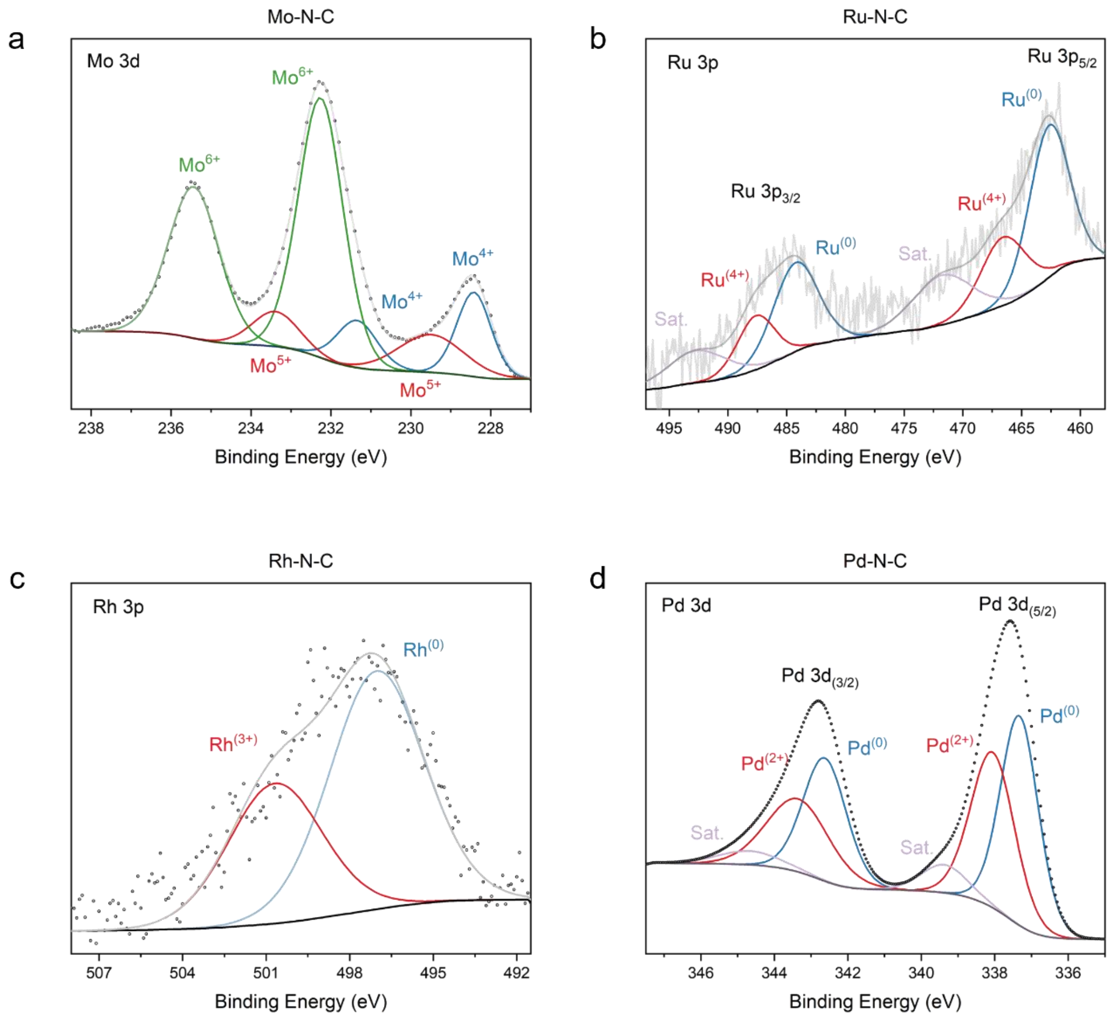

Figure S 15. Deconvoluted XPS spectra for 4 d metals (a) Mo-N-C, Mo 3d spectra showing a metal oxidation state between Mo^{4+} and Mo^{6+}. (b) Ru-N-C, Ru 3p spectra showing a metal oxidation state between Ru^{0} and Ru^{4+}. (c) Rh-N-C, Rh 3p spectra showing a metal oxidation state between $Rh^{(0)}$ and $Rh^{(3+)}$. (d) Pd-N-C, Pd 3d spectra showing a metal oxidation state between $Pd^{(0)}$ and $Pd^{(2+)}$.

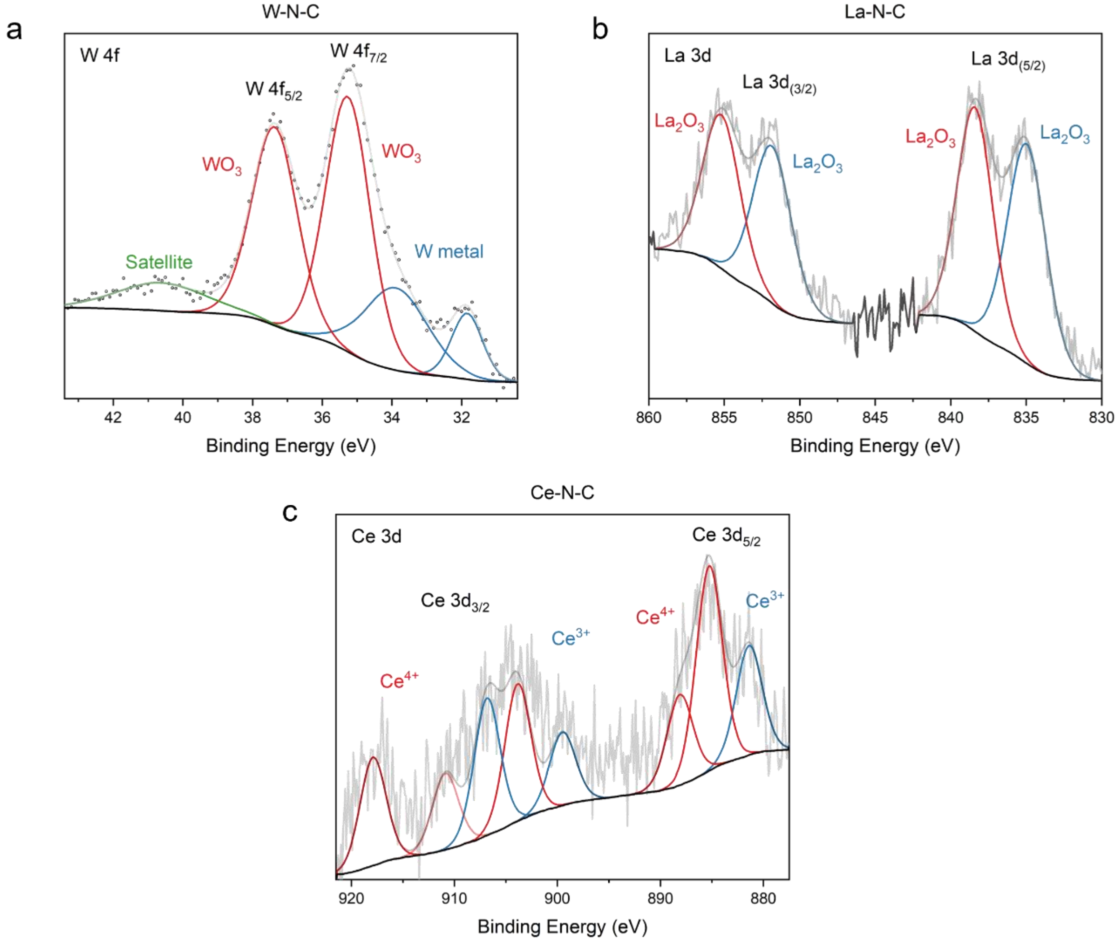

Figure S 16. Deconvoluted XPS spectra for 5 d and f metals (a) W-N-C, W 4f spectra showing a metal oxidation state between $W^{(0)}$ and $W^{(6+)}$. (b) La-N-C, La 3d spectra showing a metal oxidation state of $La^{(3+)}$. (c) Ce-N-C, Ce 3d spectra showing a metal oxidation state between $Ce^{(3+)}$ and $Ce^{(4+)}$.

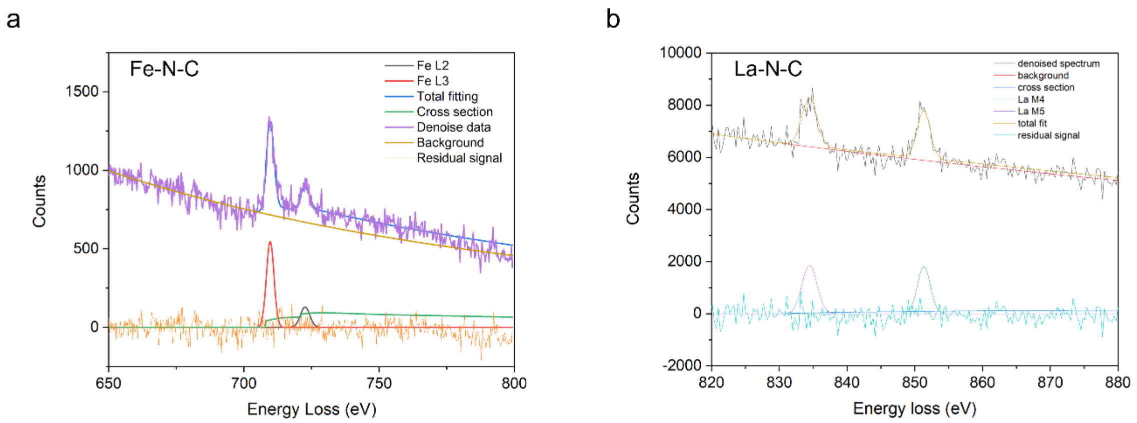

Figure S 17. EELS valence state analysis. a) Fe-N-C, the Fe L_3/L_2 intensity ratio is 4.2, $L_2 – L_3$ separation distance is 12.9 eV, according to the literature this suggests a valance state of Fe close to Fe^{2+}. b) La-N-C, the La M_5/M_4 intensity ratio is 0.97, being close to a standard La_2O_3 sample, suggesting a La valence state close to La^{3+}.

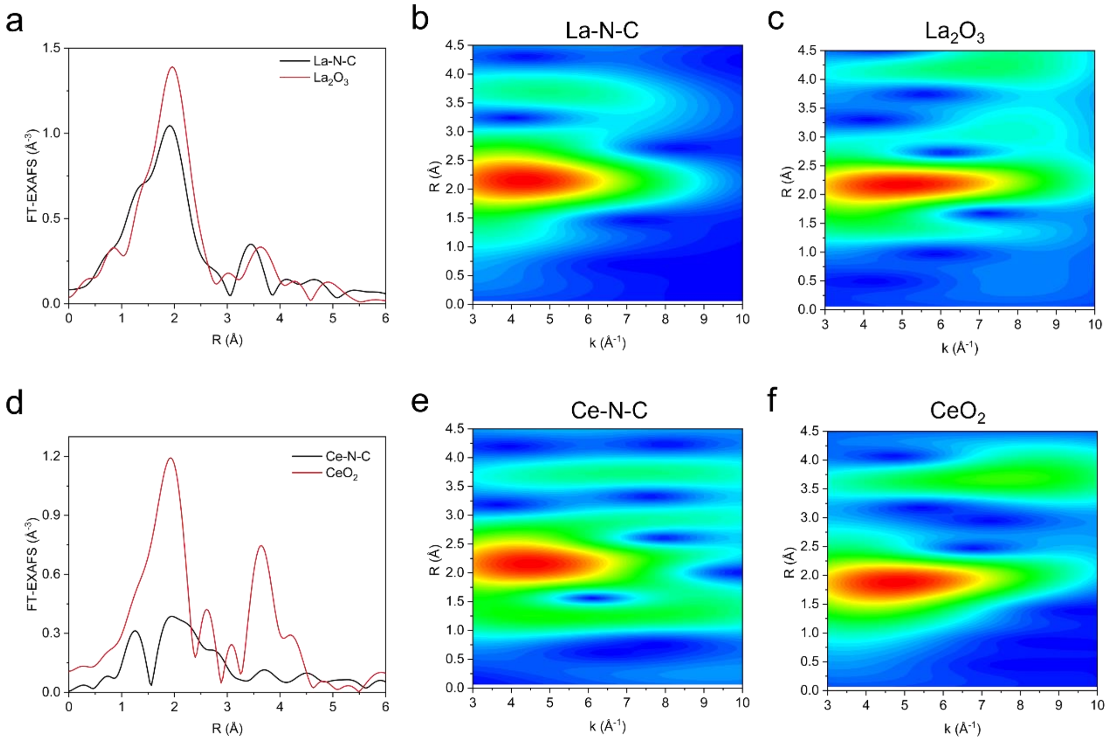

Figure S 18. FT-EXAFS and wavelet transformation (WT) for *f*-metals (a-c) La-N-C and the corresponding La_2O_3 standard and (d-f) Ce-N-C and the corresponding CeO_2 standard. In the WT-EXAFS, in addition to the peak intensity observed at *ca.* 2.1 Å at low k-values, an additional peak is present at *ca.* 3.7 Å again at low k-values, indicating interactions with only low Z-number neighbors, in particular La-C. For Ce-N-C, the WT-EXAFS shows two features located at *ca.* 1.5 Å and 2 Å, both located at low k-values (*ca.* 4-5 $Å^{-1}$), matching the Ce-O intensity in the CeO_2 WT-EXAFS, indicating the split peaks for Ce-N-C constitute interactions between Ce and low Z number neighbors (C, N or O atoms). While an additional feature at 3.7 Å is also observed at higher k-number (7 $Å^{-1}$), suggesting that in addition to atomically dispersed Ce-N_x sites, the formation of small oxide clusters can coexist.

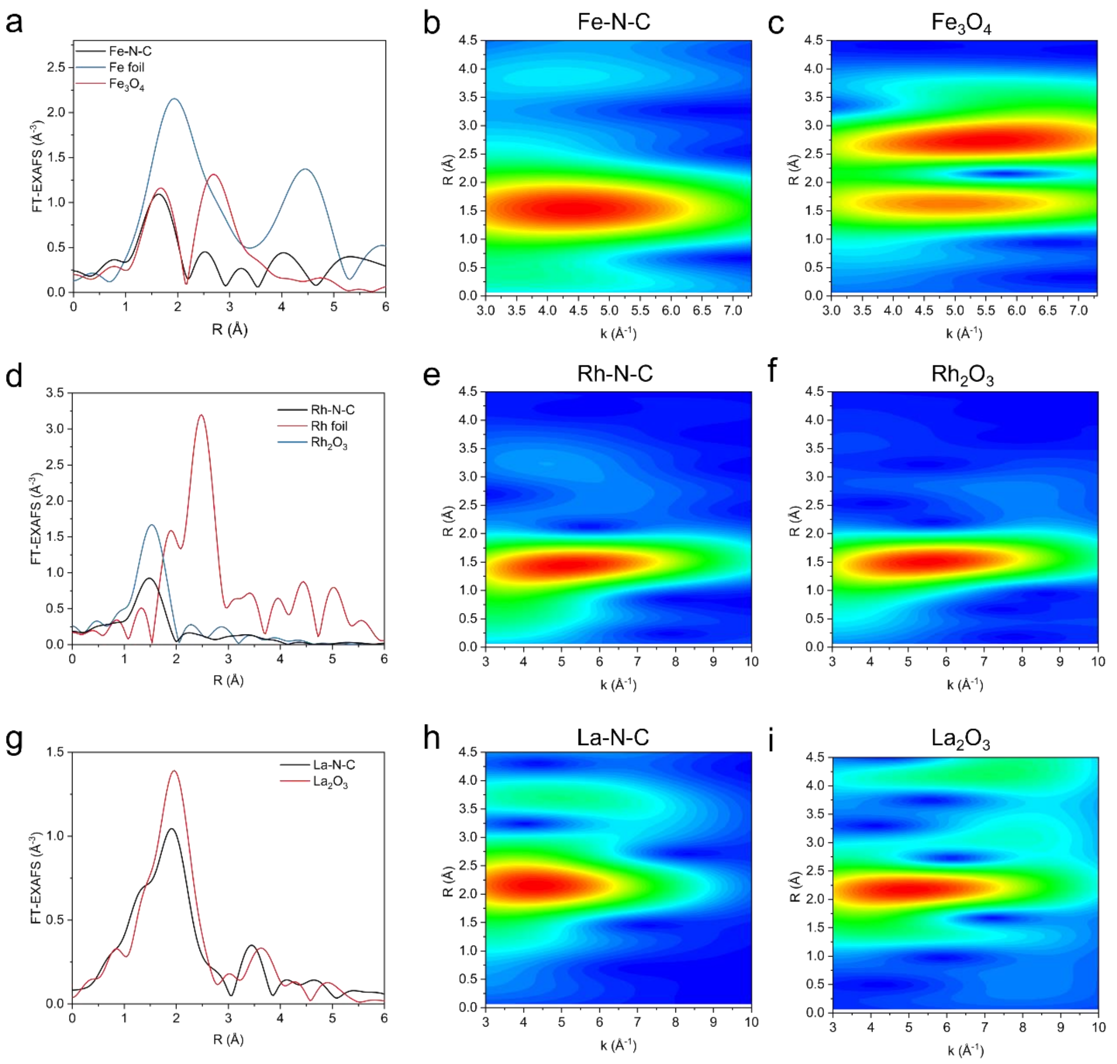

Figure S 19. FT-EXAFS and WT-EXAFS for (a-c) Fe-N-C and the corresponding Fe_3O_4 standard, (d-f) Rh-N-C and the corresponding Rh_2O_3 standard (g-i) La-N-C with the corresponding La_2O_3 standard.

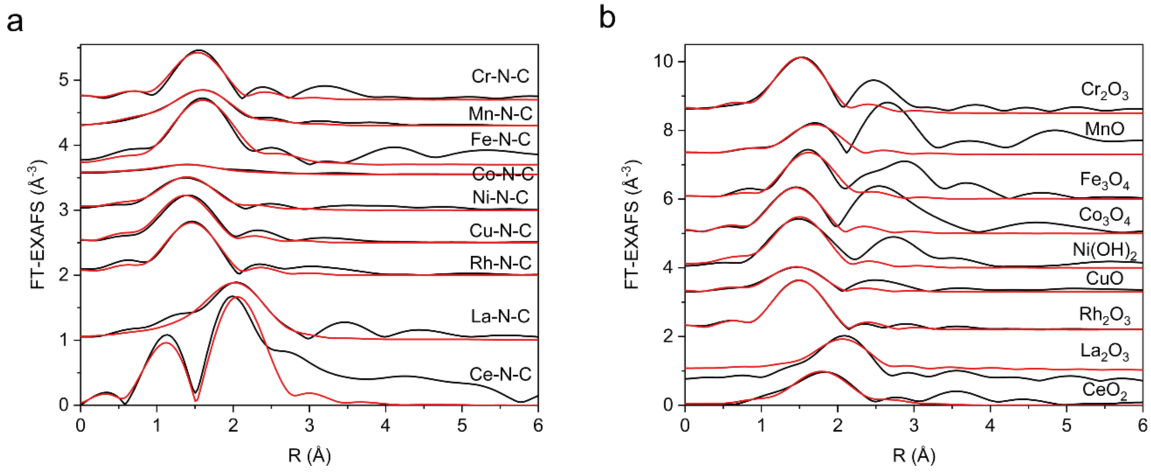

Figure S 20. Fitting of the Fourier-transformed experimental (black lines) and fitted (red lines) (a) EXAFS spectra for the M-N-C catalysts and (b) corresponding reference materials, where only contributions from the first coordination shell were considered. Note that for Ce-N-C two different paths were included in the fit, to describe the split distributions of bond-lengths to neighboring atoms due to the displacement of Ce from the center of Ce-N_4 unit.

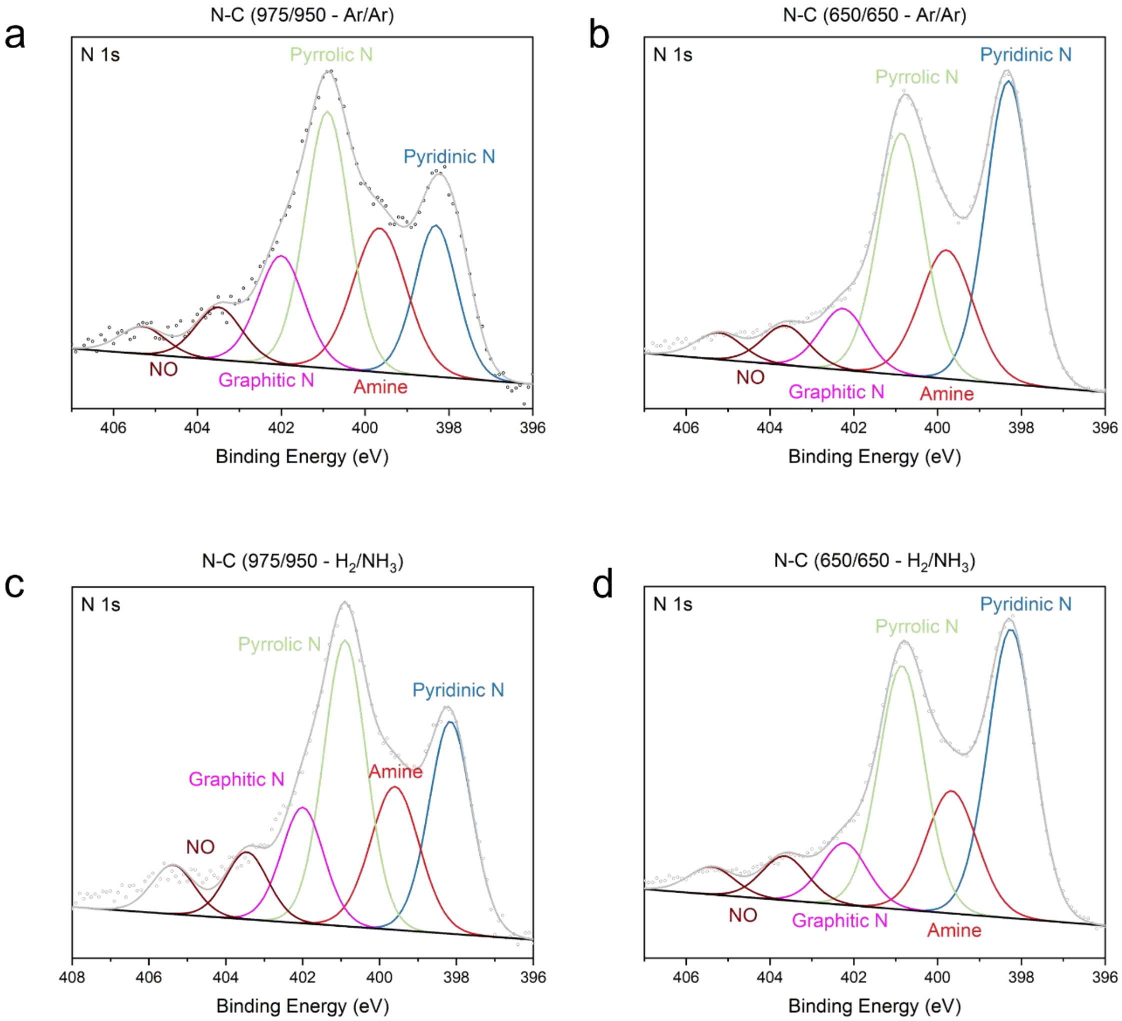

Figure S 21. Deconvoluted N 1*s* XPS spectra for the metal free N-C synthesized under varying conditions. a) N-C (975-Ar / 950-Ar), b) N-C (650-Ar / 650-Ar), c) N-C (975-H_2 / 950-NH_3) and d) N-C (650- H_2 / 650- NH_3).

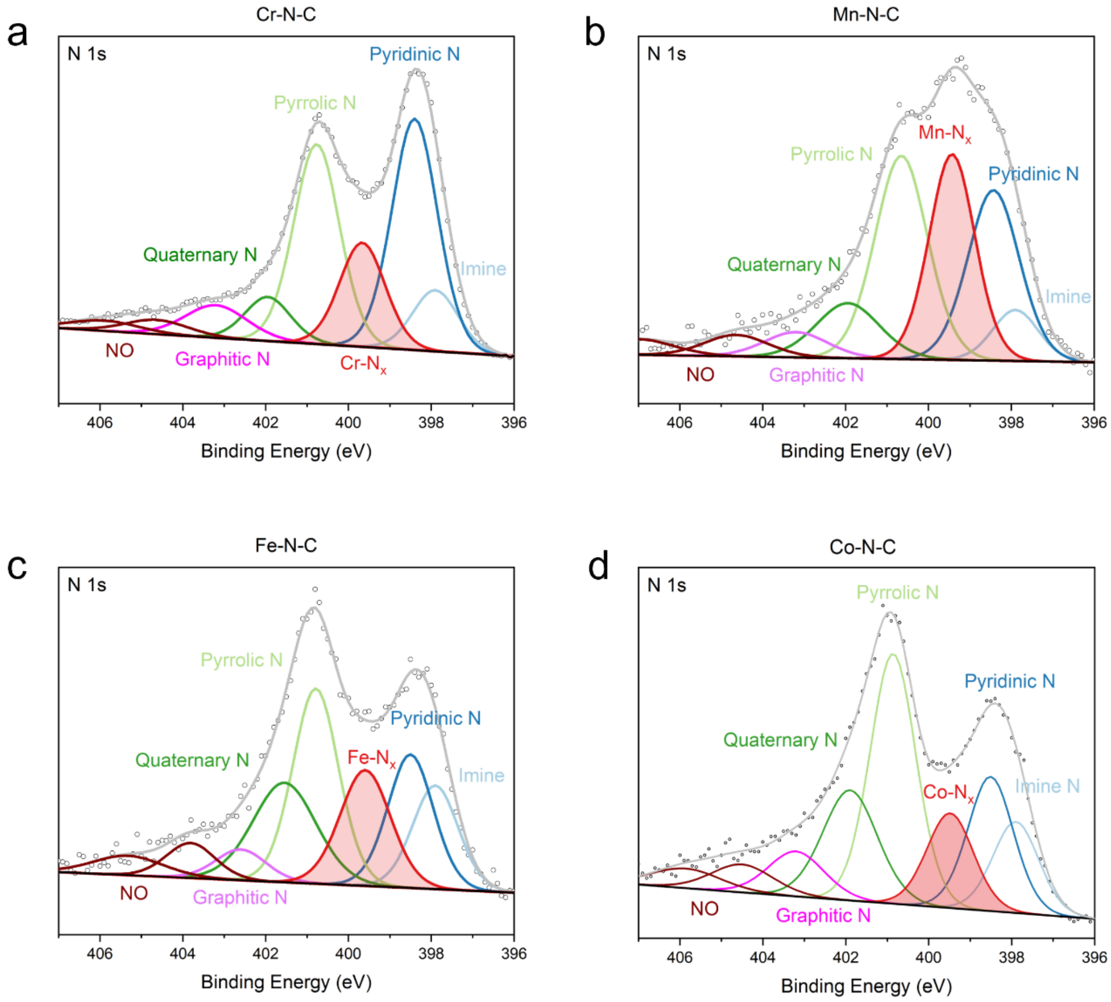

Figure S 22. Deconvoluted N 1*s* XPS spectra for the 3d metal; (a) Cr-N-C, (b) Mn-N-C , (c) Fe-N-C and (d) Co-N-C catalysts.

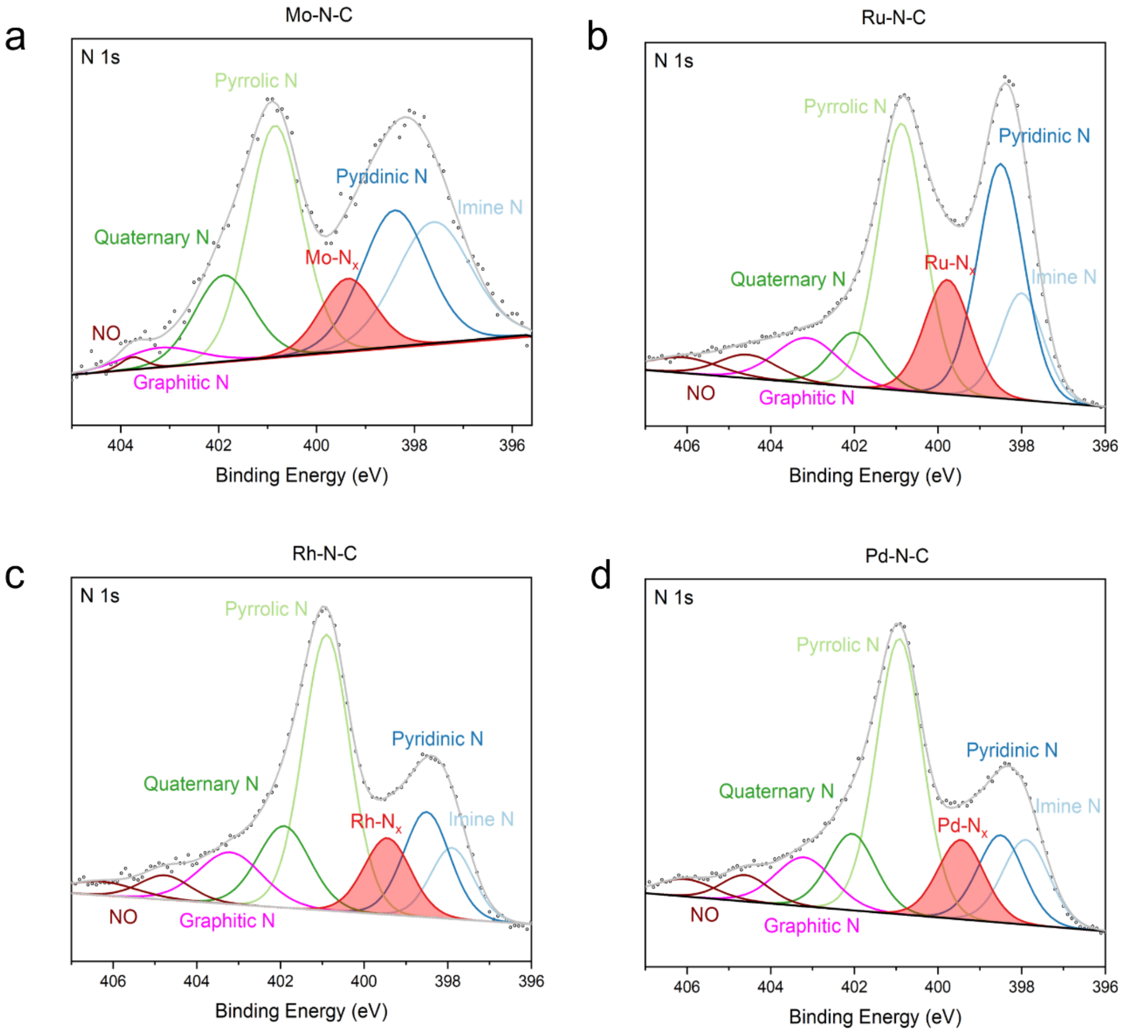

Figure S 23. Deconvoluted N 1*s* XPS spectra for the 4d metal; (a) Mo-N-C, (b) Ru-N-C, (c) Rh-N-C and (d) Pd-N-C catalysts.

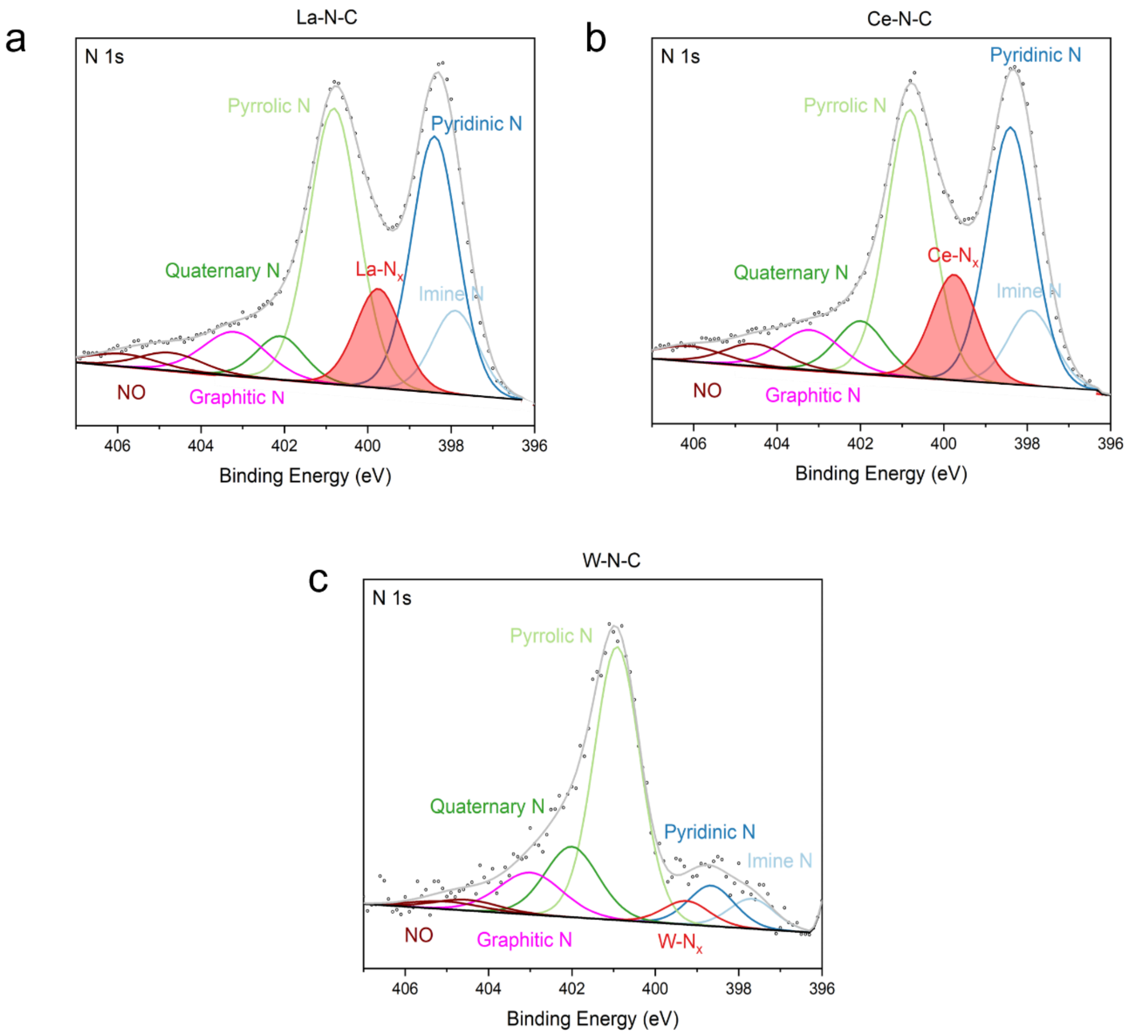

Figure S 24. Deconvoluted N 1*s* XPS spectra for the *f* and 5*d* metal; (a) La-N-C, (b) Ce-N-C and (c) W-N-C catalysts.

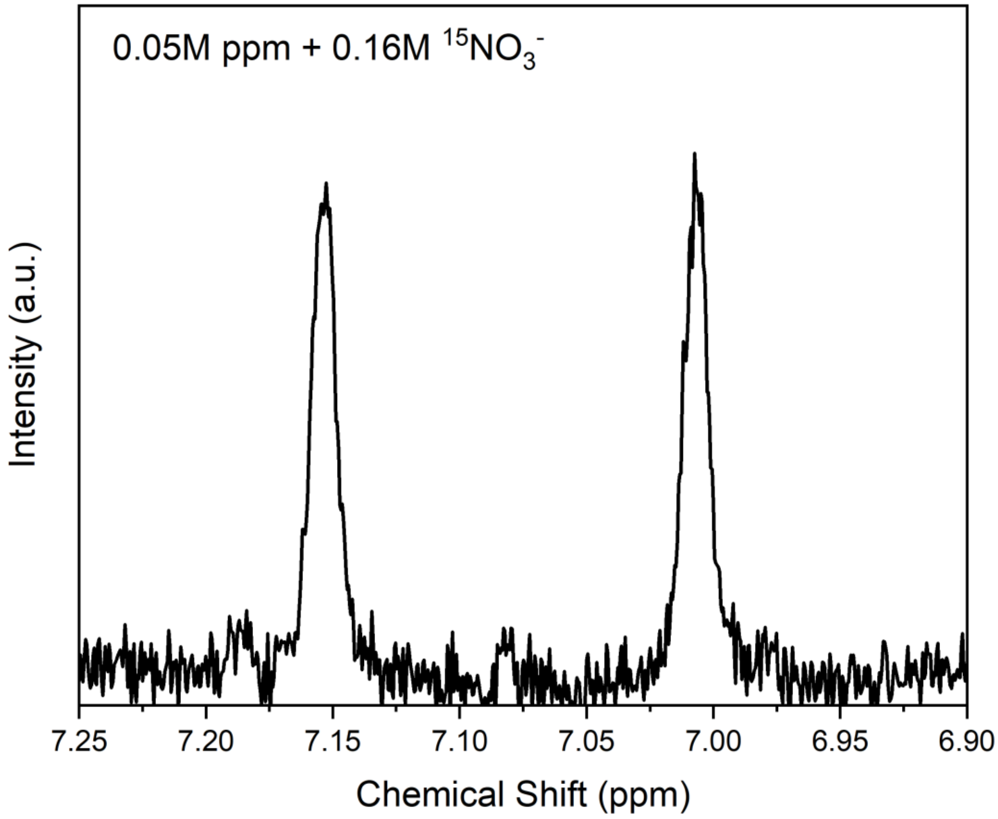

Figure S 25. Isotopically labeled NO_3RR with 0.05M PBS + 0.16M $K^{15}NO_3^-$ electrolyte for metal-free N-C catalyst. Electrolysis is performed for 4 hours at -0.40 V *vs.* RHE to ensure a reliable amount of $^{15}NH_3$ had been produced. The typical doublet is observed for $^{15}NH_3$. The FE for $^{15}NH_3$ is 36% with a $^{15}NH_3$ yield rate of 1.14 µmol h^{-1} cm^{-2}, agreeing with the non-isotopic NO_3RR for N-C (975-H_2 / 950-NH_3) with a FE of 40% and a yield rate of 1.32 µmol h^{-1} cm^{-2}. This confirms both that the metal free N-C is active for the NO_3RR and that the NH_3 produced originates from the nitrate salt rather than catalyst degradation from the N-C support or other contamination.

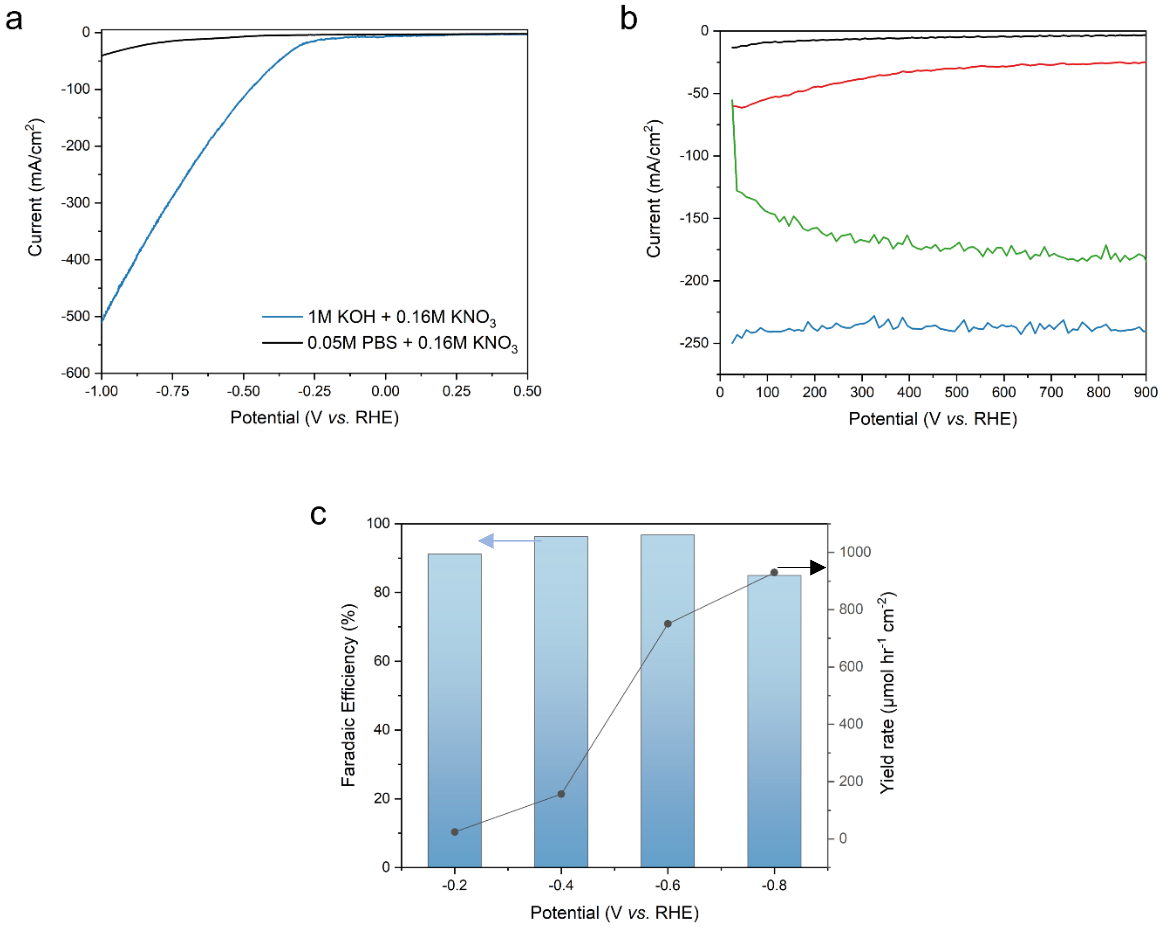

Figure S 26. NO_3RR electrolysis for the Fe-N-C catalyst in a static cell with alkaline electrolyte (1M KOH, pH = 14). a) LSV comparing 0.05M PBS (pH = 7) with 0.16M KNO_3 and 1M KOH (pH = 14) with 0.16M KNO_3. b) Chronoamperometry in 1M KOH + 0.16M KNO_3 electrolyte at -0.2 to -0.8 V *vs.* RHE. c) Faradaic efficiency for NH_3 (left) and NH_3 yield rate per geometric surface area (right) as a function of applied potential.

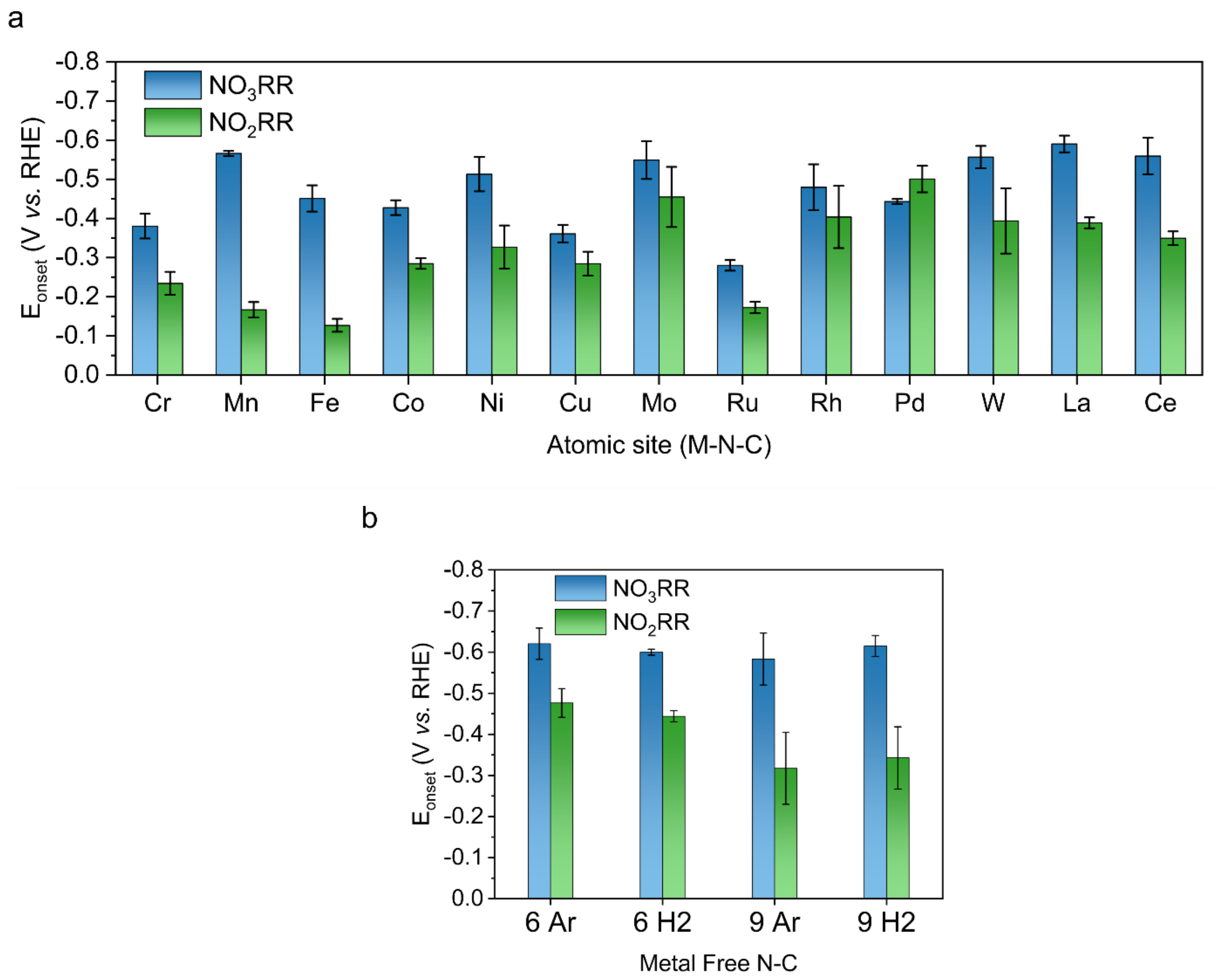

Figure S 27. Onset potential of the (a) M-N-C and (b) metal-free N-C catalysts for the NO_2RR and NO_3RR. The onset potentials were determined at a current density of -1.0 mA/cm^2. Cr- and Cu-N-C exhibit the lowest onset potentials for the NO_3RR, while Fe-N-C demonstrates the lowest onset potential for the NO_2RR. Note that Ru-N-C exhibits an early onset potential for both the NO_3 and NO_2RR, however, the NO_3 and NO_2RR electrolysis results in Figure 3d and 4d suggest activity is largely attributed to the HER. Figure b suggest that over the metal free N-C catalysts the onset potential for the NO_3RR not impacted by the varying pyrolysis conditions, while for the NO_2RR, a slightly earlier onset potential is shown for the NO_2RR.

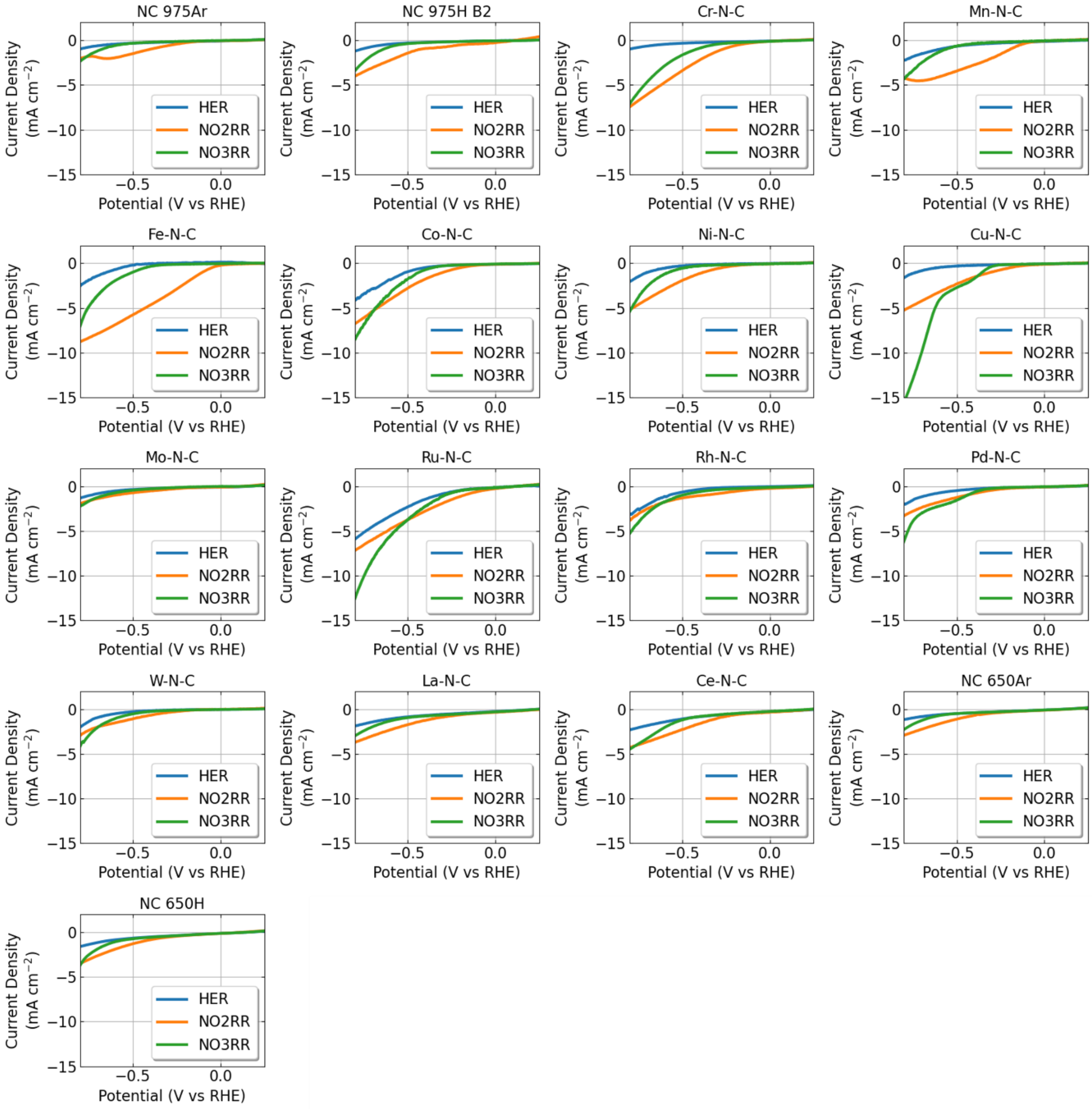

Figure S 28. Linear sweep voltammetry in 0.05M PBS (HER), 0.01M KNO_2 (NO_2RR) and 0.16M KNO_3 (NO_3RR) for all metal free N-C and M-N-C catalysts.

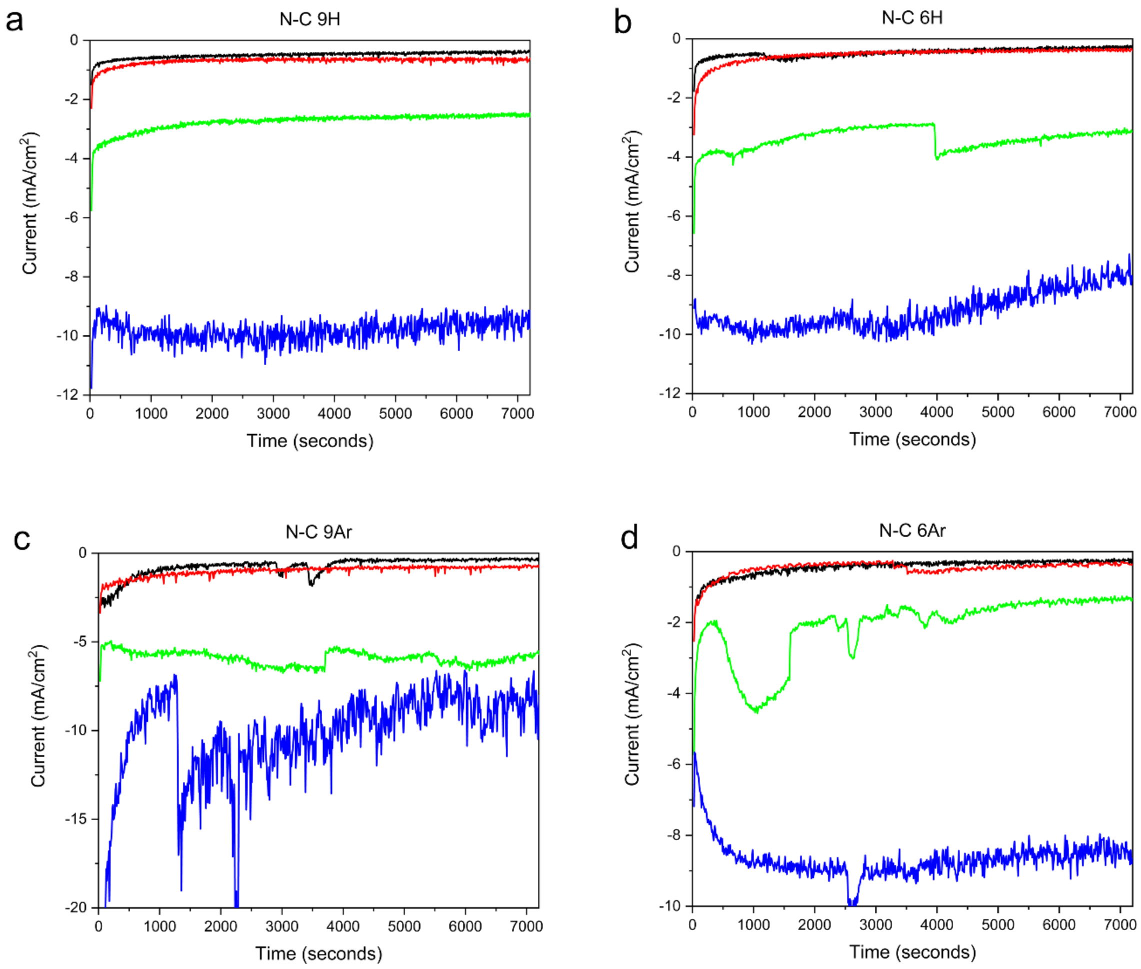

Figure S 29. NO_3RR chronoamperometry on (a) N-C (975 H_2 / 950 Ar), (b) N-C (650 H_2 / 650 Ar), (c) N-C (975 Ar / 950 Ar) and (d) N-C (650 Ar / 650 Ar) catalysts. NO_3RR electrolysis is performed for 2 hours in a 0.05M PBS + 0.16M NO_3^- electrolyte. The black, red, green and blue curves correspond to applied potentials of -0.20, -0.40, -0.60 and -0.80 V *vs.* RHE, respectively.

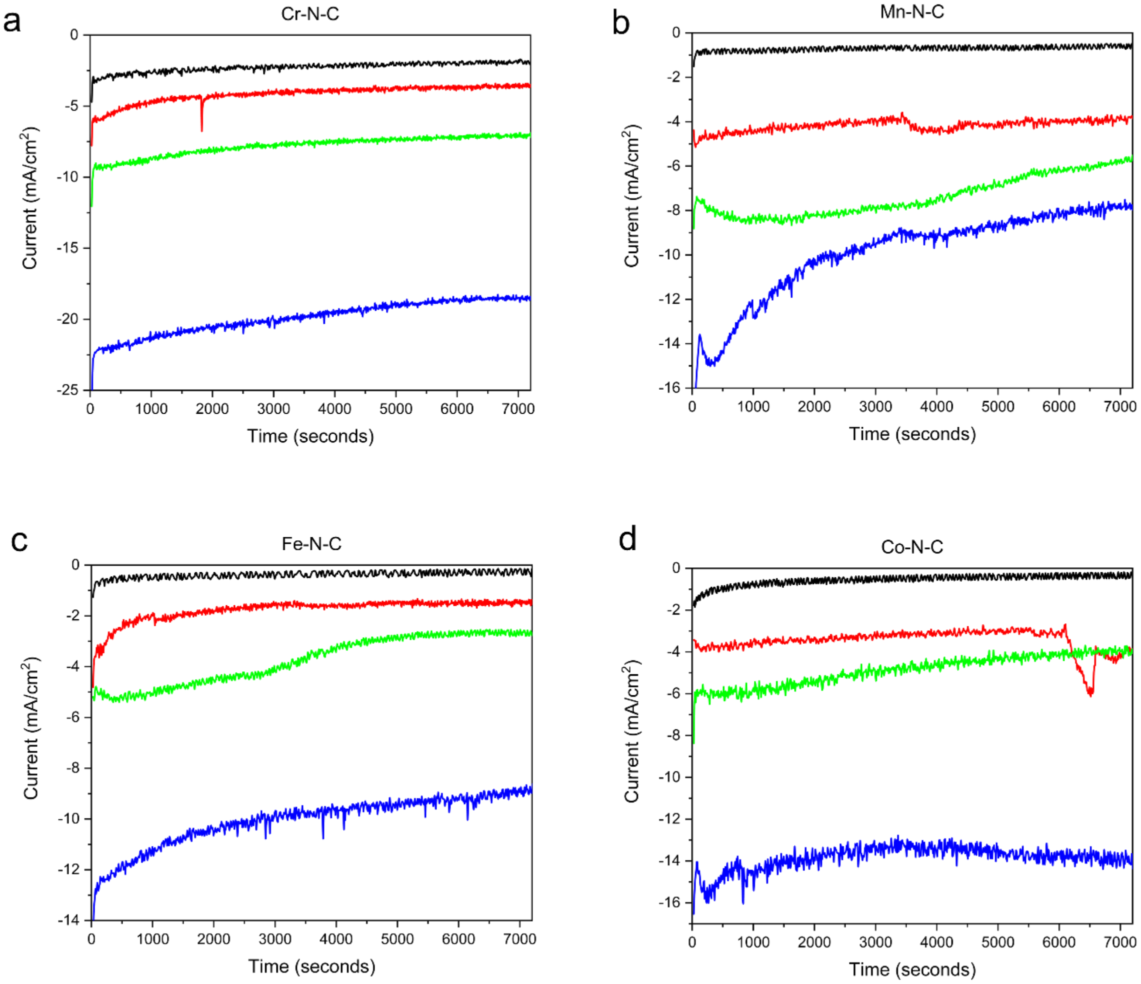

Figure S 30. NO_3RR chronoamperometry on (a) Cr-N-C, (b) Mn-N-C, (c) Fe-N-C and (d) Co-N-C catalysts. NO_3RR electrolysis is performed for 2 hours in a 0.05M PBS + 0.16M NO_3^- electrolyte. The black, red, green and blue curves correspond to applied potentials of -0.20, -0.40, -0.60 and -0.80 V *vs.* RHE, respectively.

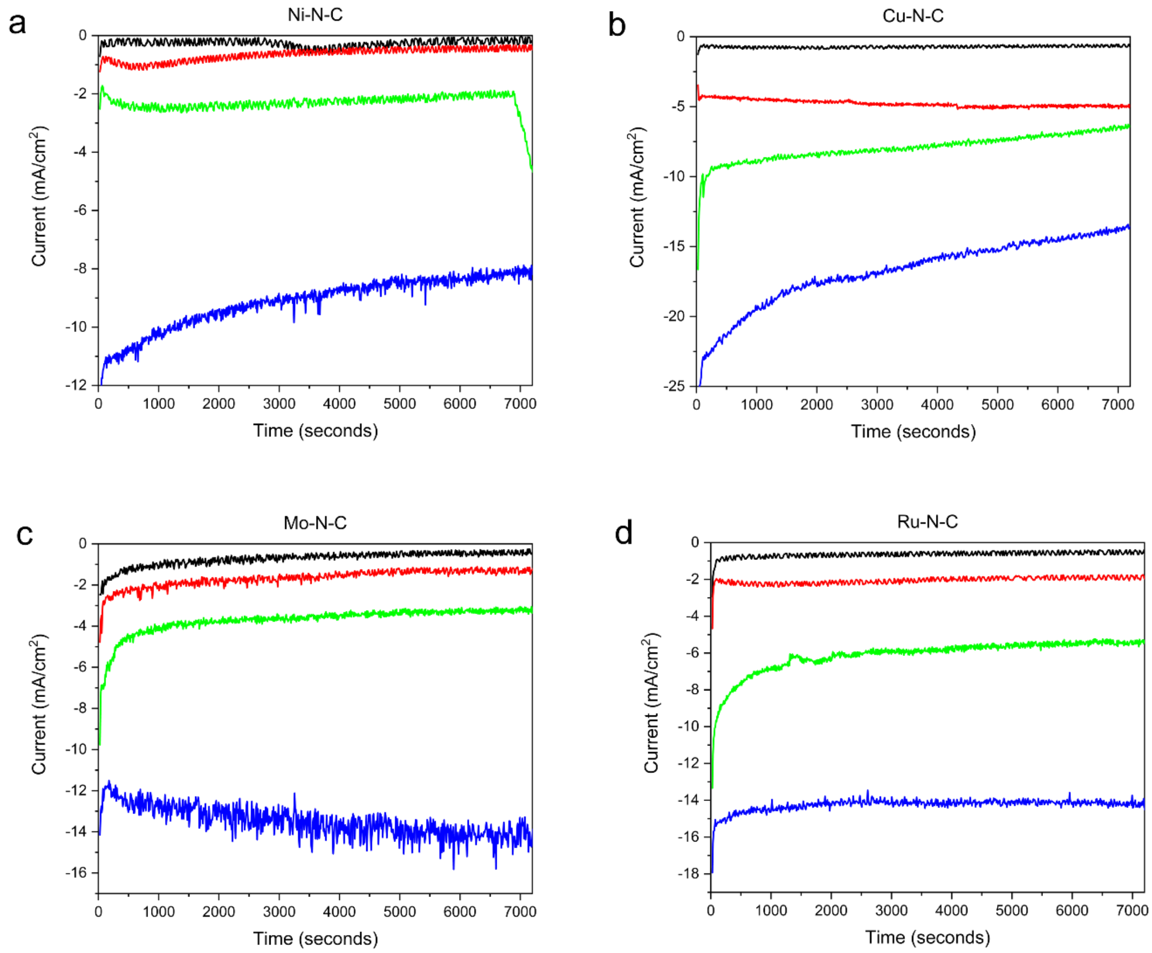

Figure S 31. NO_3RR chronoamperometry on (a) Ni-N-C, (b) Cu-N-C, (c) Mo-N-C and (d) Ru-N-C catalysts. NO_3RR electrolysis is performed for 2 hours in a 0.05M PBS + 0.16M NO_3^- electrolyte. The black, red, green and blue curves correspond to applied potentials of -0.20, -0.40, -0.60 and -0.80 V *vs.* RHE, respectively.

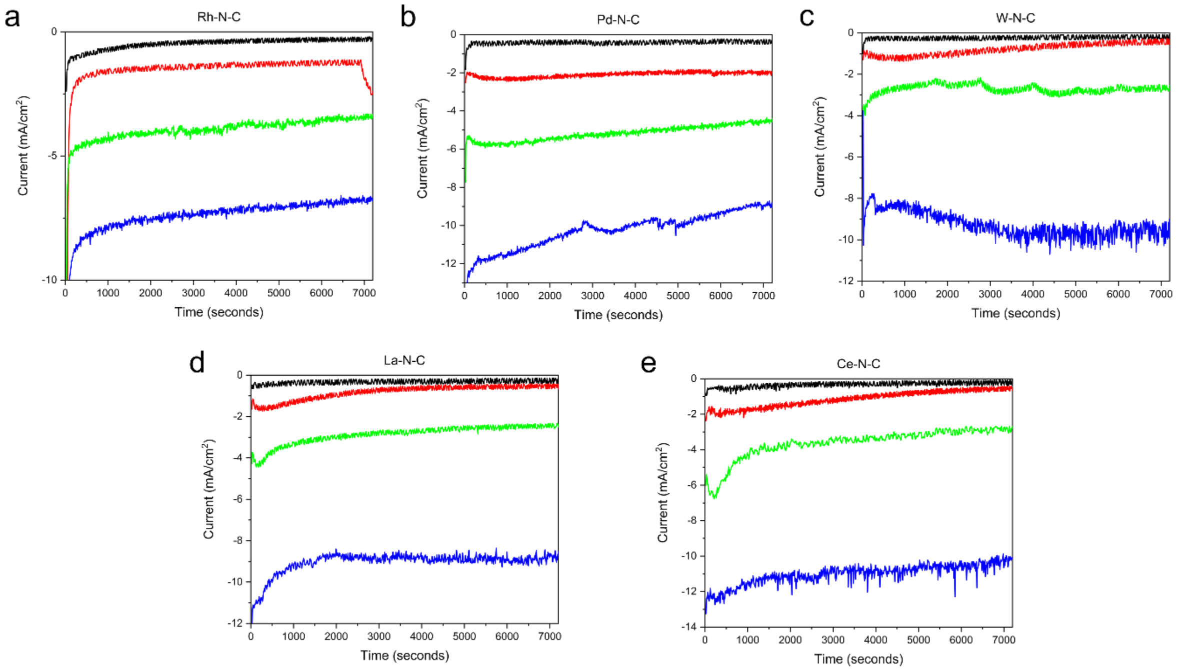

Figure S 32. NO_3RR chronoamperometry on (a) Rh-N-C, (b) Pd-N-C, (c) W-N-C, (d) La-N-C and (e) Ce-N-C catalysts. NO_3RR electrolysis is performed for 2 hours in a 0.05M PBS + 0.16M NO_3^- electrolyte. The black, red, green and blue curves correspond to applied potentials of -0.20, -0.40, -0.60 and -0.80 V *vs.* RHE, respectively.

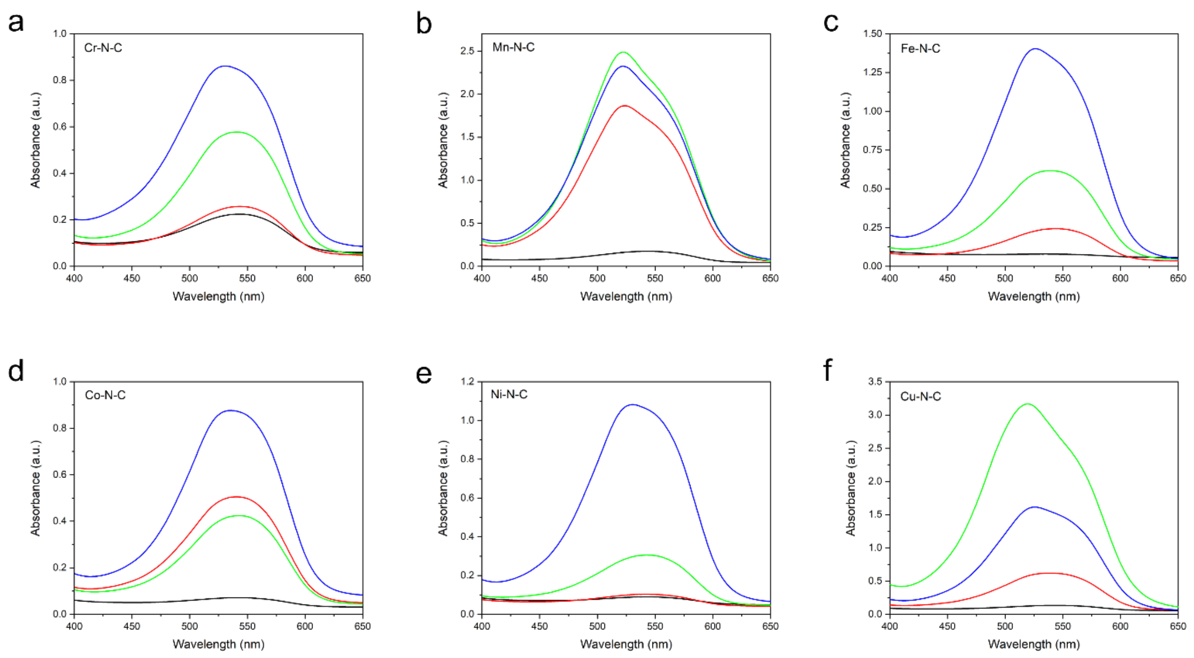

Figure S 33. NO_3RR UV Vis detection of NO_2^- for (a) Cr-N-C and (b) Mn-N-C (c) Fe-N-C, (d) Co-N-C, (e) Ni-N-C and (f) Cu-N-C for 2 hr electrolysis at an applied potential of -0.20 V (black), -0.40 V (red), -0.60 V (green) and -0.80 V *vs.* RHE (blue). Dashed curves are spectra from electrolyte in the counter chamber. Dilutions have been applied to obtain a NH_3 concentration in the calibration range.

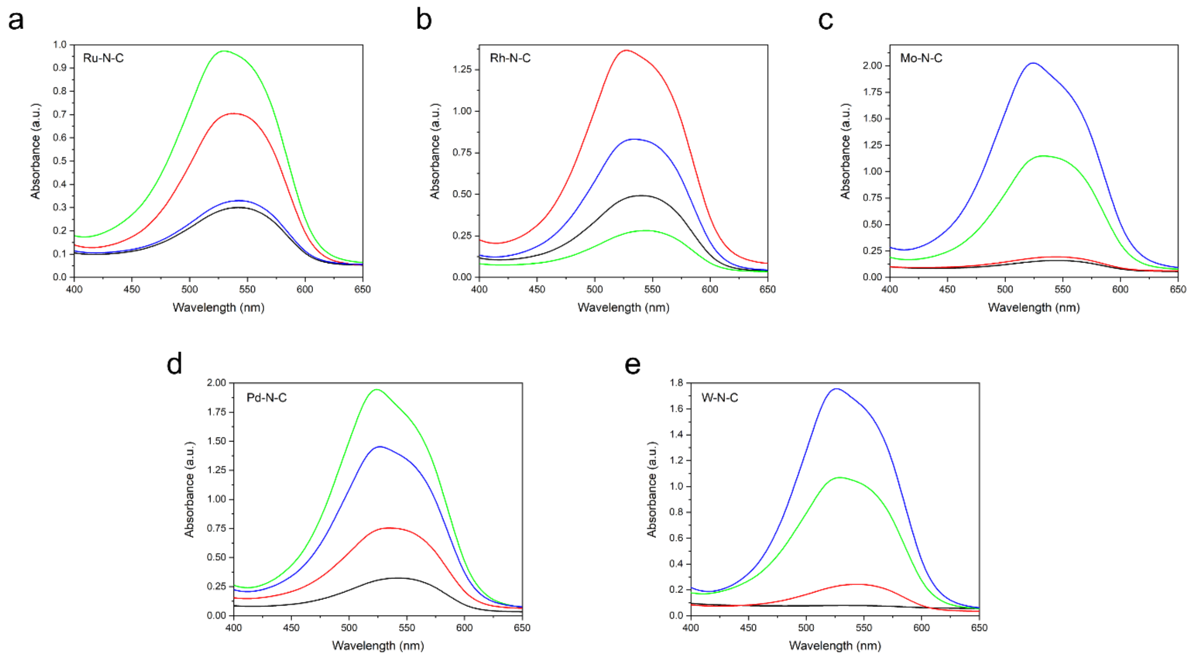

Figure S 34. NO_3RR UV Vis detection of NO_2^- for (a) Mo-N-C and (b) Ru-N-C (c) Rh-N-C, (d) Pd-N-C and (e) W-N-C for 2 hr electrolysis at an applied potential of -0.20 V (black), -0.40 V (red), -0.60 V (green) and -0.80 V *vs.* RHE (blue). Dashed curves are spectra from electrolyte in the counter chamber. Dilutions have been applied to obtain a NH_3 concentration in the calibration range.

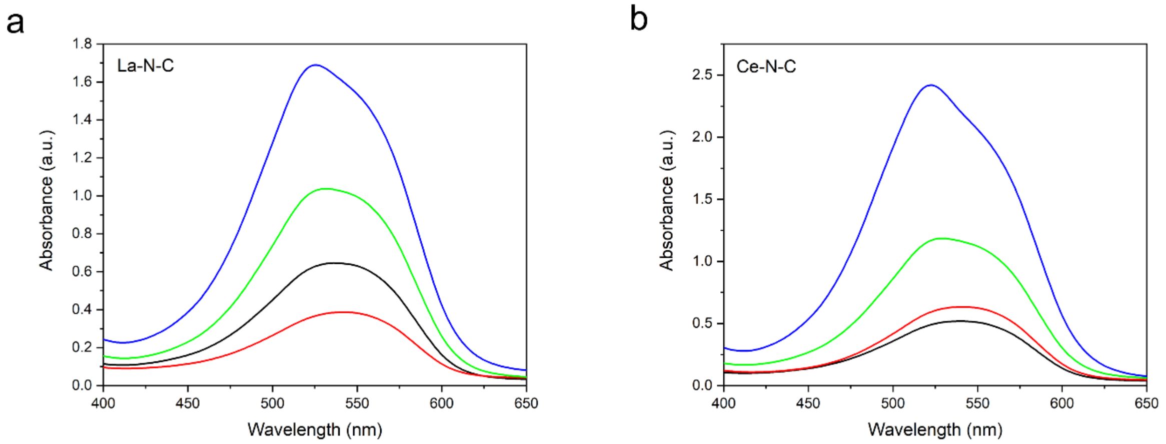

Figure S 35. NO_3RR UV Vis detection of NO_2^- for (a) La-N-C and (b) Ce-N-C for 2 hr electrolysis at an applied potential of -0.20 V (black), -0.40 V (red), -0.60 V (green) and -0.80 V *vs.* RHE (blue). Dashed curves are spectra from electrolyte in the counter chamber. Dilutions have been applied to obtain a NH_3 concentration in the calibration range.

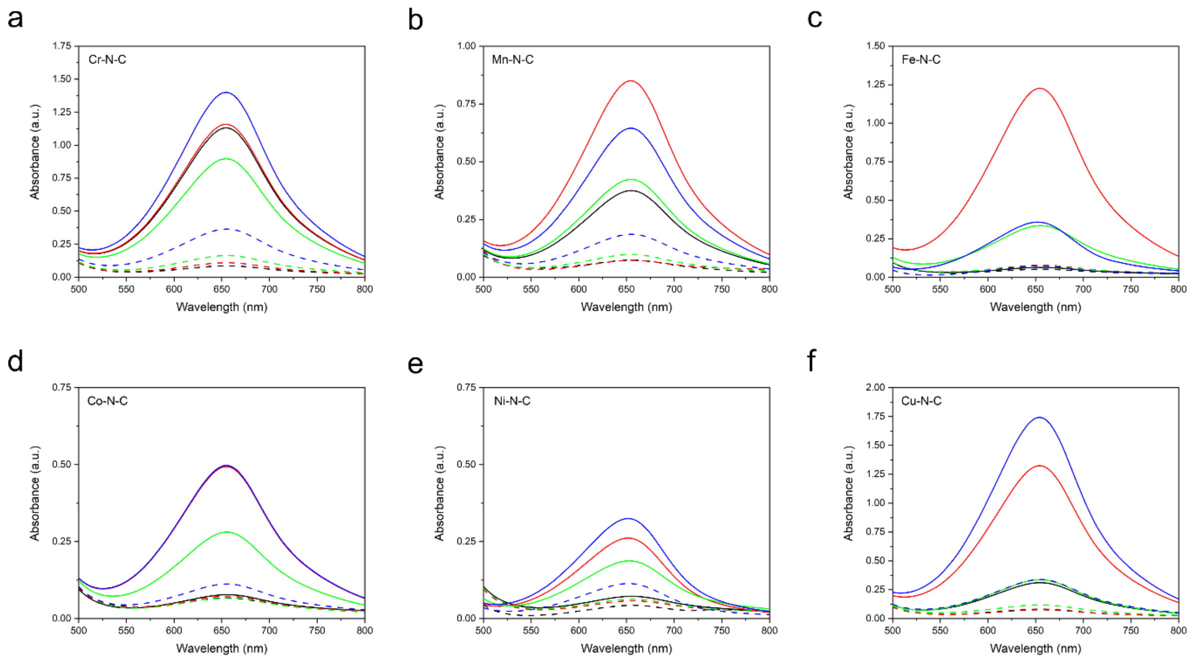

Figure S 36. NO_3RR UV Vis detection of NH_3 for (a) Cr-N-C and (b) Mn-N-C (c) Fe-N-C, (d) Co-N-C, (e) Ni-N-C and (f) Cu-N-C for 2 hr electrolysis at an applied potential of -0.20 V (black), -0.40 V (red), -0.60 V (green) and -0.80 V *vs.* RHE (blue). Dashed curves are spectra from electrolyte in the counter chamber. Dilutions have been applied to obtain a NH_3 concentration in the calibration range.

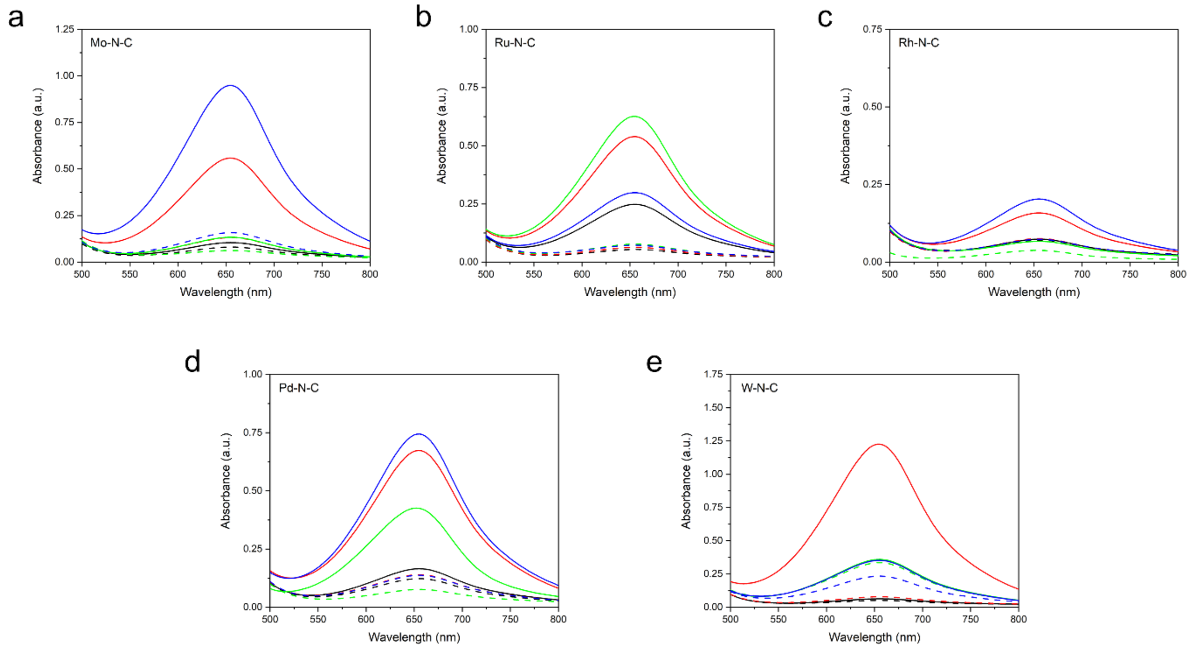

Figure S 37. NO_3RR UV Vis detection of NH_3 for (a) Mo-N-C and (b) Ru-N-C (c) Rh-N-C, (d) Pd-N-C and (e) W-N-C for 2 hr electrolysis at an applied potential of -0.20 V (black), -0.40 V (red), -0.60 V (green) and -0.80 V *vs.* RHE (blue). Dashed curves are spectra from electrolyte in the counter chamber. Dilutions have been applied to obtain a NH_3 concentration in the calibration range.

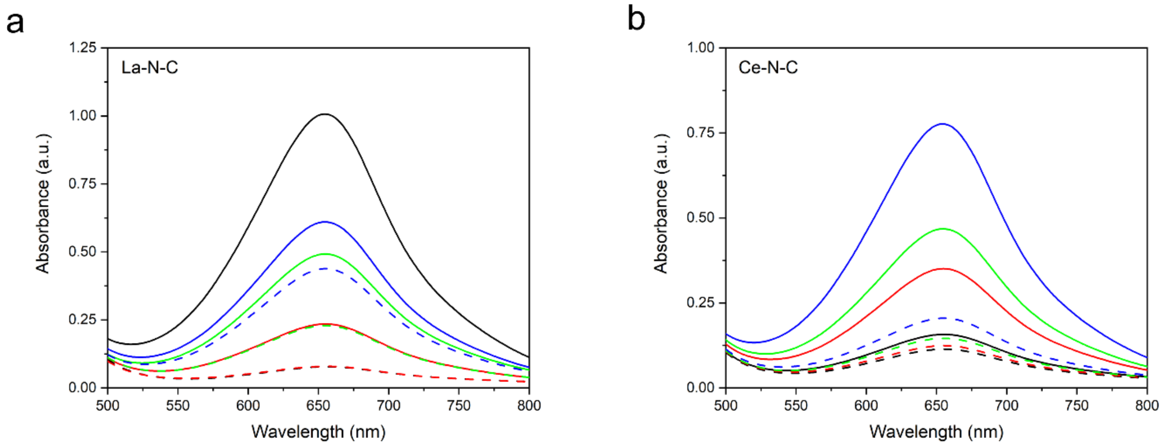

Figure S 38. NO_3RR UV Vis detection of NH_3 for (a) La-N-C and (b) Ce-N-C for 2 hr electrolysis at an applied potential of -0.20 V (black), -0.40 V (red), -0.60 V (green) and -0.80 V *vs.* RHE (blue). Dashed curves are spectra from electrolyte in the counter chamber. Dilutions have been applied to obtain a NH_3 concentration in the calibration range.

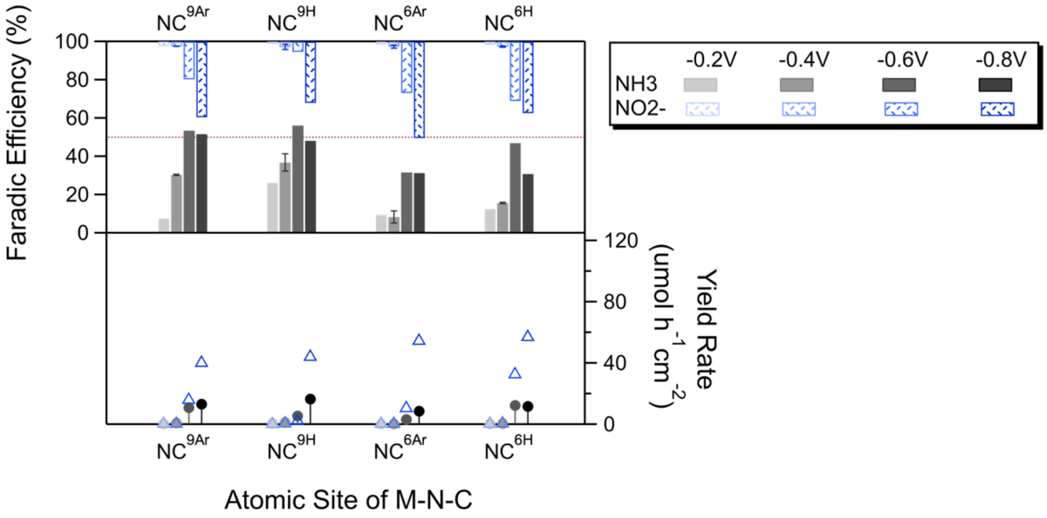

Figure S39. Electrochemical NO_3RR for metal free N-C catalysts in 0.05M PBS + 0.16M KNO_3 for 2 hours at an applied potential of -0.20 to -0.80 V *vs.* RHE. The top section of the figure shows the Faradic efficiency for NO_2^- (blue; top-down) and NH_3 (gray; bottom-up) A red line is set an efficiency of 50% to guide the eye. The bottom section of the figure shows the corresponding yield rate (μmol h^{-1} cm^{-2}) for NO_2^- (blue; triangle) and NH_3 (gray; circle) as a function of the applied potential.

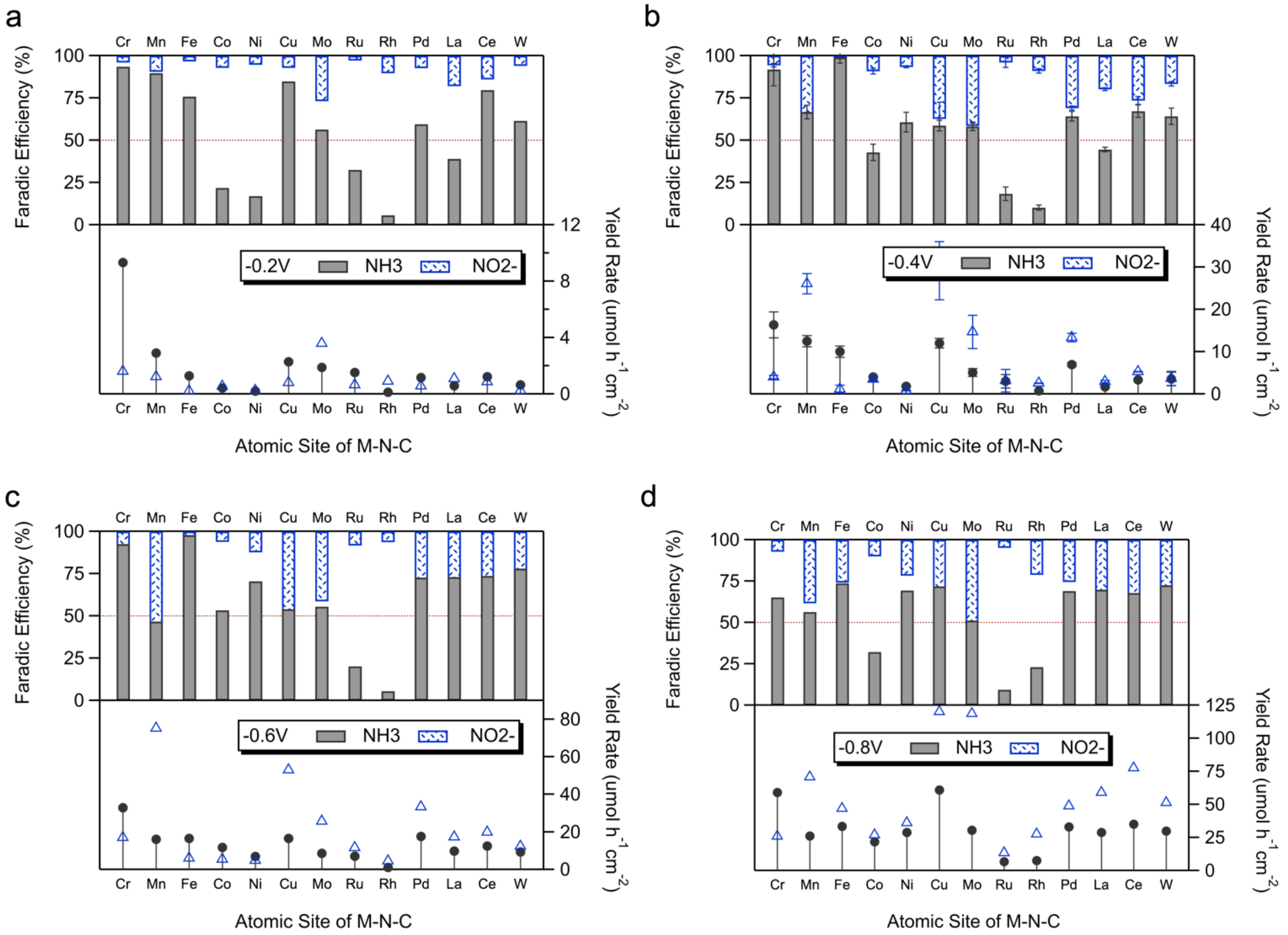

Figure S 40. Electrochemical NO_3RR for all M-N-C catalysts in 0.05M PBS + 0.16M KNO_3 for 2 hours at each applied potential of (a) -0.20 V, (b) -0.40 V, (c) -0.60 V and (d) -0.80 V v.s. RHE. The top section of the figure shows the Faradic efficiency for NO_2^- (blue; top-down) and NH_3 (gray; bottom-up) A red line is set an efficiency of 50% to guide the eye. The bottom section of the figure shows the corresponding yield rate (μmol h^{-1} cm^{-2}) for NO_2^- (blue; triangle) and NH_3 (gray; circle).

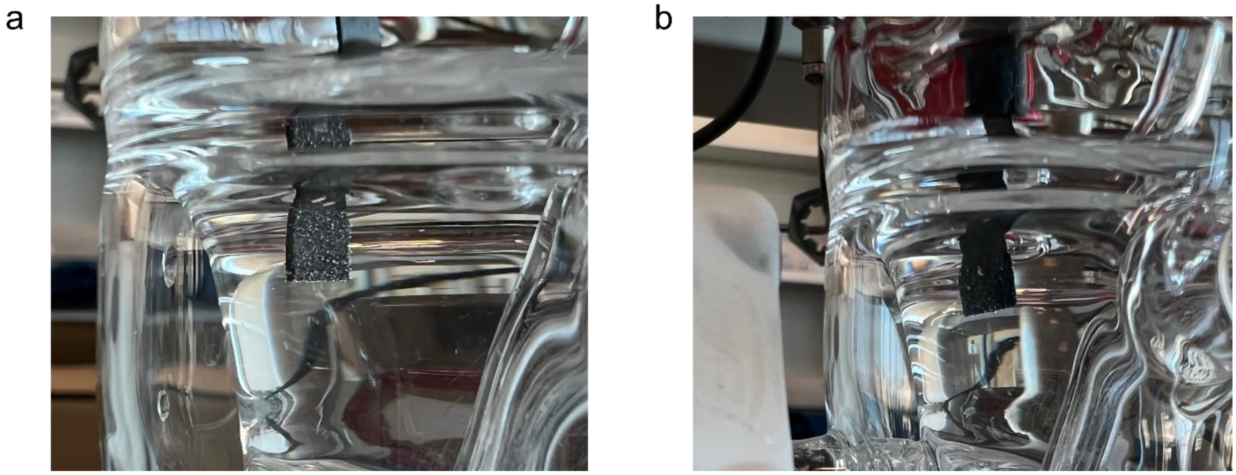

Figure S 41. NO_3RR electrolysis over (a) Ru-N-C and (b) Rh-N-C, showing gaseous bubbles produced on the working electrode. Demonstrating the Ru/Rh-N-C catalysts being out competed by the HER, yielding a large gap in the GAP of Figure 4 and Figure S 38.

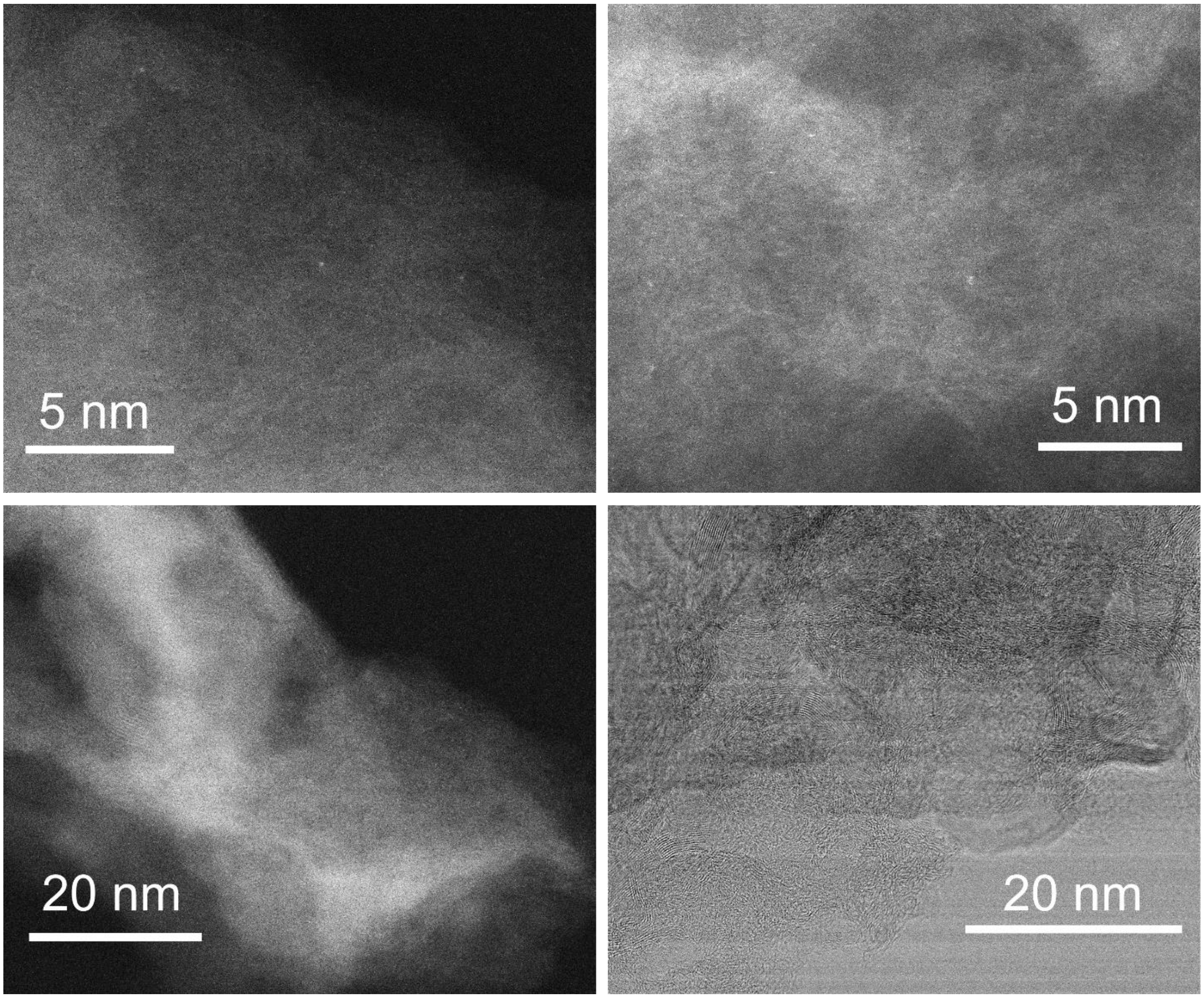

Figure S 42. Post-mortem AC-STEM of the Fe-N-C catalyst after NO_3RR electrolysis at -0.2, -0.4, -0.6 and -0.8 V *vs.* RHE for 2 hours at each potential. High magnification darkfield images show the atomically dispersed Fe sites. Lower magnification darkfield and brightfield images showing the absence of metallic nanoparticles.

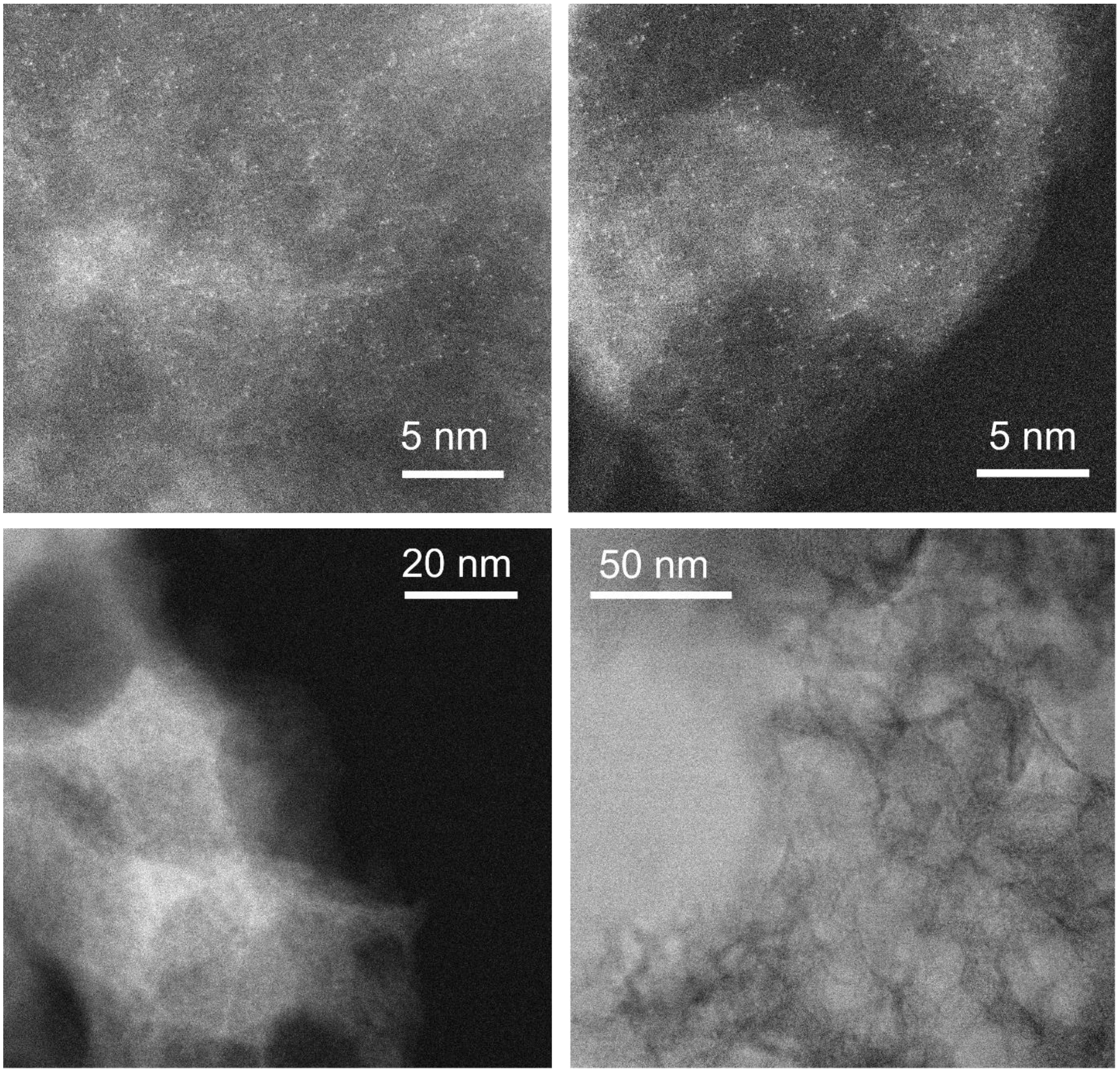

Figure S 43. Post-mortem AC-STEM of the Rh-N-C catalyst after NO_3RR electrolysis at -0.2, -0.4, -0.6 and -0.8 V *vs.* RHE for 2 hours at each potential. High magnification darkfield images show the atomically dispersed Rh sites. Lower magnification darkfield and brightfield images show the absence of metallic nanoparticles.

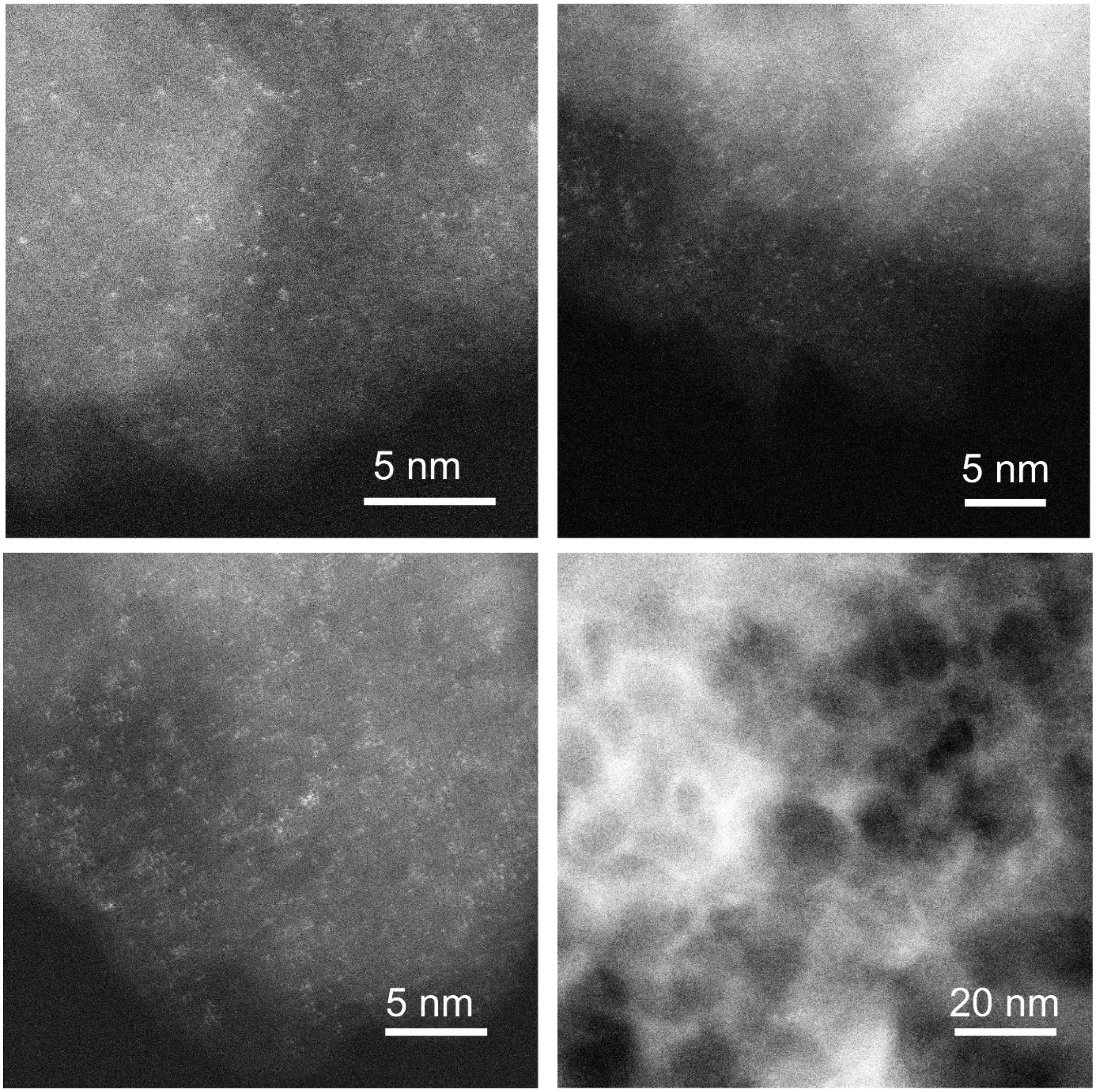

Figure S 44. Post-mortem AC-STEM of the La-N-C catalyst after NO_3RR electrolysis at -0.2, -0.4, -0.6 and -0.8 V *vs.* RHE for 2 hours at each potential. High magnification darkfield images show the atomically dispersed La sites. Lower magnification darkfield image showing the absence of metallic nanoparticles.

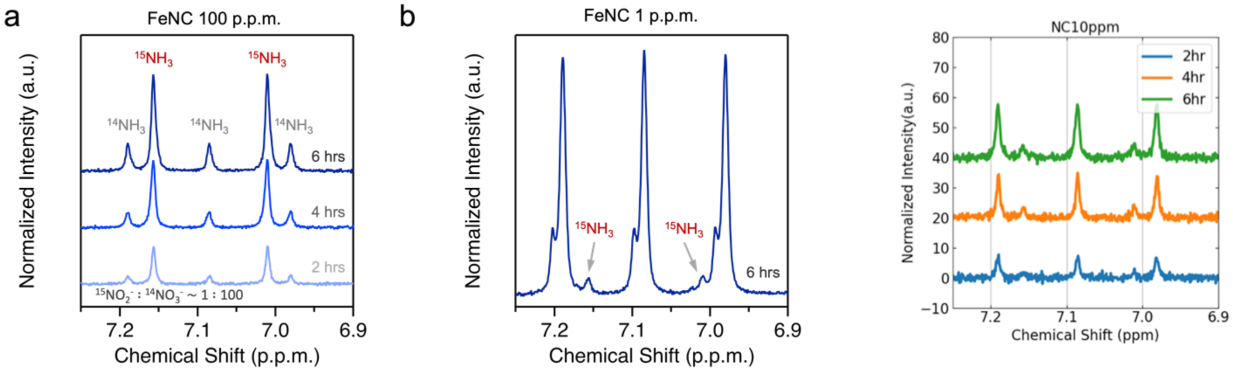

Figure S 45. Competing NO_2RR in concentrated NO_3^-. NO_3RR electrolysis with isotopically labeled $^{15}NO_2^-$ in concentrated $^{14}NO_3^-$. This figure is correlated to Figure 4 in the main text. (a) NMR curves for NH_3 produced during electrolysis of 100 ppm of doped $^{15}NO_2^-$ at -0.40V *vs.* RHE over an Fe-N-C catalyst. (b) 1 ppm of doped $^{15}NO_2^-$. (c) 10 ppm of doped $^{15}NO_2^-$ over a metal free N-C catalyst.

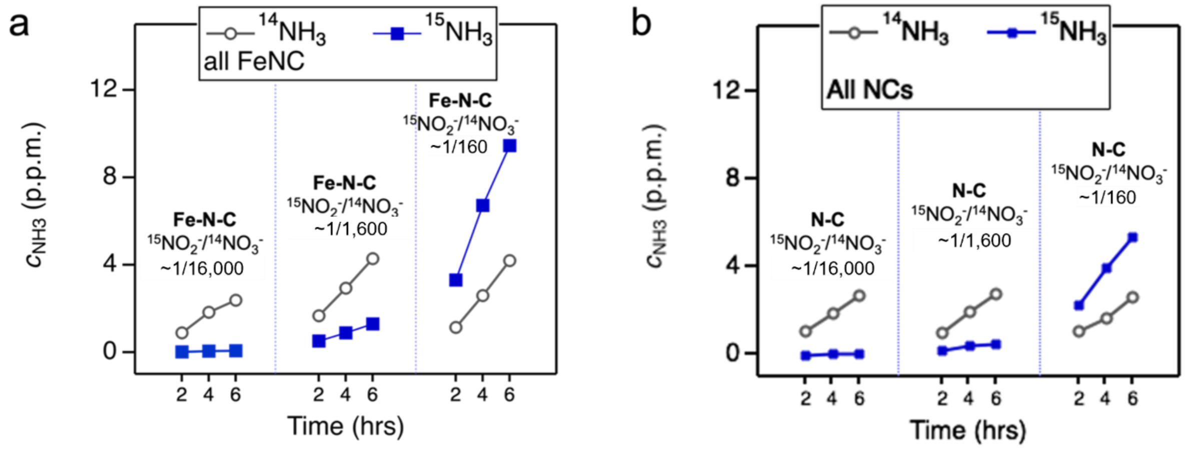

Figure S 46. Time course of $^{15}NH_3$ in the electrolysis with concentrated $^{14}NO_3^-$ doped with small amount $^{15}NO_2^-$. (a) FeNC catalysts. (b) Metal free NC catalyst.

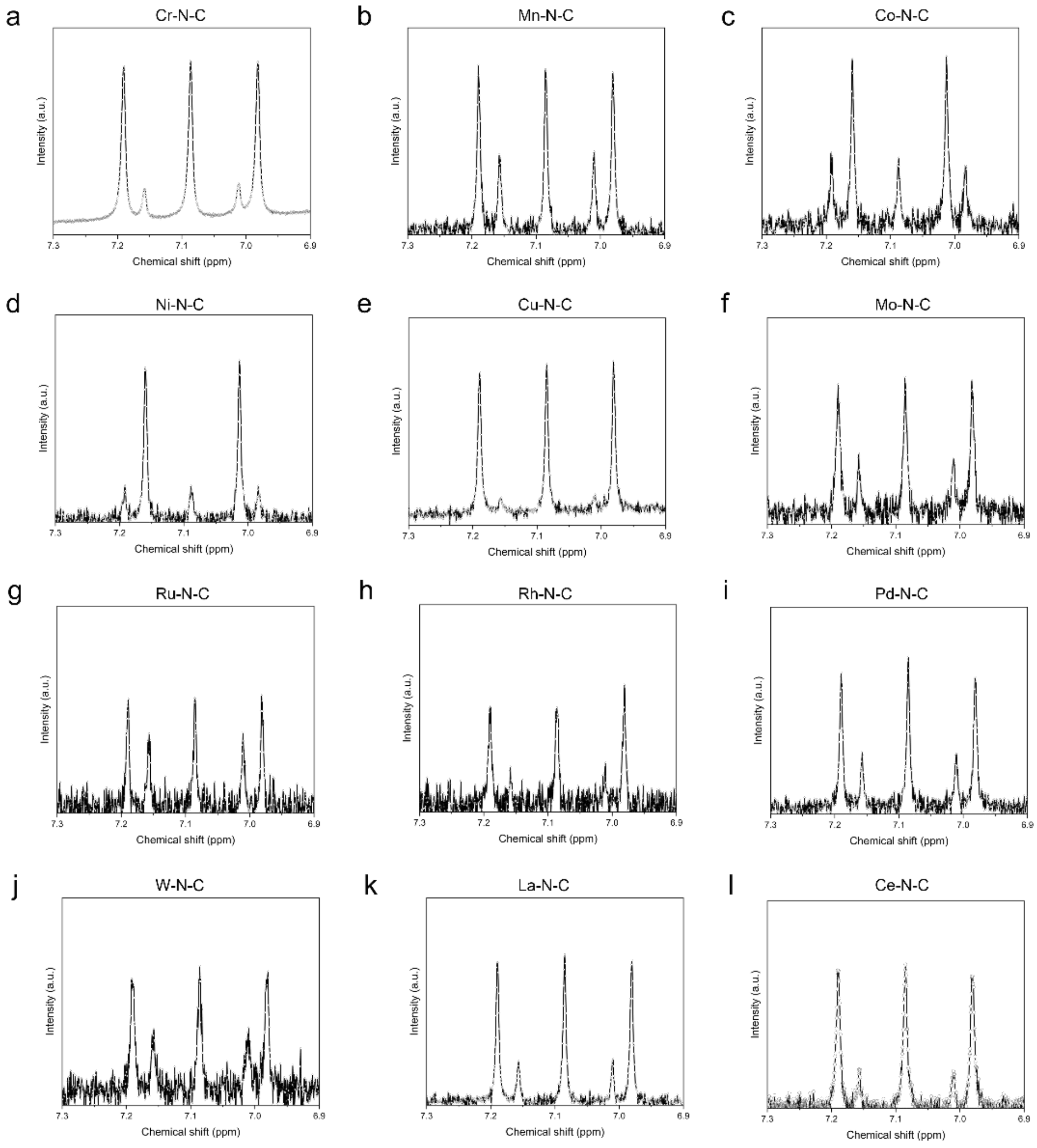

Figure S 47. Competing NO_2RR in concentrated NO_3^-. NO_3RR electrolysis with isotopically labeled $^{15}NO_2^-$ in concentrated $^{14}NO_3^-$ for all M-N-C catalysts. Figures (a – e) are 3*d* metal centers, Figures (f – i) are 4*d* metal centers, Figure (j) is a 5*d* metal center and Figures (k – l) are *f* metal centers. All electrolysis is performed at -0.40 V *vs.* RHE for 2 hours. The electrolyte consists of 0.16M $K^{14}NO_3$ and 10 ppm of isotopically labeled nitrite, $K^{15}NO_2$. For all catalysts (except Co & Ni) there is a dominating triplet indicative of the reduction of the $K^{14}NO_3$ to standard $^{14}NH_3$, with a smaller doublet peak for isotopic $^{15}NH_3$. For Co and Ni, very unique behavior is observed in that these atomically dispersed metal centers show poor activity for the

NO_3RR, but are highly active for the reduction of NO_2^-, as shown by the dominating doublet for $^{15}NH_3$.

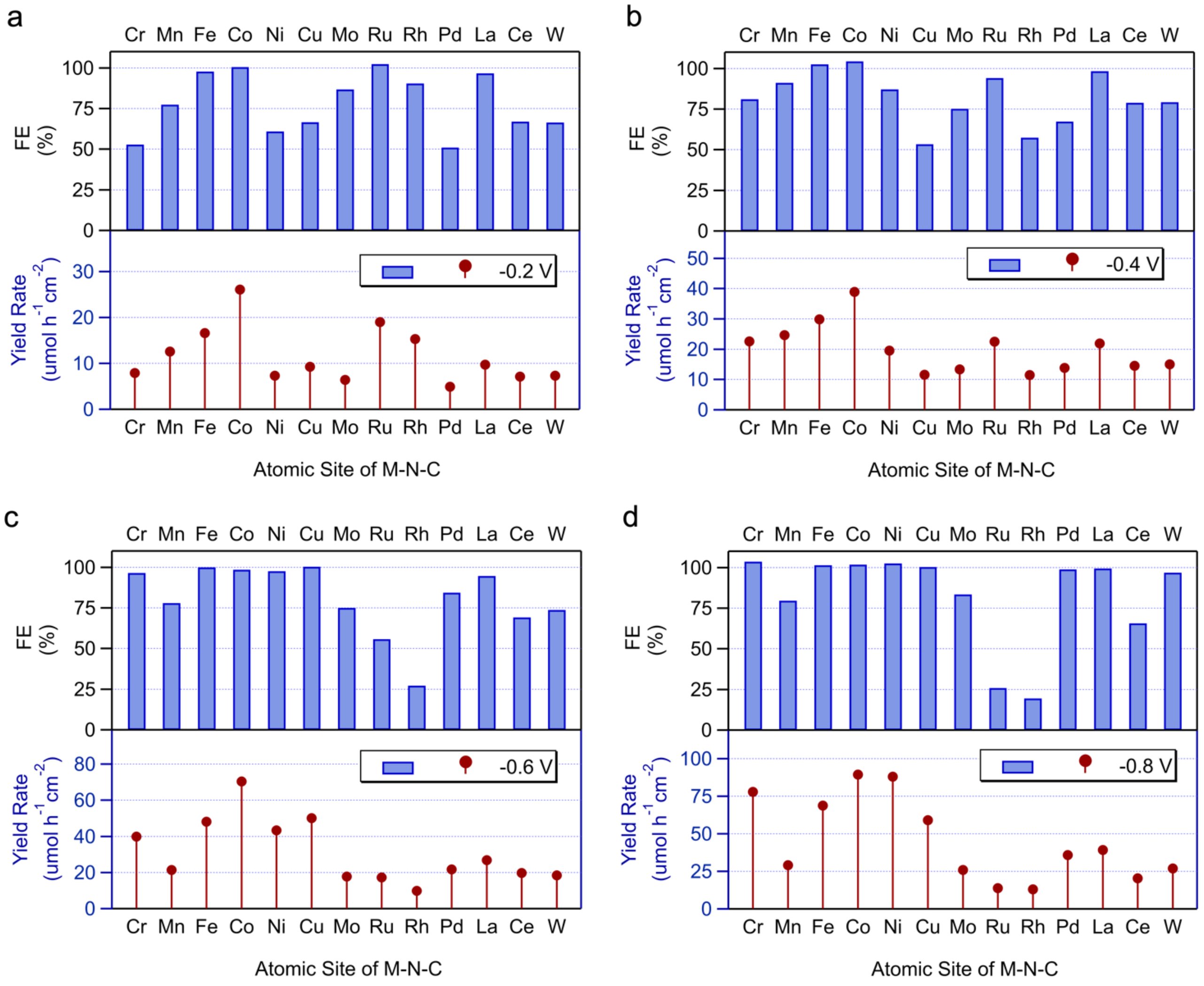

Figure S 48. Electrochemical NO_2RR for all M-N-C catalysts in 0.05M PBS + 0.01M KNO_2 for 30 min at each applied potential of (a) -0.20 V, (b) -0.40 V, (c) -0.60 V and (d) -0.80 V *vs.* RHE. The top section of the figure shows the Faradic efficiency for NO_2^- (blue; top-down) and NH_3 (gray; bottom-up). The bottom section of the figure shows the corresponding yield rate (μmol h^{-1} cm^{-2}) for NH_3.

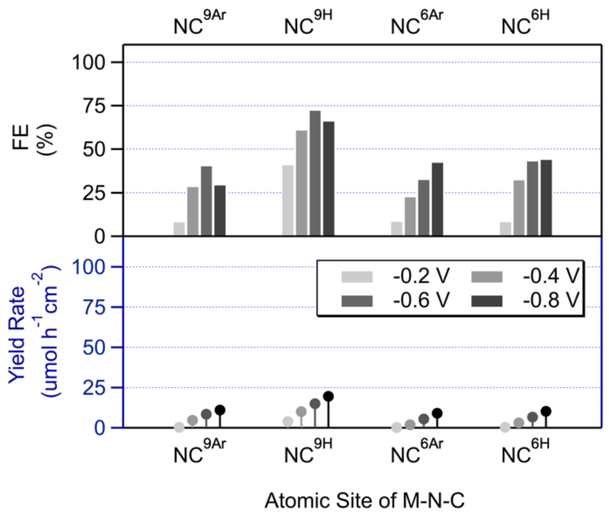

Figure S 49. Electrochemical NO_2RR for metal free N-C catalysts in 0.05M PBS + 0.01M KNO_3 for 30 min at an applied potential of -0.20 to -0.80 V *vs.* RHE. The top section of the figure shows the Faradic efficiency for NO_2^- (blue; top-down) and NH_3 (gray; bottom-up). The bottom section of the figure shows the corresponding yield rate (μmol h^{-1} cm^{-2}) for NH_3 as a function of the applied potential.

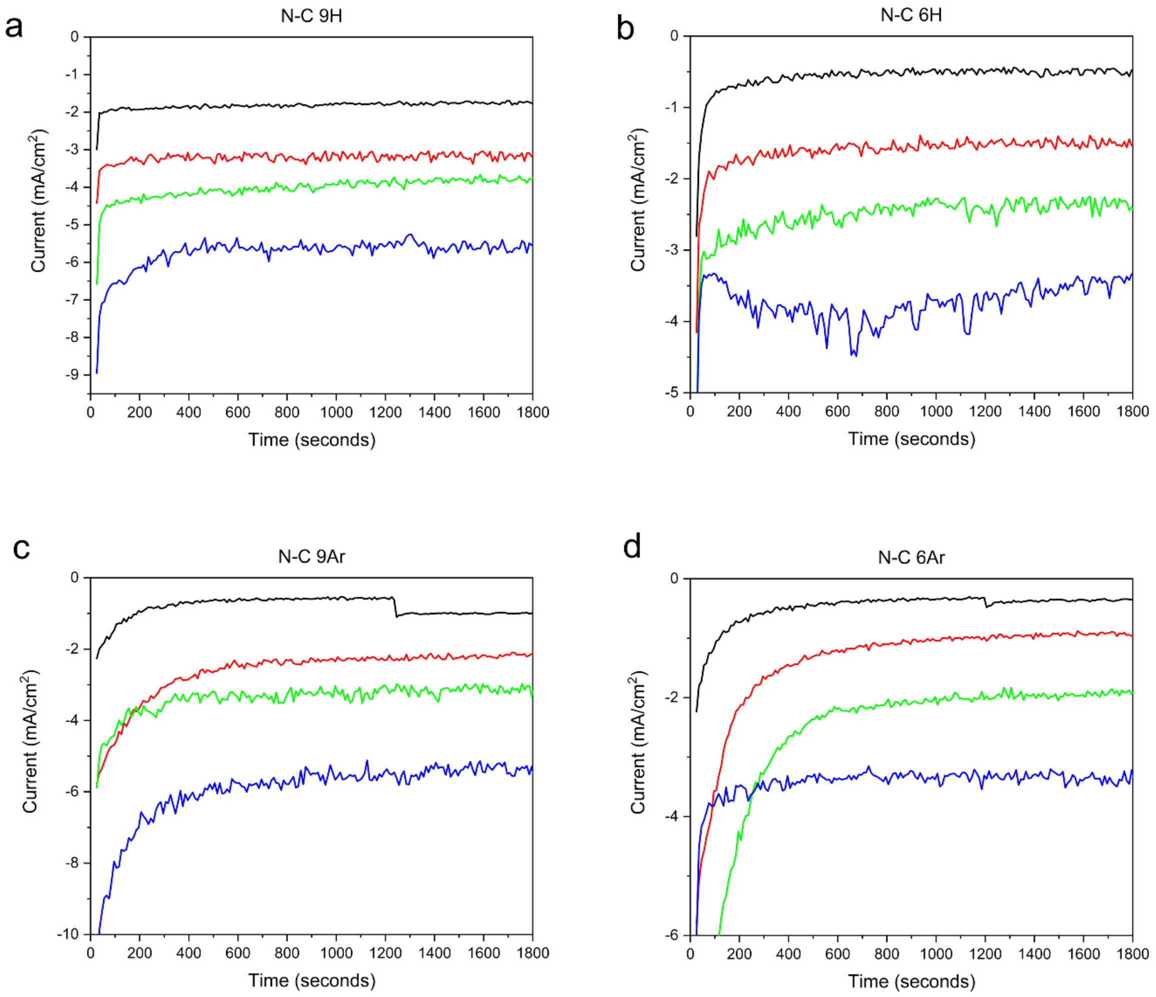

Figure S 50. NO_2RR chronoamperometry on (a) N-C (975 H_2 / 950 Ar), (b) N-C (650 H_2 / 650 Ar), (c) N-C (975 Ar / 950 Ar) and (d) N-C (650 Ar / 650 Ar) catalysts. NO_2RR electrolysis is performed for 0.5 hours in a 0.05M PBS + 0.01M NO_2^- electrolyte. The black, red, green and blue curves correspond to applied potentials of -0.20, -0.40, -0.60 and -0.80 V *vs.* RHE, respectively.

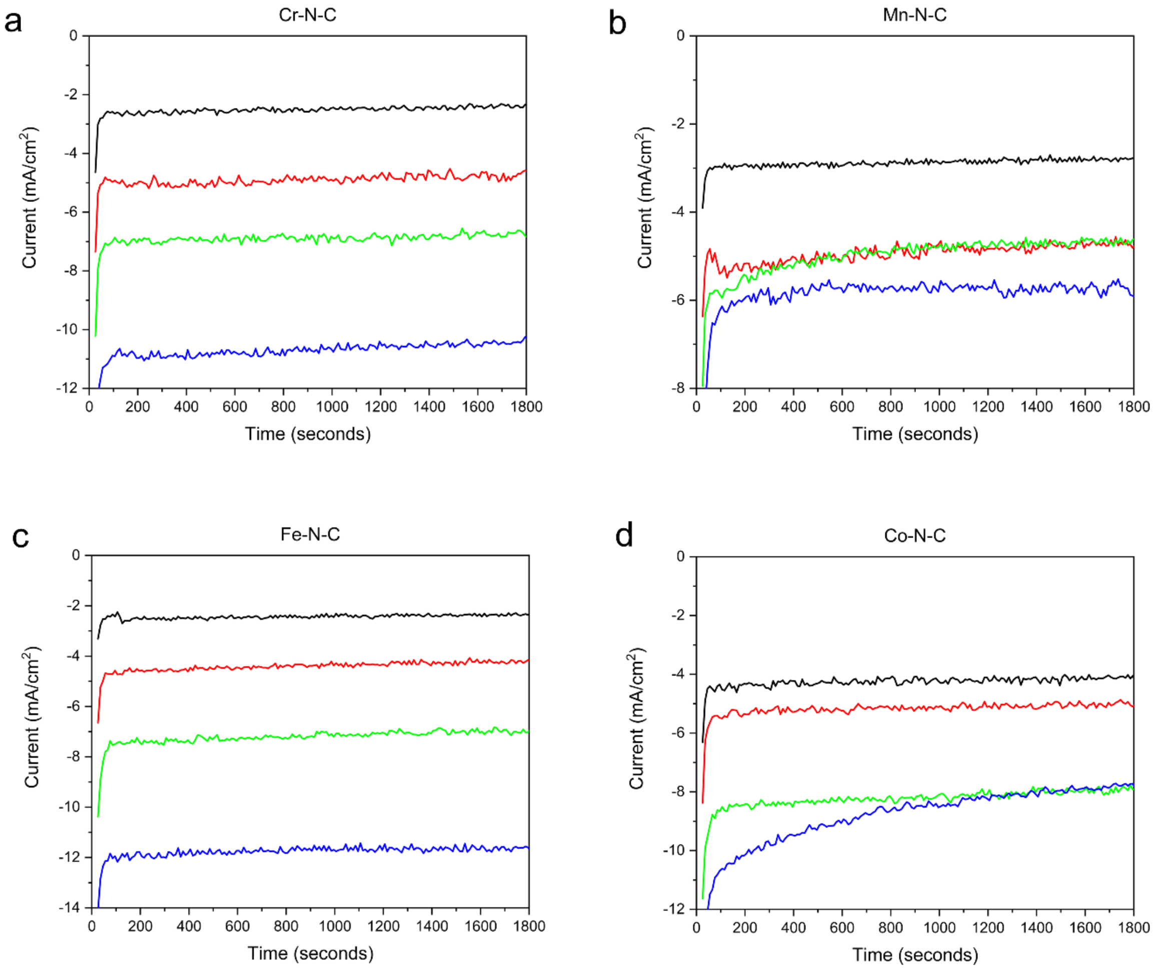

Figure S 51. NO_2RR chronoamperometry on (a) Cr-N-C, (b) Mn-N-C, (c) Fe-N-C and (d) Co-N-C catalysts. NO_2RR electrolysis is performed for 0.5 hours in a 0.05M PBS + 0.01M NO_2^- electrolyte. The black, red, green and blue curves correspond to applied potentials of -0.20, -0.40, -0.60 and -0.80 V *vs.* RHE, respectively.

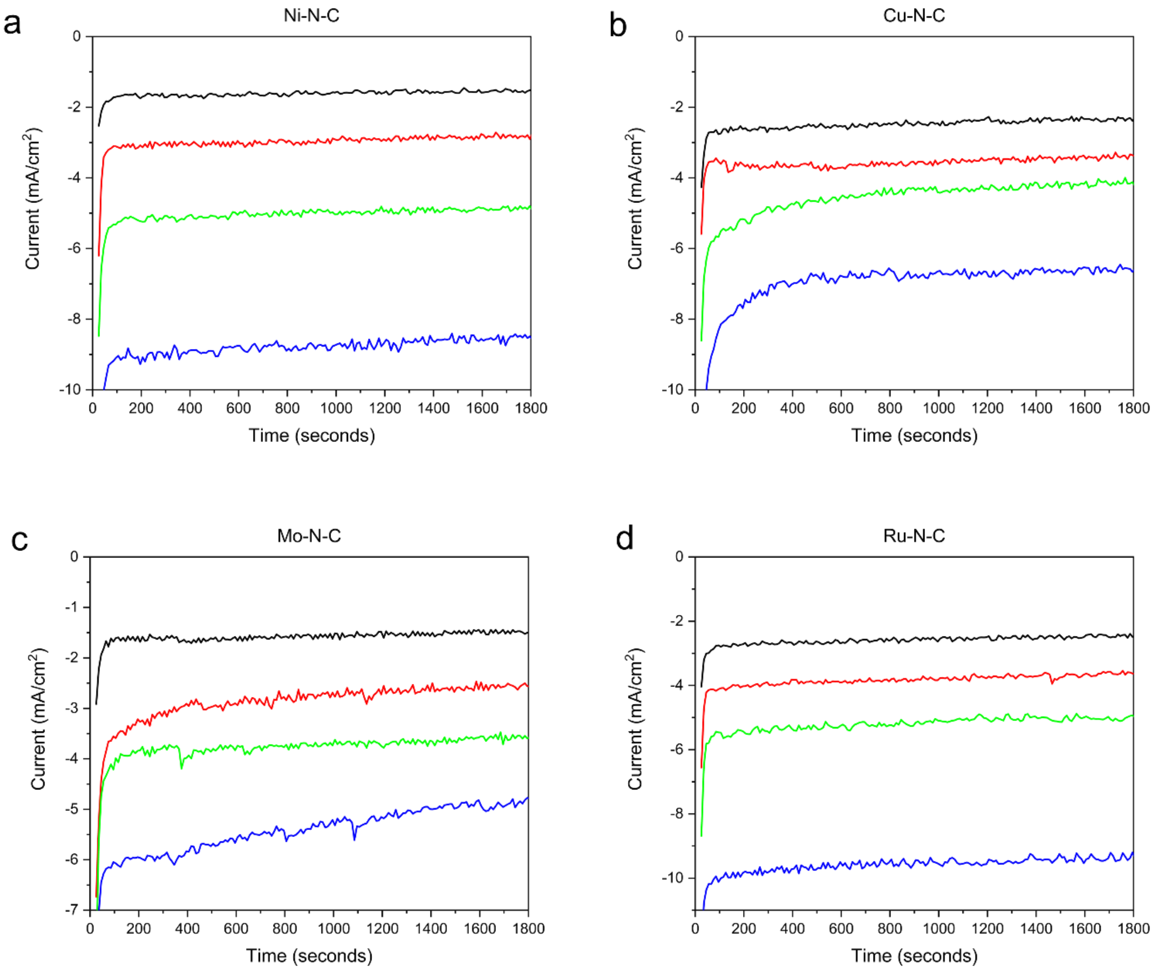

Figure S 52. NO_2RR chronoamperometry on (a) Ni-N-C, (b) Cu-N-C, (c) Mo-N-C and (d) Ru-N-C catalysts. NO_2RR electrolysis is performed for 0.5 hours in a 0.05M PBS + 0.01M NO_2^- electrolyte. The black, red, green and blue curves correspond to applied potentials of -0.20, -0.40, -0.60 and -0.80 V *vs.* RHE, respectively.

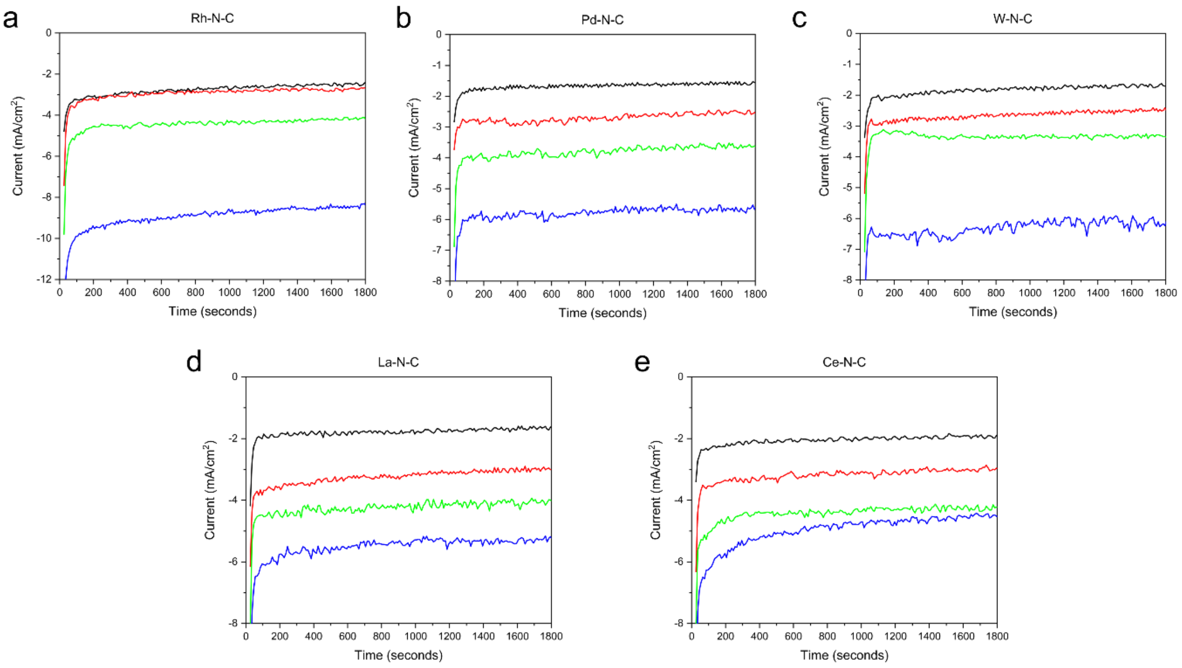

Figure S 53. NO_2RR chronoamperometry on (a) Rh-N-C, (b) Pd-N-C, (c) W-N-C, (d) La-N-C and (e) Ce-N-C catalysts. NO_2RR electrolysis is performed for 0.5 hours in a 0.05M PBS + 0.01M NO_2^- electrolyte. The black, red, green and blue curves correspond to applied potentials of -0.20, -0.40, -0.60 and -0.80 V *vs.* RHE, respectively.

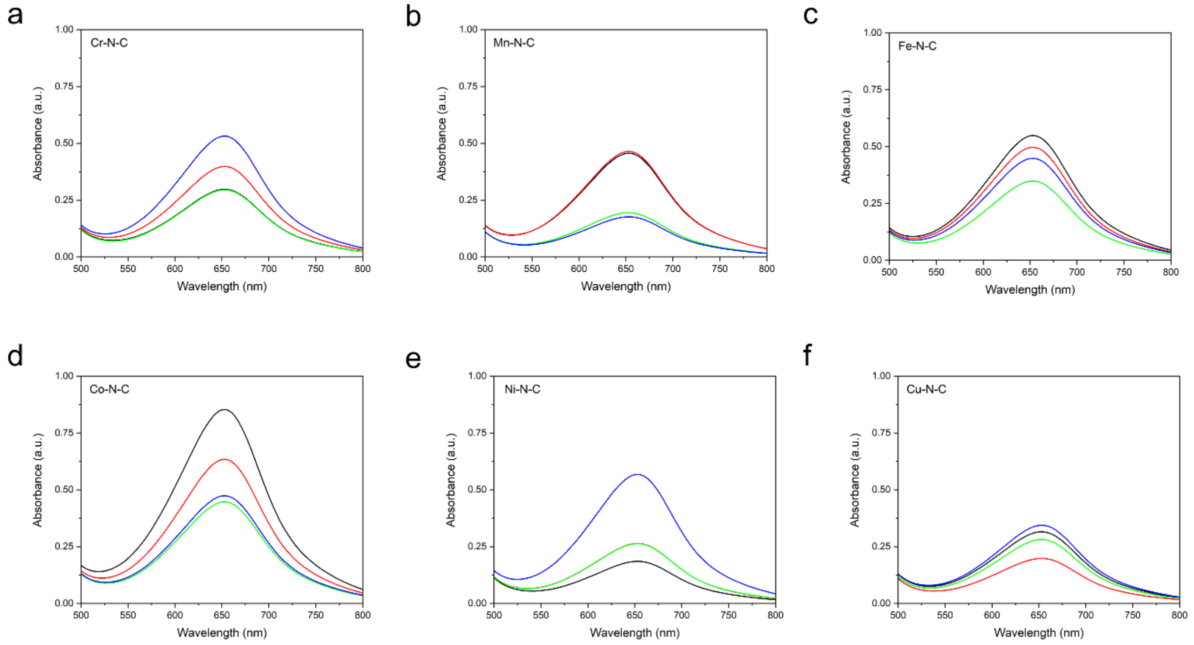

Figure S 54. NO_2RR UV Vis detection of NH_3 for (a) Cr-N-C, (b) Mn-N-C, (c) Fe-N-C, (d) Co-N-C, (e) Ni-N-C and (f) Cu-N-C for 0.5 hr electrolysis at an applied potential of -0.20 V (black), -0.40 V (red), -0.60 V (green) and -0.80 V *vs.* RHE (blue). Dashed curves are spectra from electrolyte in the counter chamber. Dilutions have been applied to obtain a NH_3 concentration in the calibration range.

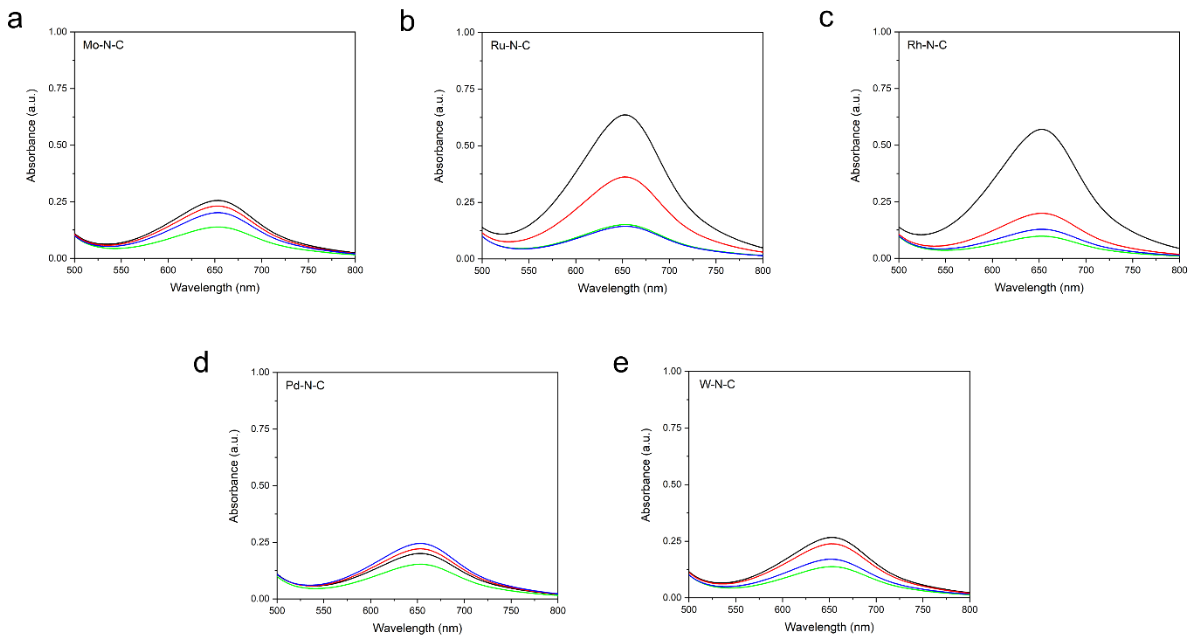

Figure S 55. NO_2RR UV Vis detection of NH_3 for (a) Mo-N-C, (b) Ru-N-C, (c) Rh-N-C, (d) Pd-N-C and (e) W-N-C for 0.5 hr electrolysis at an applied potential of -0.20 V (black), -0.40 V (red), -0.60 V (green) and -0.80 V *vs.* RHE (blue). Dashed curves are spectra from electrolyte in the counter chamber. Dilutions have been applied to obtain a NH_3 concentration in the calibration range.

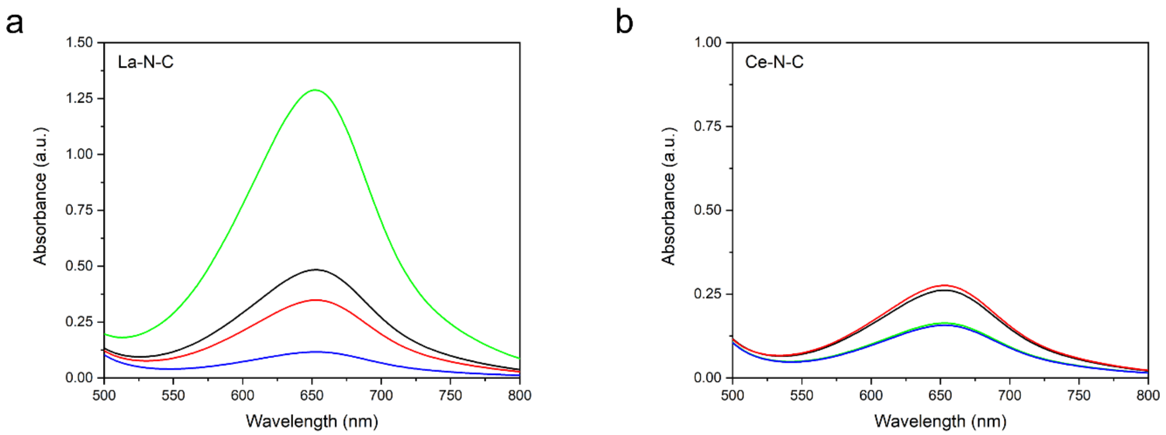

Figure S 56. NO_2RR UV Vis detection of NH_3 for (a) La-N-C and (b) Ce-N-C for 0.5 hr electrolysis at an applied potential of -0.20 V (black), -0.40 V (red), -0.60 V (green) and -0.80 V *vs.* RHE (blue). Dashed curves are spectra from electrolyte in the counter chamber. Dilutions have been applied to obtain a NH_3 concentration in the calibration range.

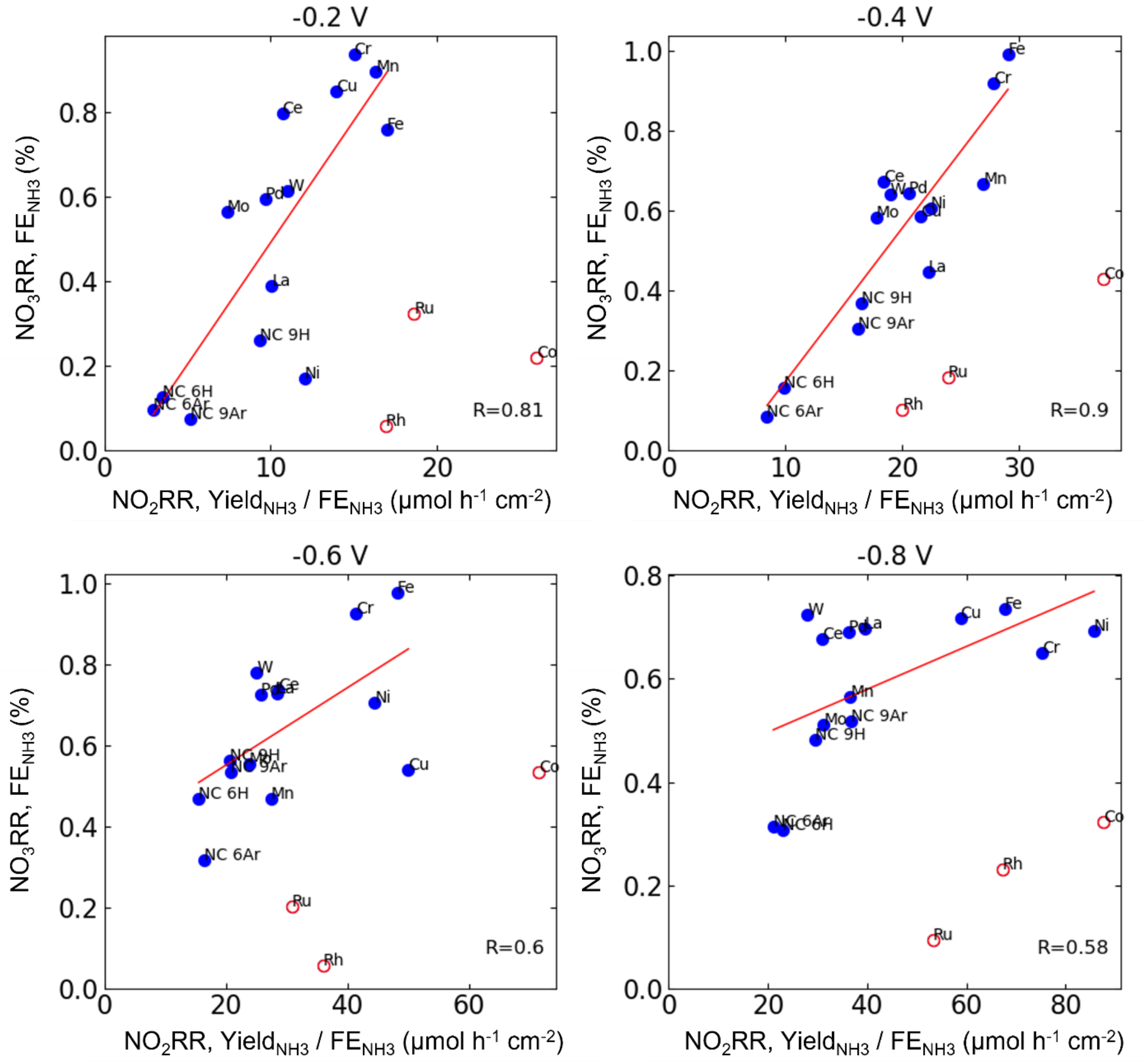

Figure S 57. Correlations between the NO_3RR FE_{NH3} and the NO_2RR dividend of the NH_3 yield rate and NO_2RR FE_{NH3} at cathodic potentials of -0.2 V, -0.4 V, -0.6 V and -0.8V *vs.* RHE. R values represent the linearity of the blue points (also shown in Figure 5g). Red points (Co, Ru, Rh) represent the outliers due to dominant gaseous products (Figure 4 and Figure 5d), and were not included in the linear fitting. The linearity gradually dropped with increasing overpotentials, as the reaction is forcibly driven over various active sites, convoluting the electrolysis.

note: The R values of the linear fitting decreased at more reductive potentials, as at very reductive potentials, the reaction can be forcefully driven over most active sites convoluting the intrinsic activity of the M-N_x site.

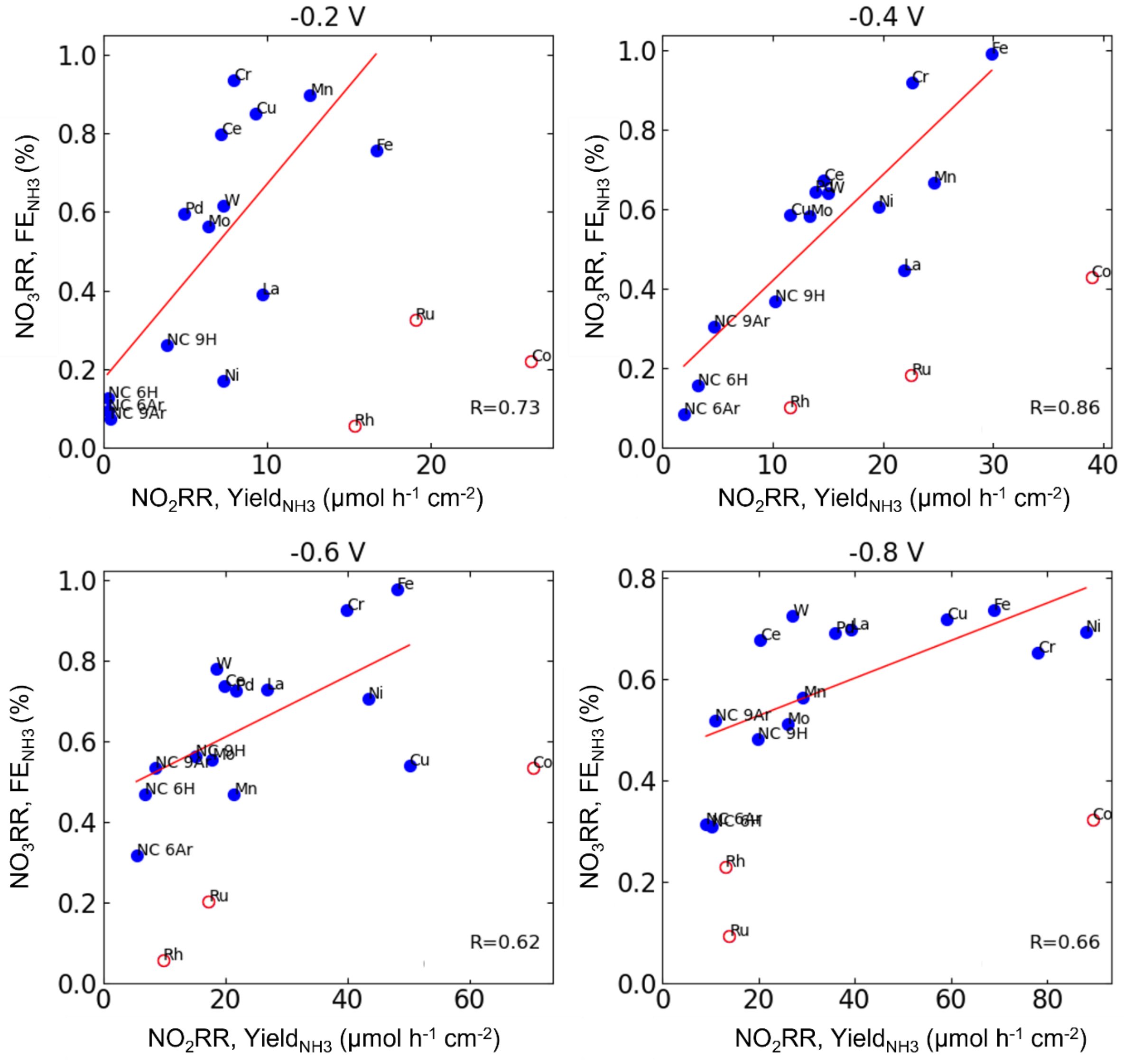

Figure S 58. Correlations between the NO_3RR FE_{NH3} and the NO_2RR NH_3 yield rate at cathodic potentials of -0.2 V, -0.4 V, -0.6 V and -0.8V *vs.* RHE. R values represent the linearity of the blue points. Red points (Co, Ru, Rh) represent the outliers due to dominant gaseous products (Figure 4 and Figure 5d), and were not included in the linear fitting.

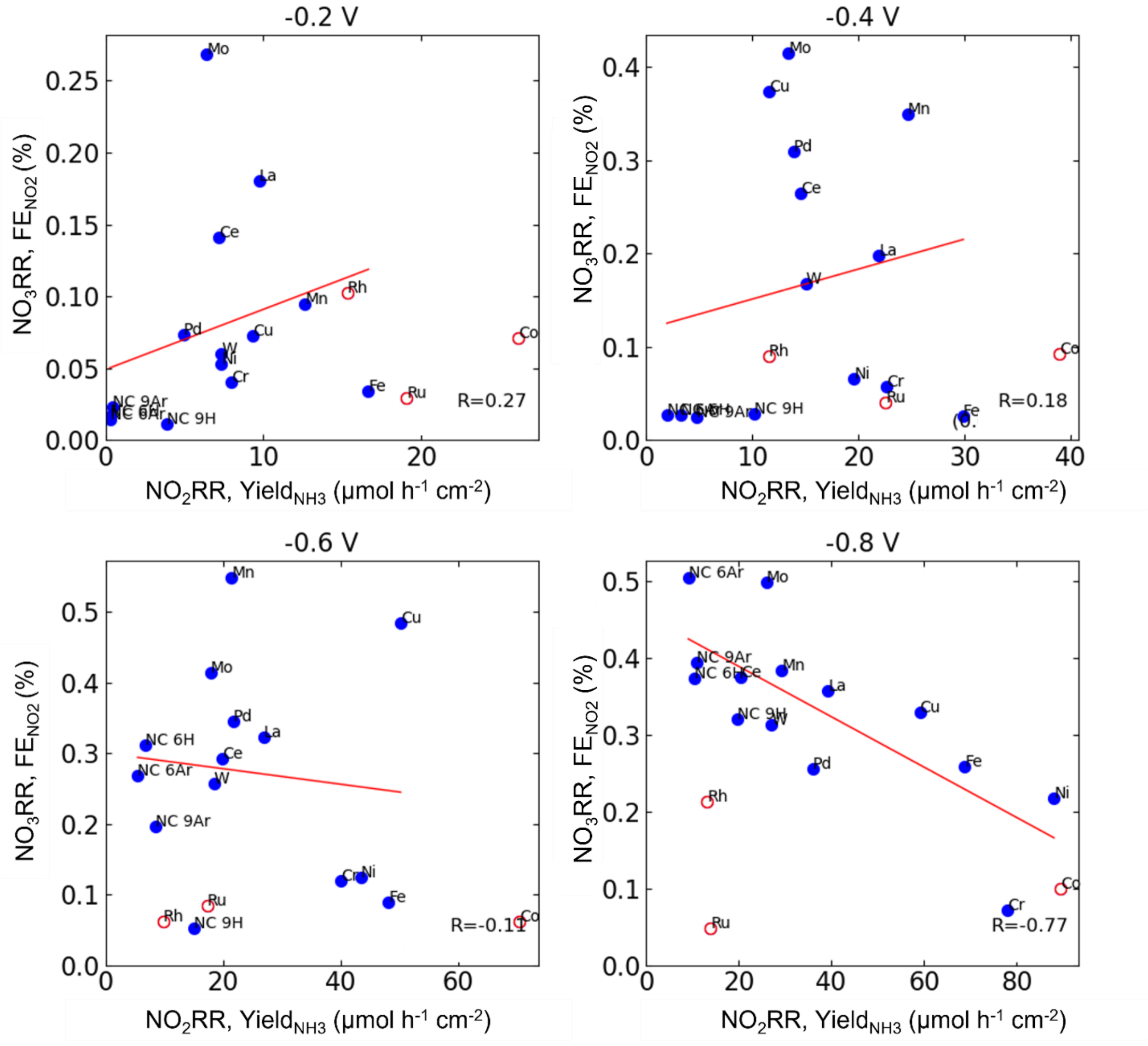

Figure S 59. Correlations between the NO_3RR FE_{NO2} and the NO_2RR NH_3 yield rate at cathodic potentials of -0.2 V, -0.4 V, -0.6 V and -0.8V *vs.* RHE. R values represent the linearity of the blue points (also shown in Figure 5g). Red points (Co, Ru, Rh) represent the outliers due to dominant gaseous products (Figure 4 and Figure 5d), and were not included in the linear fitting.

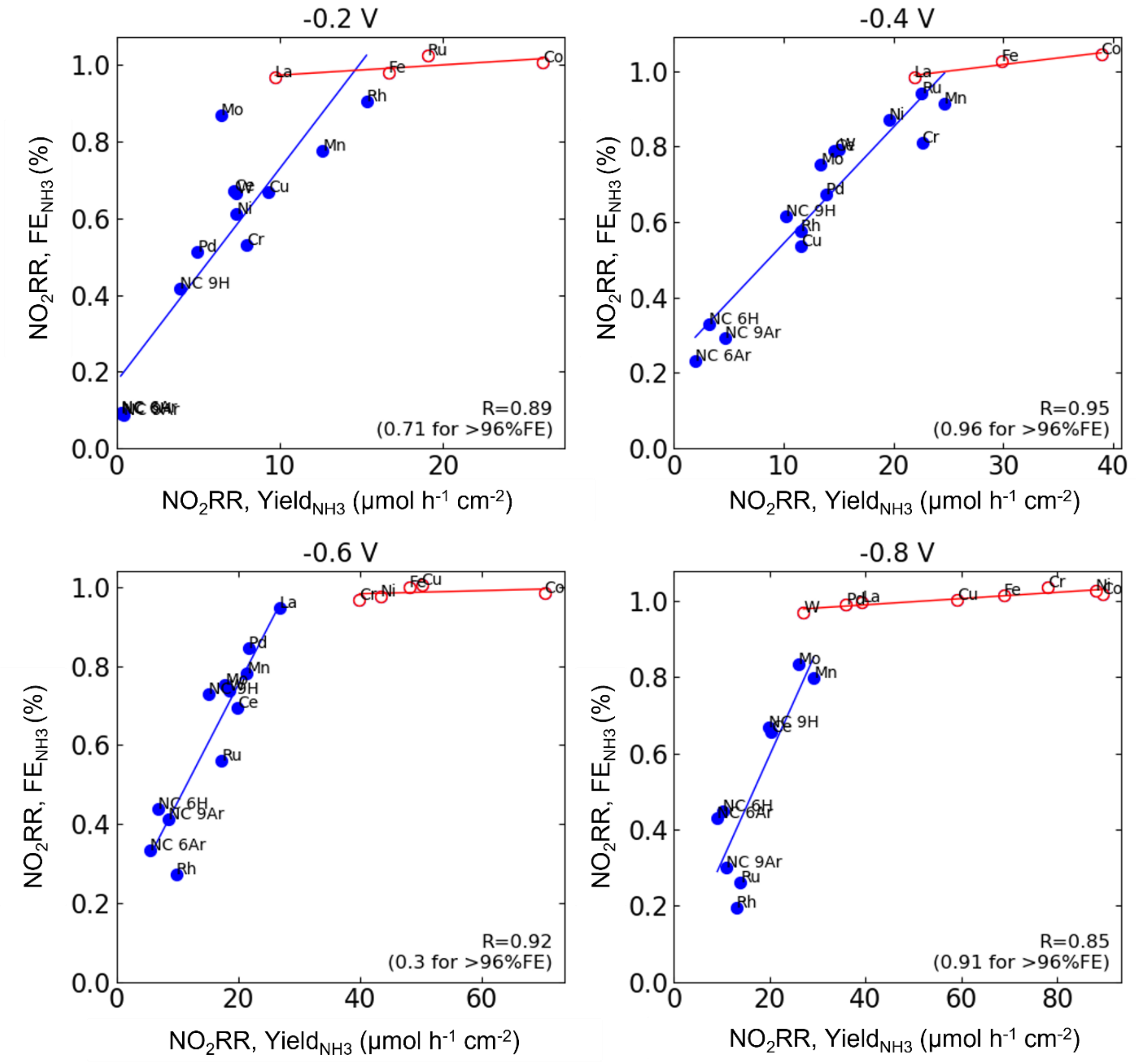

Figure S 60. Correlations between the NO_2RR FE_{NH3} and the NO_2RR NH_3 yield rate at cathodic potentials of -0.2 V, -0.4 V, -0.6 V and -0.8V *vs.* RHE. R values represent the linearity of the blue points (also shown in Figure 5g). Red points represent the catalysts reaching 100% FE at each potential, and were not included in the linear fitting.

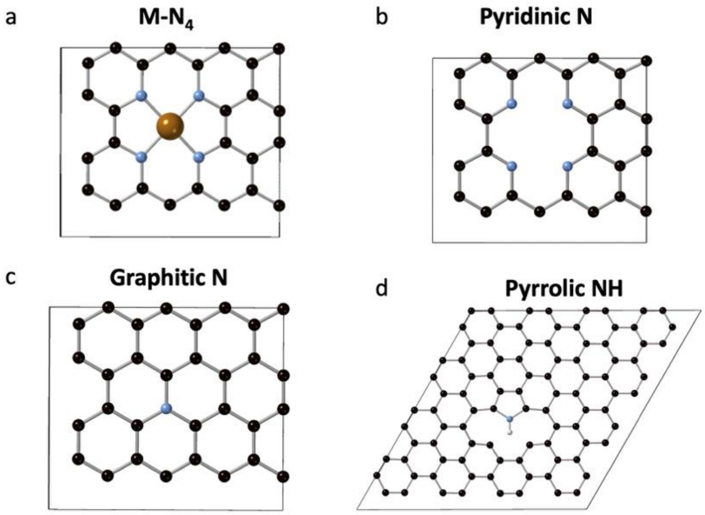

Figure S 61. Active sites used in the DFT calculation of reactivity descriptors for the NO_3RR and the NO_2RR. a) M-N_4 site, b) pyridinic N, c) graphitic N, and d) pyrrolic NH. Atoms belonging to one unit cell are shown. Black – C, blue – N, white – H, tan – metal atom.

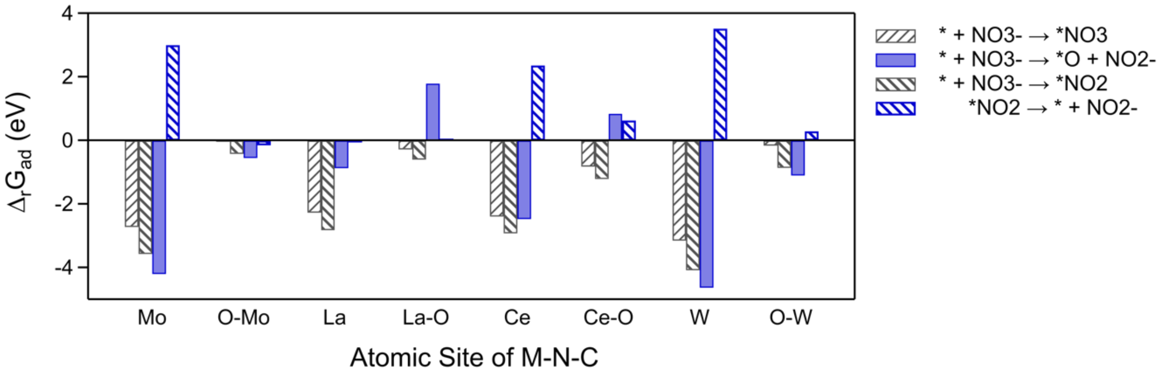

Figure S 62. DFT-derived free energies on Mo-, La-, Ce- and W-N-C sites and their oxygenated (O-M) states.

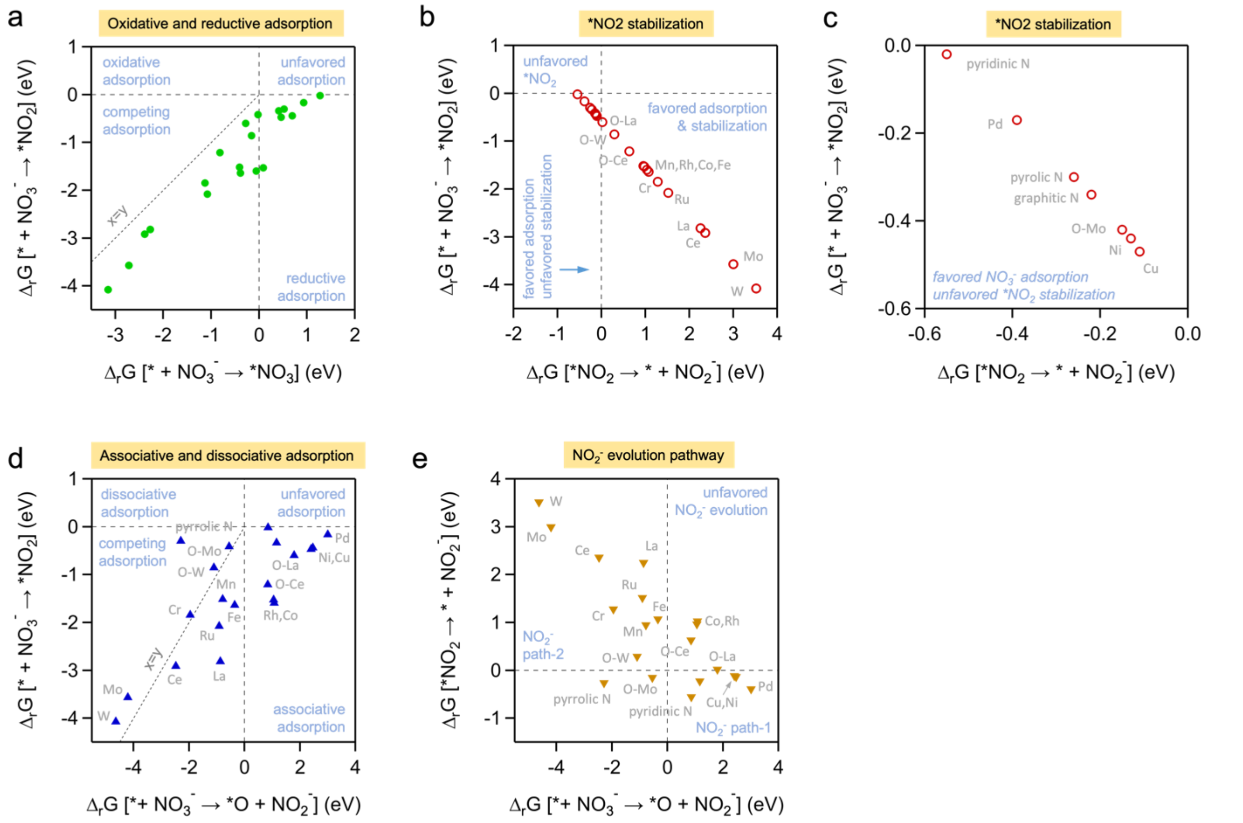

Figure S 63. Correlations between various DFT-derived free energies ($\Delta_r G$) in Figure 5a. (a) Oxidative and reductive adsorption, (b) *NO_2 stabilization, (c) zoomed in *NO_2 stabilization for more clarity, (d) associative and dissociative adsorption and (e) NO_2^- evolution. It should be noted that $\Delta_r G[\,^*+NO_3^- \rightarrow \,^*NO_2]$ and $\Delta_r G[\,^*NO_2 \rightarrow \,^*+NO_2^-]$ were dependent terms, given the fact that they shared same surface intermediate species $^*NO_2^-$ and all other species in these two reaction coordinates were bulk items

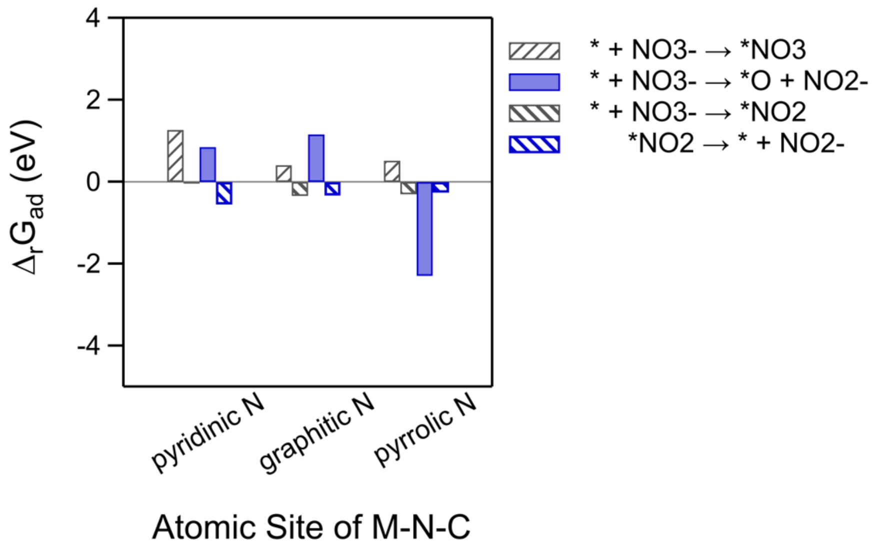

Figure S 64. DFT-derived adsorption/desorption energies on different nitrogen moieties.

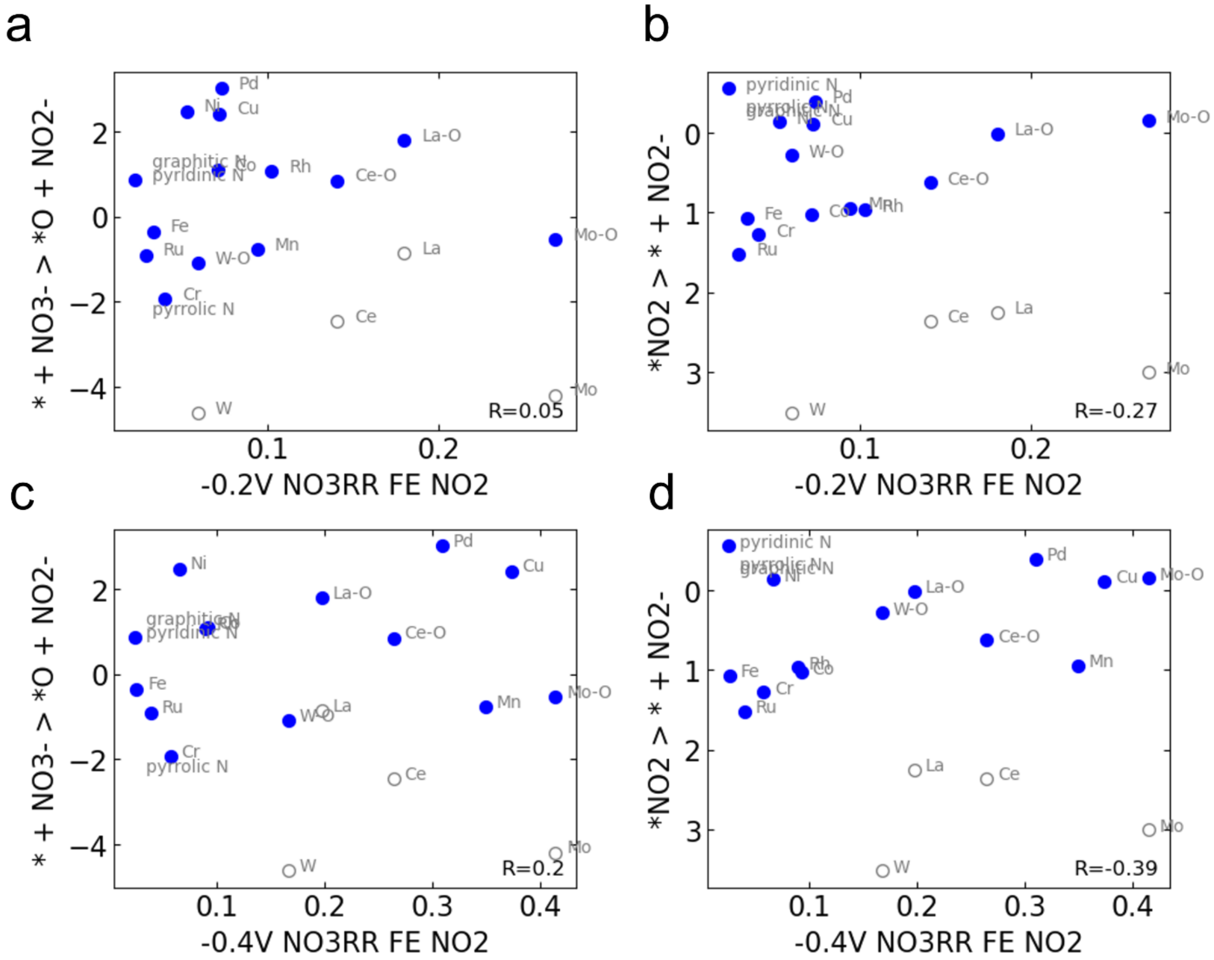

Figure S 65. Correlations between various DFT-derived descriptors $\Delta_r G\ [{}^{*}NO_2 \rightarrow {}^{*} + NO_2^-]$ or $\Delta_r G\ [{}^{*} + NO_3^- \rightarrow {}^{*}O + NO_2^-]$, and the NO_3RR FE_{NO2-} at (a) -0.20, (b) -0.40, (c) -0.60, (d) -0.80 V *vs.* RHE.

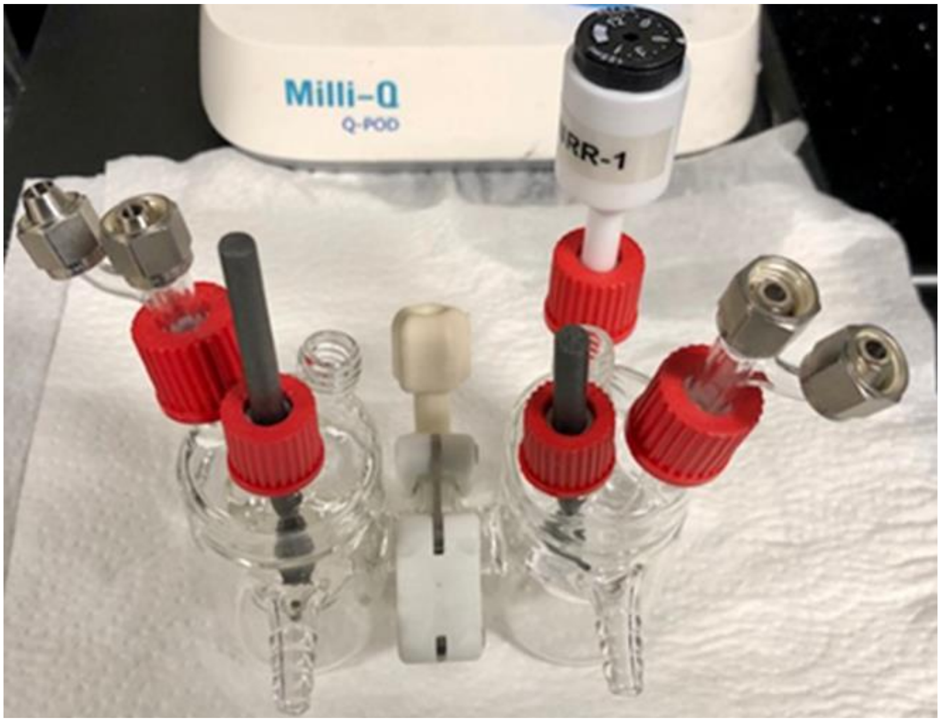

Figure S 66. Electrochemical H-cell used in this work. Left hand side is the counter chamber with the graphite counter working electrode. The right-hand side contains the RHE reference electrode (in a separate chamber connected through a glass frit) and graphite rod with the carbon paper working electrode. The septum on both the left and right hand side allow for gas inlet and outlet into the working and counter chambers.

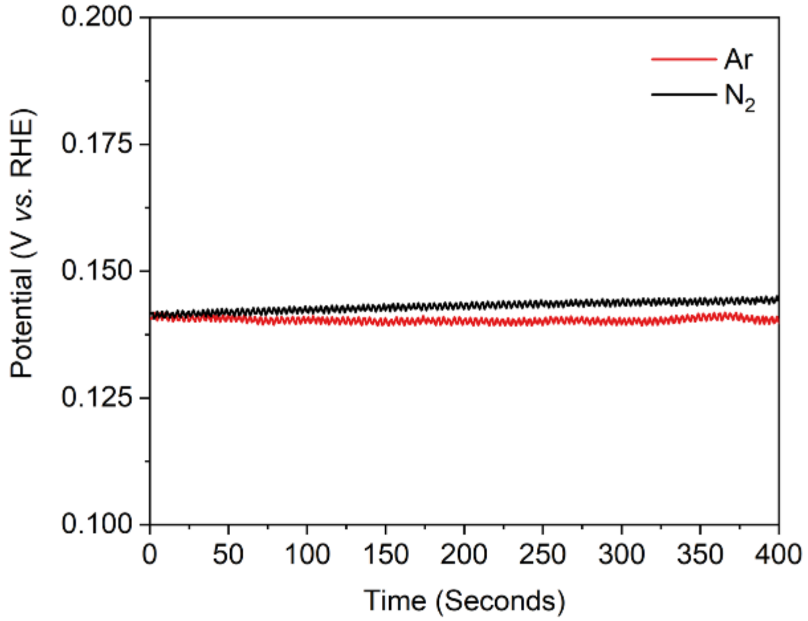

Figure S 67. Open circuit voltage (OCV) measurements when the electrolyte is saturated in N_2 or Ar. The negligible difference in the OCV suggests that the use of either N_2 or Ar does not alter the equilibrium potentials of the NO_3RR process.

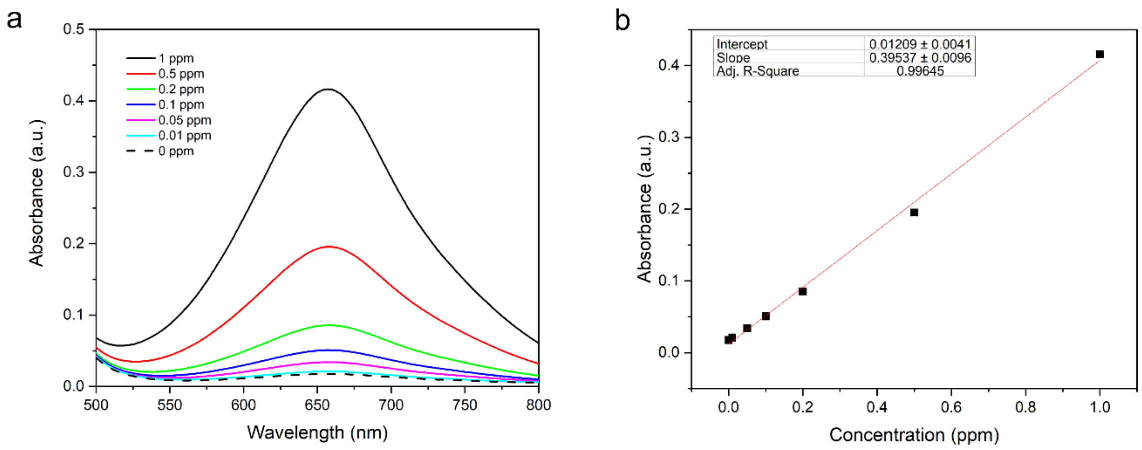

Figure S 68. Calibration curves for the Indophenol blue method for NH_3 detection in 0.05M PBS electrolyte. (a) Raw UV-Vis curves and (b) linear calibration.

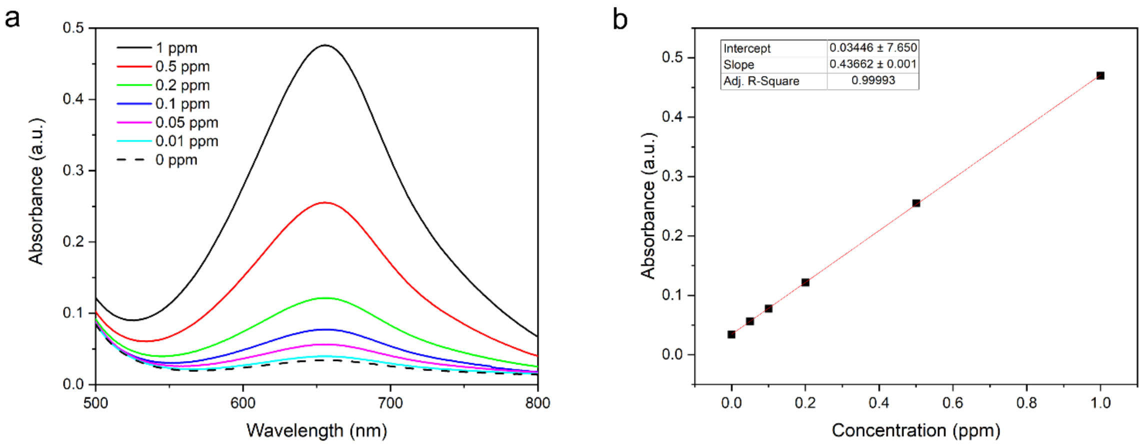

Figure S 69. Calibration curves for the Indophenol blue method for NH_3 detection in 1M KOH electrolyte. (a) Raw UV-Vis curves and (b) linear calibration.

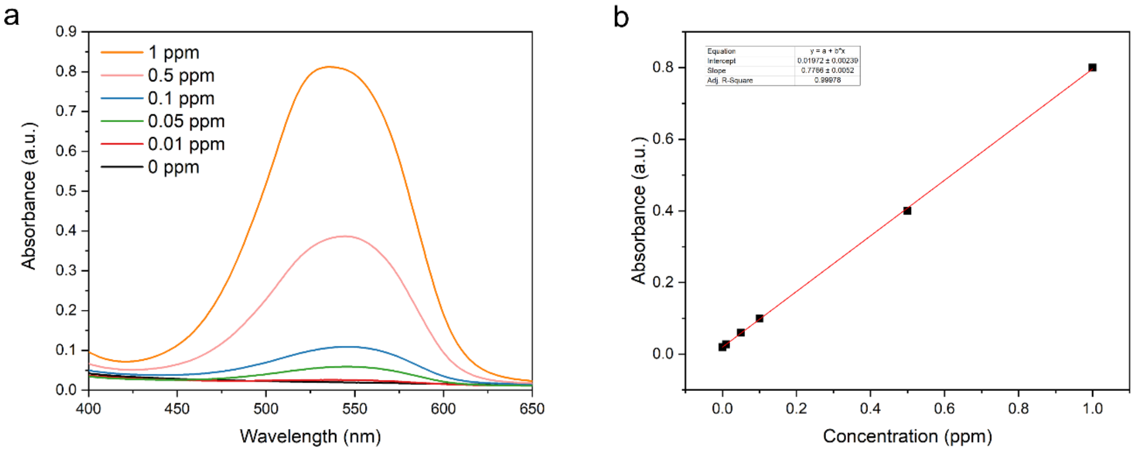

Figure S 70. Calibration curves for the Griess method for NO_2^- detection in 0.05M PBS electrolyte . (a) Raw UV-Vis curves and (b) linear calibration.

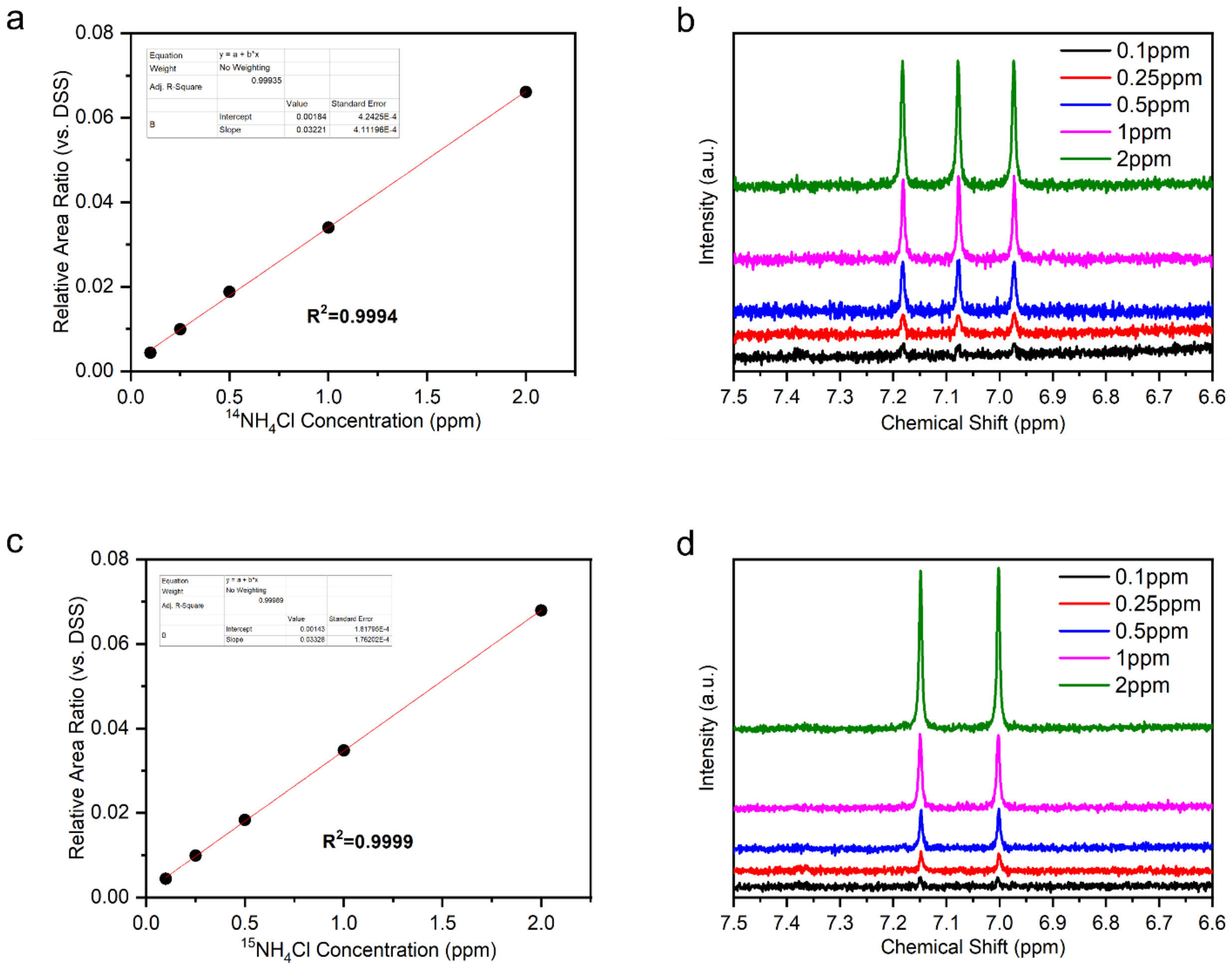

Figure S 71. Calibration curves for the detection of NH_3 with ^1H NMR. (a) Linear calibration for the detection of $^{14}NH_4$, (b) ^1H NMR spectra for $^{14}NH_4$, (c) Linear calibration for the detection of isotopic $^{15}NH_4$, (d) ^1H NMR spectra for $^{15}NH_4$.

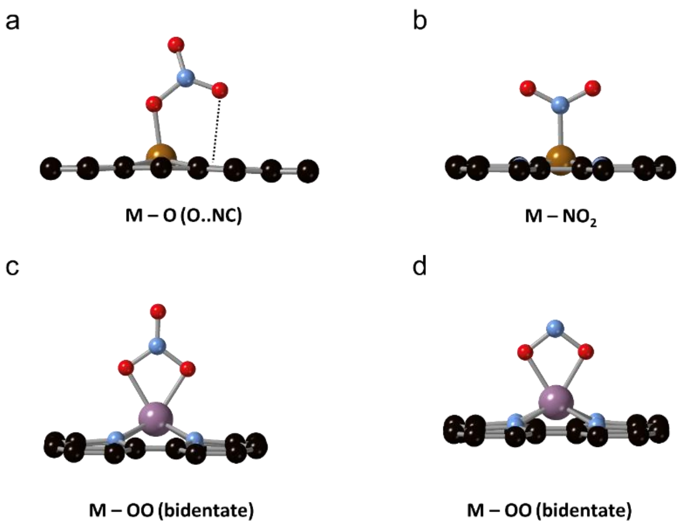

Figure S 72. Schematically represented DFT determined $*NO_3$ and $*NO_2$ preferred orientations on different in-plane and out-of-plane $M-N_4$ sites (See table S9). (a) M-O and O-N of NO_3, (b) M-NO_2, (c) M-OO (bidentate) from $*NO_3$ and (d) M-OO (bidentate) from $*NO_2$.

Table S1. Summary of optimized synthesis conditions to obtain atomically dispersed metal sites with a variety of M-N-C catalysts.

Catalyst	Pyrolysis 1 Temperature	Pyrolysis 1 Environment	Pyrolysis 2 Temperature	Pyrolysis 2 Environment	Etching Media
Cr-N-C	650 ˚C	8% H_2 / 92% Ar	650 ˚C	10% NH_3 / 90% N_2	HF
Mn-, Fe-, Ni-, Co-, Cu-, Mo-, W-N-C	975 ˚C	8% H_2 / 92% Ar	950 ˚C	10% NH_3 / 90% N_2	HF
Ru-N-C	650 ˚C	Ar	650 ˚C	Ar	HF
Rh-, Pd-N-C	975 ˚C	Ar	950 ˚C	Ar	HF
La-, Ce-N-C	650 ˚C	Ar	650 ˚C	Ar	NaOH - 80˚C

See Supplementary note 2 for a discussion on tuning the synthesis parameters to maintain an atomic dispersion of the metal species.

Table S2. Structure parameters from the EXAFS fittings for the M-N-C catalysts including coordination number, interatomic distances (Metal-Oxygen, $R_{M\text{-}O}$ and Metal-Nitrogen, $R_{M\text{-}N}$) and disorder factor σ^2.

**Sample**	**S_0^2 factor**	**Coordination number**	**R $_{M\text{-}N / M\text{-}O}$ (Å)**	**σ^2 $_{M\text{-}N / M\text{-}O}$ (Å^2)**	**ΔE_0 (eV)**	**R factor**
Cr-N-C		2.4 ± 0.3	1.97 ± 0.001	0 ± 0.003	5.31 ± 2.8	0.81%
Cr_2O_3	0.56	6	1.96 ± 0.01	0.002 ± 0.002	4.18 ± 1.1	0.13%
Mn-N-C		7.9 ± 1.4	2.12 ± 0.1	0.017 ± 0.001	-5.52 ± 0.7	0.01%
MnO	0.48	6	2.2 ± 0.03	0.002 ± 0.003	-5.08 ± 2.1	0.46%
Fe-N-C		5.98 ± 1.1	2.12 ± 0.1	0.009 ± 0.005	8.49 ± 1.6	0.37%
Fe_3O_4	0.53	5.3	2.03 ± 0.1	0.005 ± 0.005	8.56 ± 1.3	0.25%
Co-N-C		8.4 ± 0.9	1.92 ± 0.02	0.021 ± 0.002	-5.82 ± 1.1	0.46%
Co_3O_4	0.54	5.3	1.91 ± 0.02	0 ± 0.005	-6.41 ± 3.4	0.90%
Ni-N-C		1.8 ± 0.3	1.89 ± 0.2	0.01 ± 0.003	-7.58 ± 1.7	0.21%
$Ni(OH)_2$	0.99	6	2.08 ± 0.06	0.006 ± 0.004	-11.41 ± 2.9	0.60%
Cu-N-C		5.2 ± 0.4	1.9 ± 0.06	0.005 ± 0.001	-5.84 ± 1.0	0.07%
CuO	0.41	4	1.94 ± 0.02	0.001 ± 0.002	-4.5 ± 1.2	0.11%
Rh-N-C		4.1 ± 0.6	1.99 ± 0.05	0.004 ± 0.002	-1.31 ± 1.8	0.26%
Rh_2O_3	0.67	6	2.02 ± 0.02	0 ± 0.001	-0.57 ± 0.8	0.05%
La-N-C		8.2 ± 1.4	2.61 ± 0.3	0.03 ± 0.006	5.77 ± 1.0	1.74%
La_2O_3	1.4	7	2.6 ± 0.2	0.015 ± 0.003	5.57 ± 0.6	0.56%
Ce-N-C		0.35 ± 0.1 / 5.5 ± 0.6	1.65 ± 0.7 / 2.60 ± 0.2	0.00 ± 0.04 / 0.015 ± 0.02	1.29 ± 5.7	0.91%
CeO_2	0.70	8	2.27 ± 0.1	0.011 ± 0.004	5.97 ± 1.7	1.58%

See supplementary note 3 for a discussion regarding the EXAFS fitting results and coordination numbers.

Table S3. Summary of total nitrogen and oxygen content for all metal free N-C catalysts.

Catalyst	Total N (At %)	Total O (At %)
N-C (650-H_2 / 650-NH_3)	11.13%	3.25%
N-C (975-H_2 / 950-NH_3)	3.22%	6.87%
N-C (650-Ar / 650-Ar)	12.48%	3.13%
N-C (975-Ar / 950-Ar)	3.18%	7.48%

Table S4. Summary of total nitrogen and oxygen content for all M-N-C catalysts.

**Catalyst**	**Total N (At %)**	**Total O (At %)**
Cr	12.10%	3.71%
Mn	2.86%	10.65%
Fe	2.71%	5.40%
Co	3.60%	1.79%
Ni	2.61%	2.04%
Cu	4.23%	3.20%
Mo	1.07%	2.70%
Ru	12.12%	3.61%
Rh	5.55%	2.90%
Pd	5.91%	2.55%
W	1.64%	3.08%
La	11.65%	3.81%
Ce	11.26%	4.22%

Table S5. Quantification of specific N-moieties; pyridinic, pyrrolic and graphitic and amine, for all metal free N-C catalysts.

**Catalyst**	**pyridinic N (At %)**	**pyrrolic (At %)**	**graphitic (At %)**	**Metal-N_x (At %)**
N-C (650-H_2 / 650-NH_3)	3.95%	3.35%	0.87%	2.03%
N-C (975-H_2 / 950-NH_3)	0.77%	1.03%	0.42%	0.59%
N-C (650-Ar / 650-Ar)	4.65%	3.59%	0.91%	2.16%
N-C (975-Ar / 950-Ar)	0.56%	0.96%	0.44%	0.67%

Table S6. Quantification of specific N-moieties; pyridinic, pyrrolic and graphitic and M-N_x, for all M-N-C catalysts.

Catalyst	pyridinic N (At %)	pyrrolic (At %)	graphitic (At %)	Metal-N_x (At %)
Cr	3.88%	3.36%	0.75%	1.75%
Mn	0.65%	0.79%	0.12%	0.69%
Fe	0.45%	0.66%	0.11%	0.43%
Co	0.58%	1.09%	0.24%	0.42%
Ni	0.55%	0.94%	0.22%	0.32%
Cu	0.77%	1.07%	0.38%	0.50%
Mo	0.23%	0.32%	0.04%	0.10%
Ru	3.21%	3.74%	0.89%	1.46%
Rh	0.75%	2.05%	0.55%	0.59%
Pd	0.72%	2.28%	0.51%	0.66%
W	0.12%	0.84%	0.18%	0.07%
La	3.37%	3.91%	0.80%	1.30%
Ce	3.34%	3.47%	0.69%	1.37%

Table S7. DFT calculated Gibbs free energy changes for reactions on Figure S55 (in eV) on different M-N_4 sites.

	**Descriptors for NH_3 formation (in NO_3RR)**		**Descriptors for NO_2^- formation (in NO_3RR) /NH_3 formation (in NO_2RR)**	
**site**	* + NO_3^- > *NO_3 + e^-	* + NO_3^- + $2H^+$ + e^- > *NO_2 + H_2O	*+ NO_3^- > *O + NO_2^-	*NO_2 + e^- > * + NO_2^-
	$\triangle_r G$ [eV]	$\triangle_r G$ [eV]	$\triangle_r G$ [eV]	$\triangle_r G$ [eV]
**FeN_4**	-0.39	-1.64	-0.35	+1.07
**MoN_4**	-2.72	-3.57	-4.20*	+3.0
**MoN_4-O**	-0.02	-0.42	-0.55	-0.15
**CuN_4**	+0.46	-0.47	+2.41	-0.11
**NiN_4**	+0.69	-0.44	+2.47	-0.13
**CoN_4**	-0.06	-1.60	+1.08	+1.03
**MnN_4**	-0.41	-1.52	-0.78	+0.95
**PdN_4**	+0.93	-0.17	+3.01	-0.39
**RuN_4**	-1.08	-2.08	-0.91	+1.52
**RhN_4**	+0.09	+0.19	+1.05	+0.97
**CrN_4**	-1.13	-1.85	-1.95	+1.28
**WN_4**	-3.15	-4.08	-4.63*	+3.52
**WN_4-O**	-0.16	-0.86	-1.10	+0.29
**LaN4**	-2.27	-2.82	-0.87	+2.25
**LaN_4-O**	-0.28	-0.60	1.79	0.02
**CeN4**	-2.39	-2.92	-2.47	+2.36
**CeN_4-O**	-0.82	-1.21	0.84	0.63
**pyridinic N**	+1.27	-0.02	+0.85	-0.55
**graphitic N**	+0.41	-0.34	+1.16	-0.33
**pyrrolic NH**	+0.52	-0.30	-2.29	-0.26

Table S8. Fitting parameters for the EXAFS spectra $\chi(k)k^2$ in R-space and Fourier transform k range.

	R min (Å)	R max (Å)	kmin ($Å^{-1}$)	k max ($Å^{-1}$)
Cr-N-C	1	2	2	8
Mn-N-C	1	2	3	8
Co-N-C	1	2	3	8
Ni-N-C	1	2	3	8
Cu-N-C	1	2	3	8
Rh-N-C	1	2	3	8
La-N-C	0.8	2.5	3	8
Ce-N-C	0.8	2.5	3	8
Fe-N-C	1	2	3	7.3

Table S9. DFT determined preferred *NO_3 and *NO_2 orientations on different M-N_4 sites.

	NO_3	***NO_2***
**FeN_4**	M-O (O…NC)	M-NO_2
**MoN_4**	M-OO (bidentate)	M-OO (bidentate)
**MoN_4-O**	M-OO (bidentate)	M-O (monodentate)
**CuN_4**	M-O (O…NC)	M-NO_2
**NiN_4**	M-O (O…NC)	M-NO_2
**CoN_4**	M-O (O…NC)	M-NO_2
**MnN_4**	M-OO (bidentate)	M-NO_2
**PdN_4**	M-O (O…NC)	M-NO_2
**RuN_4**	M-OO (bidentate)	M-NO_2
**RhN_4**	M-O (O…NC)	M-NO_2
**CrN_4**	M-OO (bidentate)	M-NO_2
**WN_4**	M-OO (bidentate)	M-OO (bidentate)
**WN_4-O**	M-OO (bidentate)	M-O (monodentate)
**LaN4**	M-OO (bidentate)	M-OO (bidentate)
**CeN4**	M-OO (bidentate)	M-OO (bidentate)
**pyridinic N**	CNC-OO (bidentate)	N-NO_2
**graphitic N**	CNC-NO_3 parallel	CC-OO (bidentate)
**pyrrolic NH**	NH-O (monodentate)	NH-O (monodentate)

Supplementary note 1: XANES analysis

For Cr-N-C, the XANES spectrum of the Cr K-edge exhibits a feature in the pre-edge at *ca.* 5994 eV, which strongly resembles the pre-edge of the K_2CrO_3 reference, a $Cr6^+$ compound. This feature arises due to the excitation of a photoelectron from the 1*s* state to the 3*d* state (such a transition is forbidden in Cr_2O_3).[1] The Cr oxidation state can be inferred by the relative intensity of this feature and can be used to quantify the ratio between Cr^{6+} (20-30%) and Cr^{3+} (70-80%).

For Mn-N-C, the XANES spectrum of the Mn K-edge the position and intensity of the main features closely resemble those in the MnO reference spectrum. Indicating Mn is in a Mn^{2+} oxidation state.

The Fe K-edge XANES spectrum for the Fe-N-C aligns well with the Fe_3O_4 reference spectrum, suggesting that Fe-N-C is in a *ca.* $Fe^{2.6+}$ oxidation state. While the Fe-N-C spectrum differs from Fe_3O_4 indicating a dissimilar local environment.

For Co-N-C, the XANES spectrum of the Co K-edge closely resembles that of the Co_3O_4 reference, which has an oxidation state of $Co^{2.6+}$. However, the Co-N-C is shifted to lower energies, indicating a slightly reduced oxidation state.

While the position of the Ni-N-C, Ni K-edge matches well with the Ni^{2+} reference, the white line intensity is reduced compared to the oxide reference. This behavior has also been observed for other atomically dispersed Ni-based catalysts containing a planar geometry with $Ni\text{-}N_4$ moieties.[2,3]

For Cu-N-C, the XANES spectrum of the Cu K-edge resembles the CuO reference spectrum, suggesting that Cu is in a Cu^{2+} oxidation state, exhibiting a 4-coordination or strongly disordered 6-coordination local environment.

For Mo-N-C, the XANES spectrum of the Mo K-edge is shifted to significantly higher energies than the Mo foil reference, suggesting a highly oxidized Mo state. Where the deconvoluted XPS spectrum supports Mo species present at high oxidation states, comprising a mixture of Mo^{6+}, Mo^{5+} and Mo^{4+}.

For Rh-N-C, the Rh-K edge XANES spectrum is very similar to the Rh_2O_3 reference both in the shape and intensity of the white line feature, indicating a Rh^{3+} oxidation state.

The Pd-N-C, the XANES spectrum of the Pd K-edge is shifted to higher energies than the Pd reference foil suggesting the Pd-N-C is in an oxidized state. Where the deconvoluted XPS spectrum suggests a mixture of Pd^{2+} and Pd^{0}.

For La-N-C, the XANES spectrum of the La L_3-edge is similar to that of the amorphous La_2O_3 reference, suggesting a La oxidation state of La^{3+}. The energy calibration was done by collecting the V K-edge spectrum for a vanadium foil as a reference, which was measured simultaneously with the La-N-C sample.

For Ce-N-C, the XANES spectrum of the Ce L_3-edge differs strongly from the CeO_2 reference spectrum. However, the Ce-N-C spectrum is very similar to Ce^{3+} compounds in the literature, for example, cerium nitrate, $Ce(NO_3)_3$.[4] Therefore suggesting the Ce-N-C catalyst is in a *ca.* Ce^{3+} oxidation state.

Note several references were acquired from XAS databases.

Cr : K_2CrO_3 and Cr_2O_3 references are extracted from the Hokkaido university XAS database.[5]

La: The La_2O_3 reference data was extracted from the Farrel Lytle XAS data base. (http://ixs.iit.edu/database/data/Farrel_Lytle_data/RAW/index.html)

Ce: The CeO_2 reference was taken from the HEPHAESTUS data base.[6]

Note that due to some problems with the optics at the beamline, the optimal energy resolution was not obtained at the KMC-3 beamline (BESSY), which can lead to some distortions in the spectra. The samples measured at BESSY include Cr-, Mn-, Co-, Ni-, Cu- and Ce-N-C. However, corresponding reference foils and compounds were measured for energy calibrations and comparison of features. The XANES spectra for Mo-N-C and Pd-N-C and corresponding reference metal foils were measured on the 10-BM beamline of the Advanced Photon Source are Argonne National Laboratory.

Supplementary note 2: Optimization of catalyst synthesis conditions

To accurately evaluate the NO_2RR/NO_3RR activity and selectivity of single-atom centers, synthesis parameters were optimized to ensure the exclusive formation of $M\text{-}N_x$ sites, especially in metals difficult to disperse atomically, such as 4d noble metals (Ru, Rh and Pd) and rare earth elements (La and Ce) (Table S1). Therefore, control experiments to deconvolute the contributions of the metal-free N-C supports for the NO_3RR and NO_2RR revealed similar activity between all metal free N-C’s, regardless of the pyrolyzing conditions (Figure S39 and S46),

indicating the minimum impact on the NO_3RR and NO_2RR activity by the non-metal-associated N-moieties, as discussed in more detail in the main text.

Supplementary note 3: EXAFS analysis - coordination of M-N-C catalysts

The observed coordination number for the M-N-C catalysts varies between *ca.* 1.8 to 8.4. While Rh-N-C displays a coordination number of 4 (that anticipated for $M-N_4$ motifs), for Fe- and Cu-N-C, it is likely that in addition to the M-N bonds, some additional M-O bonds are present, leading to a slightly increased coordination number. For Ni-N-C, the low coordination number could be attributed to the large structural disorder due to the off-center displacement of the Ni for the center of the $Ni-N_4$ moiety (as indicated by the XANES features). Additionally, Ce-N-C, in addition to the M-N feature at low bond distance, the wavelet transformations of the EXAFS data also show an additional wave peak at higher R- and k-values, indicating small oxide clusters not detected by other techniques may co-exist.

Supplementary note 4: Limitations of the computational approach and the difficulties in modelling M-N-C catalysts

M-N-C catalysts are challenging to simulate, and the reliability of the computational results depends on many key-factors. For example, parameters used in the Density Functional Theory (DFT) calculations such as number of k-points, spin polarization, dispersion correction, and choice of the exchange-correlation functional can affect computational results.[7] In particular, there is evidence of deviations for high spin complexes when comparing results obtained using standard generalized gradient approximation and self-interaction corrected functionals. In addition, modeling of M-N-C catalysts represents a challenge as these materials lie at the boundary between homogeneous and heterogenous catalysts. Like homogeneous catalysts, metal atoms in M-N-C catalysts are individual atoms, whose chemical reactivity directly relates to their electron configuration. However, similarly to heterogeneous catalysts, metal atoms incorporated into graphene have a strong interaction to the underlying support, which in turns influences their oxidation state. Oxidation state will also depend on the adopted functional, which affects the degree of electron density localization and consequently the amount of charge on a metal atom in the M-N-C catalysts. Moreover, the real operating conditions in electrocatalysis are far from the vacuum conditions assumed in our DFT calculations and a

realistic modelling should account for solvent effects. Accounting for all these effects is extremely challenging[8]; however, interpretation of relative values and trends while cross-validating DFT results with experimental findings still provide reasonable and valuable insight.

Supplementary References

1. Peterson, M. L., Brown, G. E., Parks, G. A. & Stein, C. L. Differential redox and sorption of Cr(III/VI) on natural silicate and oxide minerals: EXAFS and XANES results. *Geochim. Cosmochim. Acta* **61**, 3399–3412 (1997).
2. Li, C. *et al.* Covalent Organic Framework (COF) Derived Ni-N-C Catalysts for Electrochemical CO 2 Reduction: Unraveling Fundamental Kinetic and Structural Parameters of the Active Sites . *Angew. Chemie* **134**, (2022).
3. Zhou, M. *et al.* Single-atom Ni-N4 provides a robust cellular NO sensor. *Nat. Commun.* **11**, 1–9 (2020).
4. Smolders, S. *et al.* Unravelling the Redox-catalytic Behavior of Ce4+ Metal–Organic Frameworks by X-ray Absorption Spectroscopy. *ChemPhysChem* **19**, 373–378 (2018).
5. Asakura, K., Abe, H. & Kimura, M. The challenge of constructing an international XAFS database. *J. Synchrotron Radiat.* **25**, 967–971 (2018).
6. Ravel, B. & Newville, M. ATHENA, ARTEMIS, HEPHAESTUS: Data analysis for X-ray absorption spectroscopy using IFEFFIT. *J. Synchrotron Radiat.* **12**, 537–541 (2005).
7. Di Liberto, G., Cipriano, L. A. & Pacchioni, G. Universal Principles for the Rational Design of Single Atom Electrocatalysts? Handle with Care. *ACS Catal.* **12**, 5846–5856 (2022).
8. Tosoni, S., Di Liberto, G., Matanovic, I. & Pacchioni, G. Modelling single atom catalysts for water splitting and fuel cells: A tutorial review. *J. Power Sources* **556**, 232492 (2023).
